# Impact of hypertensive heart disease, risk factors, and age-period-cohort models across 204 nations and regions from 1990 to 2019: a global perspective from the 2019 global burden of disease study

**DOI:** 10.3389/fcvm.2024.1417523

**Published:** 2024-07-18

**Authors:** Guoliang Gao, Zhaoyi Chen, Guoping Yan, Minqiang Bao

**Affiliations:** ^1^ Department of Electrophysiology, Xuancheng People’s Hospital, Affiliated Xuancheng Hospital of Wannan Medical College, Xuancheng, Anhui, China; ^2^ Department of Gastroenterology, Xuancheng People’s Hospital, Affiliated Xuancheng Hospital of Wannan Medical College, Xuancheng, Anhui, China; ^3^ Department of Neurology, Xuancheng People’s Hospital, Affiliated Xuancheng Hospital of Wannan Medical College, Xuancheng, Anhui, China

**Keywords:** 2019 global burden of disease study, hypertensive heart disease, age-period-cohort analysis, risk factors, mortality rates, prevalence trends, sociodemographic factors

## Abstract

**Background:**

Hypertensive heart disease (HHD) is a major global public health issue resulting from hypertension-induced end-organ damage. The aim of this study was to examine the global impact, risk factors, and age-period-cohort (APC) model of HHD from 1990 to 2019.

**Methods:**

Data from the 2019 Global Burden of Disease were used to assess age-adjusted HHD prevalence, disability-adjusted life years (DALYs), mortality rates, and contributions of HHD risk factors with 95% uncertainty intervals (UIs). APC models were used to analyze global age, period, and cohort mortality trends for HHD.

**Results:**

In 2019, 18.6 million prevalent HHD cases led to 1.16 million fatalities and 21.51 million DALYs. Age-adjusted rates were 233.8 (95%UI = 170.5–312.9) per 100,000 individuals for prevalence, 15.2 (11.2–16.7) for mortality, and 268.2 (204.6–298.1) for DALYs. Regionally, the Cook Islands (703.1), Jordan (561.6), and Kuwait (514.9) had the highest age-standardized incidence of HHD in 2019. There were significant increases in HHD prevalence in Andean Latin America (16.7%), western sub-Saharan Africa (5.6%), and eastern sub-Saharan Africa (4.6%). Mortality rate varied widely among countries. Risk factors like elevated systolic blood pressure and high body mass index significant influenced DALY rates, especially in females. The APC model revealed an association between mortality rates and age, with a decreasing mortality risk over time and improved survival rates for a later birth cohort.

**Conclusions:**

Despite the reduction in prevalence, HHD remains a significant public health issue, particularly in nations with low sociodemographic indices. To alleviate the impact of HHD, prevention efforts should concentrate on the management of hypertension, weight loss, and lifestyle improvement.

## Introduction

Hypertensive heart disease (HHD) manifests as a sequence of pathological alterations in the heart, predominantly impacting the left ventricle, a significant organ-damaging outcome of hypertension (HTN), and posing global health and quality of life concerns (1). HHD plays a crucial role in the development of cardiovascular diseases and death rates (2). HHD ranks as the fourth leading cause of cardiovascular mortality, following ischemic heart disease, stroke, and cardiomyopathy (3). Hypertension is typically defined as a mean systolic blood pressure (SBP) of ≥140 mm Hg and diastolic blood pressure (DBP) of ≥90 mm Hg, or by the self-reported use of antihypertensive medications. HHD is a serious condition that significantly increases the risk of cardiovascular, cerebrovascular, and renal diseases and other systemic diseases. It is estimated that 1.4 billion people worldwide have high blood pressure, yet only 14% manage to keep it under control (4).

## Materials and methods

### Study data

The foundation of our research is the 2019 GBD study. The Global Health Data Exchange website (http://ghdx.healthdata.org/gbd-results-tool) [Global Health Data Exchange (GHDx) query tool (Accessible at: http://ghdx.healthdata.org/gbd-results-tool) hosts all anonymized data for public use. The IHME website (http://ghdx.healthdata.org/] provided the Socio-demographic Index (SDI) information for the 21 areas of GBD. Investigating possible signs of the worldwide impact of HHD, we examined information from various origins. Initially, data related to sex and age were collected to evaluate their effect on the burden of HHD. Subsequently, the data underwent a reevaluation based on geographic locations within 21 GBD regions. Ultimately, the prevalence of HHD across 204 nations and regions was depicted through tabular data and global maps (5).

### Definition

#### HHD

HHD, a heart condition, arises from continuously high blood pressure affecting the left ventricle, left atrium, and intramural coronary arteries (6, 7). HHD manifests as symptoms of heart failure, originating from the effects of long-term hypertension (8). LVH, among the initial symptoms of HHD, is believed to serve as a balancing process to reduce the escalation of ventricular wall stress, a transitional pathological alteration in the development of HHD (9). HHD is defined by the International Classification of Diseases (ICD)-9 (code 40–402.9) and ICD-10 (code I11–I11.9) (10).

### Disability-adjusted life-years

The calculation of Disability-adjusted life years (DALYs) involves adding up the number of years lost from premature death (YLL) to the years spent living with disability (YLD). The calculation of YLLs involves multiplying the death rate per 100,000 people by the worldwide average lifespan at the time of death. YLDs are calculated by summing the frequency and length of the disease or injury, taking into account the illness's severity (known as the disability weight), which varies from 0 (optimal health) to 1 (lethal severity). A single DALY, being the aggregate of YLLs and YLDs, signifies the loss of a year's healthy lifespan in the population. Comprehensive data regarding DALY, YLL, and YLD calculations, encompassing techniques to evaluate the comparative morbidity and mortality rates of specific diseases and injuries, along with disability weights, are provided in other publications (10).

### The ASR

The ASR, or the age-standardized rate, encapsulates the observed rate in populations with a uniform age distribution. When assessing various age groups across populations, standardization is essential, achieved by transforming the real age composition rate of the population into a standardized age structure rate. Weights are calculated using the standard population distribution, and the ASR (per 100,000 people) is formulated as follows: ai represents the specific age ratio of group i, and wi denotes the count or weight of the chosen reference standard population group i.

$$\mathrm{ASR}=\frac{\sum_{i=1}^{A}a_{i}w_{i}}{\sum_{i=1}^{A}w_{i}}\times 100\;000$$

### The SDI

This broad metric determines the role of a nation or another geographical region in the context of development. Measured on a scale from 0 to 1, the SDI represents an aggregated mean of per capita earnings, average educational achievements, and fertility rates across the regions included in the GBD study.

### Age-Period-Cohort (APC) model

The age-period-cohort (APC) model is a widely used statistical tool in epidemiology for disentangling the effects of age, time period, and birth cohort on disease trends. Given the multifaceted nature of HHD, where age-related biological factors, temporal changes in medical practices and lifestyle, and generational shifts in risk factors all play crucial roles, the APC model provides a robust framework to analyze these dynamics. The selection of the APC model for this study was based on several strengths. (1) Separating the effects of age, period, and cohort can help to distinguish the influence of aging (age effect), changes over time (period effect), and generational differences (cohort effect) on HHD mortality trends. (2) The APC model can aid in the identification of temporal patterns. By examining the period and cohort effects, the APC model aids in identifying shifts in disease risk and mortality over time, which can be linked to advancements in medical care, public health interventions, and changes in risk factor prevalence. (3) The APC model can also be used to support public health interventions, as understanding these patterns enables policymakers to design targeted interventions for specific age groups or generations, improving the overall effectiveness of public health strategies.

### Risk factors

The GBD 2019 offers global and regional evaluations of attributable mortality and disability-adjusted life years (DALYs) associated with 87 risk factors and their combinations. The comparative evaluation of the risk assessment framework (RAF) is utilized to determine the agreement between mortality and DALYs linked to established risk factors (10), and risk factors are classified into four separate groups. The initial level consists of three types of risk factors (behavioral, occupational and metabolic, and environmental), which are then divided into 20 Tier 2 risk factors (10), with further details of the framework provided in a different section. Details regarding this structure have been previously published (10).

### Statistical analysis

To evaluate shifts in the HHD burden, we employed metrics like death count, DALYs, the ASR, the age-adjusted DALY rate, and the related APC model. Every statistical evaluation was conducted using R software (version 4.3.3), with a *P*-value less than 0.05 considered to indicate statistical significance.

### Outcome assessment

Utilizing the global standardized disease burden life tables, the death count for each age category was multiplied by their residual life expectancy to calculate the lost life years. To calculate the DALYs, the total years of death and disability were aggregated. The levels of uncertainty were determined by conducting 1,000 samplings at each stage of calculation and amalgamating uncertainties from various sources, including input data, adjustments for measurement inaccuracies, and calculations of remaining nonsampling errors. Uncertainty intervals were identified as the 25th and 975th values in the sequential plot. A smoothed spline model was employed to investigate the link between the HHD burden (DALYs) and sociodemographic measures across 21 regions and 204 countries and territories (11). Spanning from 0 (least developed) to 1 (most developed), the sociodemographic index serves as an aggregate measure of delayed per capita dependent income, encompassing per capita gross domestic product (GDP) (calibrated across the past decade), the average educational duration of the populace (>15 years), and the total fertility rate (TFR) for those under 25 years. Using R software (version 4.3.2), we charted the age-adjusted rates of point prevalence, mortality, and DALYs. The results of the analysis of mortality data through APC modeling are presented below. In this research, we employed the APC model to examine the fundamental patterns in mortality across different ages, periods, and birth cohorts (12). The APC framework aims to simplify the impact of age-related biological factors, along with technological and social aspects, on disease patterns, extending beyond conventional epidemiological studies (13). This method has been applied in detailed epidemiological studies on various chronic illnesses, including cardiovascular disease (14). Typically, APC models involve the application of a log-linear Poisson model to Lexis plots showing observed rates and are used to measure the aggregate impact of age, time period, and birth cohort. Given the perfectly linear correlations among age, period, and cohort (birth cohort = period−age), statistically assessing their separate impacts, known as the identification problem, is unfeasible (12, 13). The APC approach circumvents this issue by creating estimable APC numbers and functions, avoiding the imposition of random limitations on the model's parameters (12). The APC model can be implemented by using a no-cost R tool; it's the methodological specifics for this analysis are outlined in an earlier study (15). In this study, the 2019 GBD estimates of HTN-related heart-disease deaths and demographic information from each nation/region were incorporated into the APC model. Within a standard APC framework, all age and period intervals must be identical, meaning that age categories of five years should be paired with five-year calendar periods. Given that GBD estimations are produced using a data structure with irregular intervals (with five-year age groups and yearly data), we organized the GBD data into one cell frame, choosing death and population figures for each of the six five-year periods (namely, [1992] 1990–1994, [1997] 1995–1999…[2017] 2015–2019) to denote distinct timeframes. The collected data included 14 different 5-year age groups (spanning from 0 to 4 years old to 65–69 years old) along with 19 cohorts born every ten years, with mid-year birth records indicating a range from 1921 to 1929 (1925) to 2011 to 2019 (2015). The adapted APC model calculated the general temporal pattern of mortality, denoted as the yearly percentage variation in mortality rates (that is, the net shift in mortality, % annually). From a technical standpoint, the overall drift was ascertained through two distinct factors: a trend factor linked to calendar time and another linked to consecutive cohorts. Additionally, the APC model predicts the temporal progression of mortality across different age groups, quantified by the yearly percentage variation in mortality rates specific to each age group (that is, the local shift in mortality rates, percentage annually), mirroring the patterns in birth cohort impacts (15). An annual shift exceeding ±1% is deemed a notable alteration in death rates (15), accounting for approximately ±10%, ±18%, and ±26% of the variations in adjusted rates across 10-, 20-, and 30-year intervals, respectively. All analyses were conducted using R (version 4.3.2).

## Results

### Global results

In 2019, 18.6 million cases of HHD (refer to Table 1), averaging an age-adjusted rate of 233.8 cases per 100,000 people, were reported worldwide, demonstrating a 6.5% increase from 1990. In 2019, 1.16 million HHD-related deaths occurred, accompanied by an age-adjusted mortality rate of 15.2, marking a 21.5% reduction since 1990. As of 2019, the worldwide DALY rate for HHD stood at 21.51 million, and the age-adjusted rate of DALYs per 100,000 people was 268.2, demonstrating a 26.4% reduction from 1990 (Table 1).

**Table 1:** Hypertension- and heart-disease-related cases, deaths, modified life years (DALYs), and fluctuations in age-adjusted rates (ASRs) per 100,000 people from 1990 to 2019, categorized by global disease burden regions.

	Prevalence (95% UI)			Deaths (95% UI)			DALYs (95% UI)		
	No, in Millions (95% UI)	ASRs per 100,000 people (95% UI)	Percentage change in ASRs from 1990 to 2019	No, in Thousands (95% UI)	ASRs per 100,000 people (95% UI)	Percentage change in ASRs from 1990 to 2019	No, in Thousands (95% UI)	ASRs per 100,000 people (95% UI)	Percentage change in ASRs from 1990 to 2019
Global	18.6 (13.5 to 24.9)	233.8 (170.5 to 312.9)	6.5 (3.6 to 9.8)	1156.7 (859.8 to 1,278.6)	15.2 (11.2 to 16.7)	−21.5 (−35.2 to −10.1)	21,508 (16,400.1 to 23,899.9)	268.2 (204.6 to 298.1)	−26.4 (−35.5 to −15.7)
High-income Asia Pacific	0.4 (0.3 to 0.6)	85.1 (61.5 to 114.6)	−9.7 (−21.8 to 5)	19.3 (14.6 to 23.7)	3 (2.3 to 4)	−66.8 (−72.6 to −27.5)	231.3 (187.4 to 320.8)	44 (36.5 to 64.6)	−65.9 (−71 to −28.6)
High-income North America	1.5 (1.1 to 2)	253.1 (188.8 to 330.6)	3.1 (−9.7 to 18)	54.5 (36.4 to 59.5)	8.3 (5.4 to 9)	21.4 (−15.5 to 28)	1,102 (735.5 to 1,201.1)	193.7 (126.5 to 210.2)	24.7 (−14.5 to 32.4)
Western Europe	1.3 (0.9 to 1.8)	123.5 (89.2 to 169.2)	0.1 (−6.4 to 7.6)	101.5 (68.5 to 115.2)	8.4 (5.8 to 9.5)	4.5 (−31.7 to 15.3)	1,123 (847.3 to 1,253.2)	103.5 (80.7 to 115.7)	−15.1 (−34 to −4.2)
Australasia	0 (0 to 0.1)	79.1 (57.2 to 106.2)	−31.4 (−37 to −25.7)	1.5 (1.1 to 1.7)	2.5 (2 to 3)	−29.2 (−37 to −4.2)	20.4 (16.9 to 24.2)	38.9 (32.9 to 46.6)	−35.6 (−41 to −9.9)
Andean Latin America	0.1 (0.1 to 0.1)	138.4 (95.1 to 196.9)	16.7 (7.6 to 28.9)	5.2 (4.2 to 6.1)	9.7 (8 to 11.5)	−25.2 (−40.8 to −9.5)	90.9 (74.6 to 108.3)	164.6 (134.9 to 195.7)	−29.1 (−43.2 to −13.3)
Tropical Latin America	0.4 (0.3 to 0.6)	171 (118.4 to 242.8)	−1.6 (−5 to 2.4)	30.7 (26.7 to 41.7)	13.4 (11.6 to 18.2)	−38.6 (−44.3 to −7.7)	572.4 (515.6 to 781.2)	240.4 (215.7 to 327.9)	−42.1 (−47.6 to −8.8)
Central Latin America	0.3 (0.2 to 0.5)	153.5 (109 to 216.1)	−4 (−7.2 to −0.5)	23.6 (19.4 to 29.5)	10.7 (8.8 to 13.3)	−38.4 (−47.9 to −4.6)	392.5 (329.2 to 503.1)	171.5 (144.2 to 219.6)	−40.6 (−50.1 to −6.3)
Southern Latin America	0.2 (0.1 to 0.2)	184 (127 to 265.4)	−9.5 (−17.2 to −1.1)	12 (9.8 to 13.7)	13.9 (11.4 to 15.9)	−2.6 (−22.7 to 8.4)	175.5 (153.8 to 209.6)	207 (182.3 to 247.8)	−17.8 (−26.8 to −7.8)
Caribbean	0.1 (0.1 to 0.1)	202.5 (147.9 to 279.7)	−2.8 (−6.5 to 1.5)	9.7 (8 to 11.5)	18.7 (15.4 to 22.1)	−3.5 (−19.5 to 11)	192.4 (158.2 to 229.2)	372.3 (306.2 to 444.1)	−4.2 (−20.1 to 12.6)
Central Europe	0.4 (0.3 to 0.6)	173.8 (123.5 to 242)	−11.9 (−19.6 to −2.9)	48.7 (34.9 to 56.4)	21.7 (15.5 to 25.1)	13.4 (−26.2 to 30.5)	730.9 (546.3 to 846.7)	334.8 (249.4 to 387.4)	2 (−28.4 to 17.6)
Eastern Europe	0.2 (0.1 to 0.3)	56.5 (38.2 to 83)	0.7 (−2.5 to 3.4)	24.6 (15.1 to 28.5)	7.1 (4.4 to 8.2)	90.8 (−10.5 to 125.8)	447.2 (297.6 to 517.3)	131.7 (88.5 to 152.6)	59.3 (−6.5 to 85.1)
Central Asia	0.1 (0.1 to 0.2)	190.2 (133.2 to 262.4)	−9.2 (−14.9 to −4.3)	14.5 (12.3 to 16.5)	27.8 (21.9 to 31.6)	70.6 (16.9 to 110.6)	292.5 (252.6 to 335)	454.6 (390 to 515.3)	39.7 (8.2 to 68.9)
North Africa and Middle East	1.3 (1 to 1.8)	333.9 (249.1 to 446.5)	2.7 (−2.2 to 8.3)	108.9 (59.8 to 135.2)	32.2 (17.1 to 39.3)	−21.7 (−41.8 to −2.7)	2,180.4 (1,285.2 to 2,768.7)	545 (315.8 to 682.4)	−25 (−42 to −7.4)
South Asia	1.3 (0.9 to 1.9)	111.8 (78.3 to 159)	1.5 (−0.6 to 3.5)	146.3 (103.8 to 191.5)	13.2 (9.3 to 17.4)	−30.9 (−45.8 to −5)	3,002.4 (2,193.4 to 3,898.3)	229.2 (166.1 to 296.9)	−31.4 (−44.7 to −5)
Southeast Asia	1.8 (1.3 to 2.5)	334.8 (244.8 to 451.6)	−0.1 (−3.3 to 3.4)	110.4 (70.7 to 124.5)	21.4 (14.2 to 24)	−14.5 (−30.1 to 4.5)	2,498.9 (1,585.1 to 2,834.2)	422.9 (274.2 to 476.8)	−15.1 (−29.6 to 4.1)
East Asia	8.1 (5.8 to 11)	426.1 (306.6 to 574.8)	−5.9 (−8.6 to −2.8)	330.5 (211.8 to 384.6)	20.3 (12.8 to 23.5)	−50.9 (−64.5 to −40.3)	5,780.8 (4,053.6 to 6,713.5)	310.4 (217.5 to 359.4)	−54.4 (−63.6 to −44.7)
Oceania	0 (0 to 0)	344.9 (248.5 to 477.9)	1.1 (−4.8 to 7.8)	1.7 (1.1 to 2.3)	28.5 (19.5 to 37.7)	−9.4 (−25.3 to 10.8)	46.4 (29.9 to 63.5)	626.3 (419.1 to 841.6)	−8.8 (−25.9 to 13.6)
Western sub-Saharan Africa	0.4 (0.3 to 0.5)	242.9 (168.7 to 330.3)	5.6 (2.2 to 8.9)	29.6 (18.1 to 37.3)	18.9 (11.8 to 23.5)	2.1 (−37.5 to 28.9)	761.6 (475.9 to 972.6)	388.9 (247.3 to 487.7)	0.2 (−36.7 to 26.8)
Eastern sub-Saharan Africa	0.4 (0.3 to 0.5)	281.8 (193.7 to 387.8)	4.6 (1.6 to 7.6)	47.5 (27.9 to 71.8)	38.8 (22.4 to 58.5)	−24.6 (−37.5 to −4.7)	1,077.4 (658.7 to 1,587.1)	696.9 (418.4 to 1,038.7)	−31.2 (−43.4 to −9.5)
Central sub-Saharan Africa	0.1 (0 to 0.1)	147.6 (95.6 to 222.5)	−1.6 (−8.6 to 5.5)	20.7 (13.3 to 28.1)	53.5 (34.3 to 72.5)	−8.9 (−27 to 13.7)	479.6 (307.6 to 648.1)	970.3 (625.8 to 1,310.4)	−15.1 (−33.3 to 7.3)
Southern sub-Saharan Africa	0.1 (0 to 0.1)	147.8 (95.8 to 216)	−9.2 (−13.1 to −5.4)	15.2 (13.5 to 17.1)	34.1 (30.1 to 38.1)	7.8 (−10.6 to 20.2)	309.5 (277.9 to 349.1)	580.3 (519.5 to 648.9)	−4.7 (−17.5 to 5.9)

DALYs, disability-adjusted life-years; ASRs, age-standardized rates; 95% UI, 95% uncertainty intervals.

### Regional results

The year 2019 had the highest age-adjusted incidence of HHD (per 100,000 people) in East Asia (426.1), Oceania (344.9), and Southeast Asia (334.8), with the lowest rates in the affluent Asia-Pacific region (85.1), Australasia (79.1), and Eastern Europe (56.5) (Table 1). In 2019, the peak age-standardized death rates for HHD were observed in sub-Saharan Central Africa (53.5), sub-Saharan Eastern Africa (38.8), and sub-Saharan Africa (34.1), with Australasia (2.5), the high-income Asia-Pacific region (3.0), and Eastern Europe (7.1) having the highest rates (Table 1). In contrast, sub-Saharan Central Africa (970.3), sub-Saharan Eastern Africa (696.9), and Oceania (626.3) had the highest age-standardized death rates per 100,000 individuals, whereas Australasia (38.9), the high-income Asia-Pacific region (44.0), and Western Europe (103.5) had the lowest rates (Table 1).

Between 1990 and 2019, Andean Latin America (16.7%), western sub-Saharan Africa (5.6%), and eastern sub-Saharan Africa (4.6%) had the fastest increase in age-adjusted cases of HHD, while Australasia (−31.4%), Central Europe (−11.9%), and the Asia-Pacific region (−9.7%) had the most significant decreases (Table 1). Concurrently, Eastern Europe (90.8%), Central Asia (70.6%), and affluent North America (21.4%) experienced the most significant increases in age-adjusted death rates due to HHD. In contrast, wealthier regions such as the Asia-Pacific region (−66.8%), East Asia region (−50.9%), and Tropical Latin America region (−38.6%) experienced the most substantial reductions in these rates (Table 1).

Between 1990 and 2019, the most significant increases in DALY rates were observed in Eastern Europe (59.3%), Central Asia (39.7%), and affluent North America (24.7%), whereas Asia-Pacific (−65.9%), East Asia (−54.4%), and Tropical Latin America (−42.1%) experienced the most substantial decreases in these rates (see Table 1).

The incidence of HHD increased from 7.817 million in 1990 to 18.6 million in 2019. In 1990, the greatest number of outbreaks were documented in East Asia, China, and North America, with East Asia, China, and Southeast Asia leading in 2019 (Supplementary Table S1). Fatalities due to HHD surged from 650,000 in 1990 to 1.16 million in 2019, with East Asia, China, and South Asia accounting for the most deaths in 2019 (Supplementary Table S2). The incidence of DALYs linked to HTN and heart disease increased from 13.94 million in 1990 to 21.5 million in 2019, with East Asia, China, and South Asia accounting for the greatest number of DALYs in 2019 (Supplementary Table S3).

### National results

**Figure 1:**
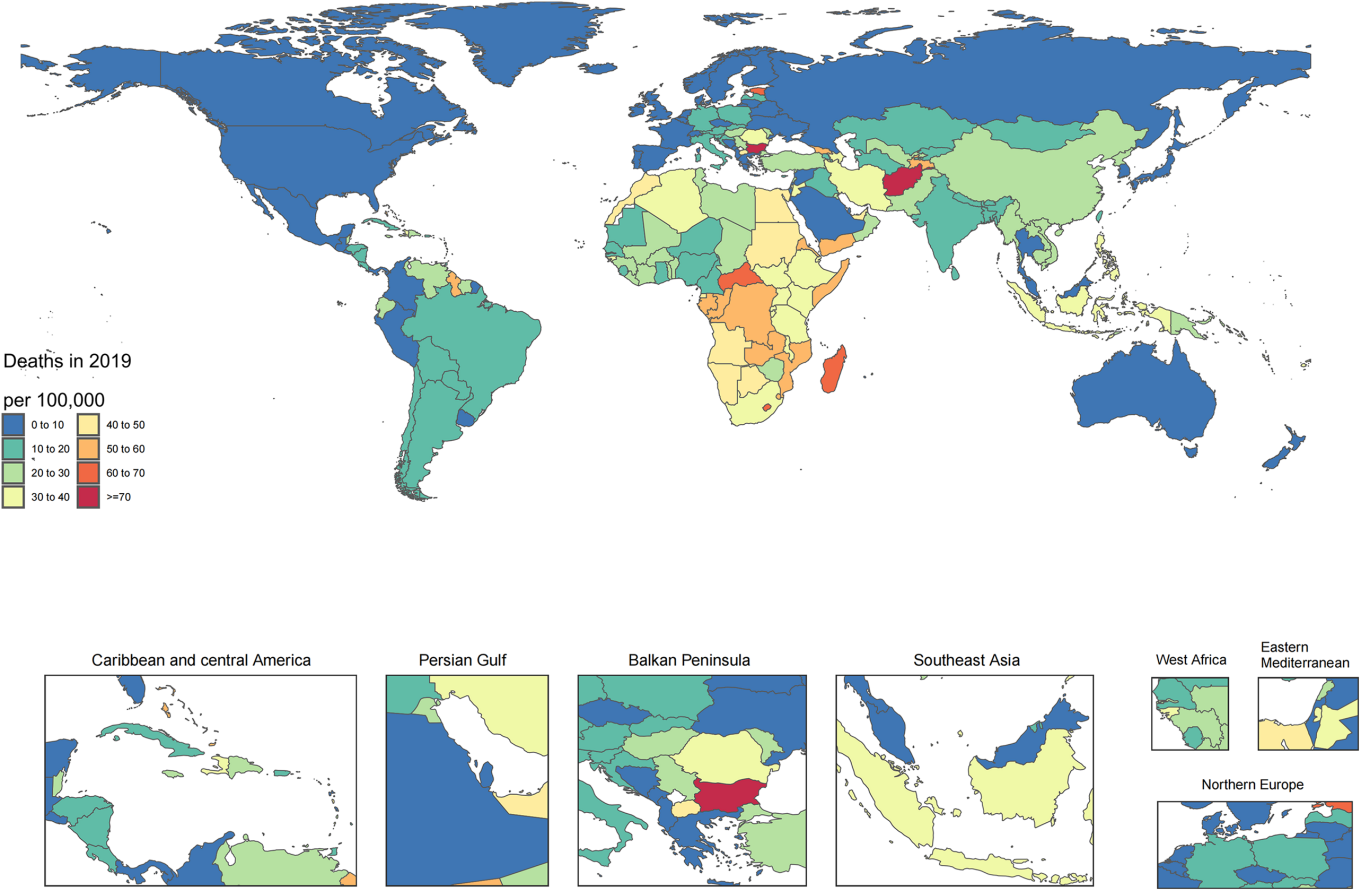
Age-standardized point prevalence of hypertension and heart disease per 100,000 people in 2019, categorized by country.

**Figure 2:**
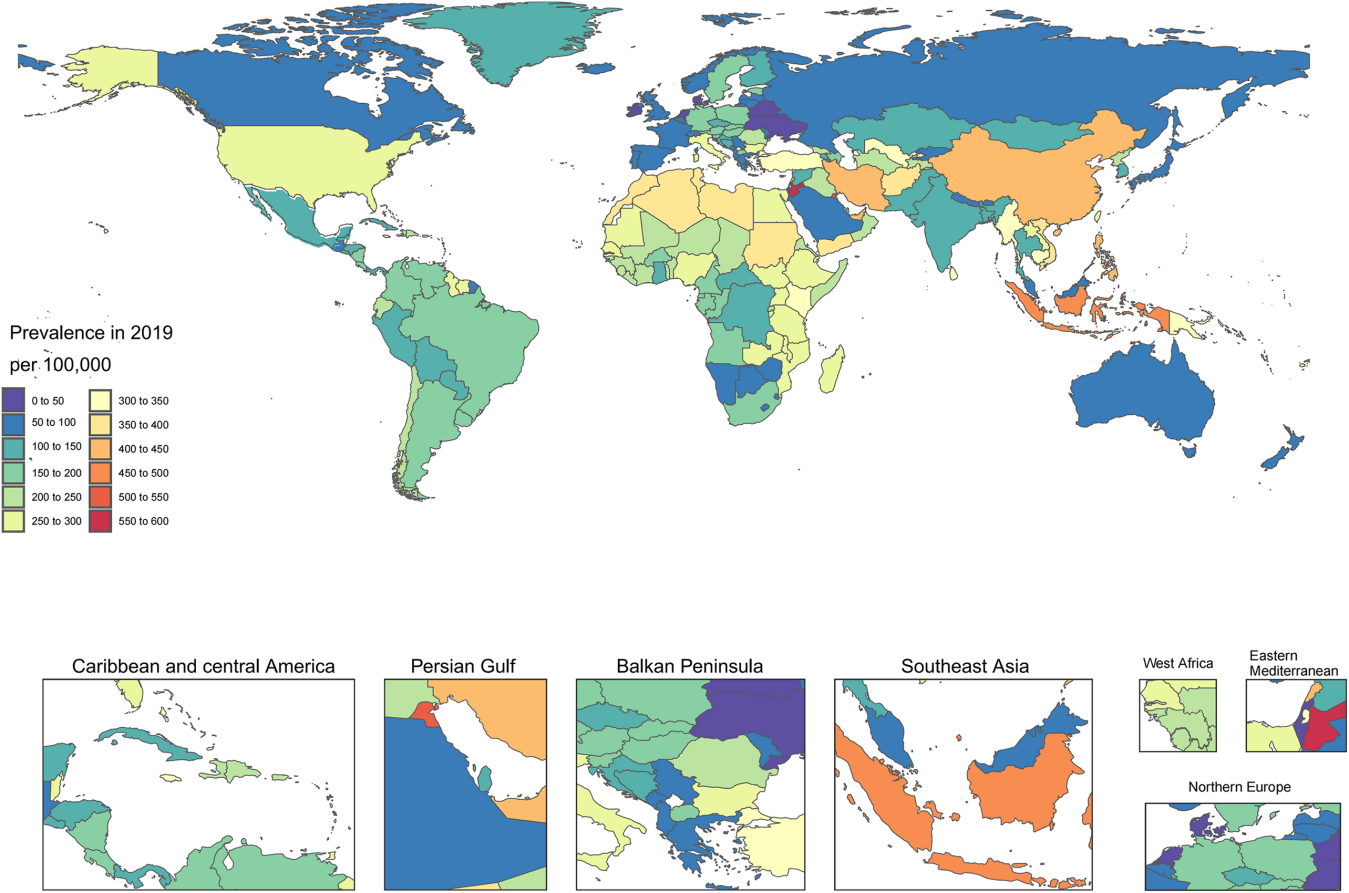
Age-standardized mortality rate for hypertension and heart disease per 100,000 people in 2019, categorized by country.

In 2019, the age-adjusted incidence of HHD among the nations varied between 11.9 and 703.1 cases per 100,000 individuals. In 2019, the Cook Islands (703.1), Jordan (561.6), and Kuwait (514.9) had the greatest age-adjusted incidence of HHD, in contrast to Ukraine (11.9), Belarus (28.4), and Denmark (30.6), which had the lowest incidence (Figure 1; Supplementary Table S1). The national age-adjusted death rate from HTN and heart disease increased from 1 to 75 deaths per 100,000 individuals. Bulgaria, Afghanistan, and the Central African Republic had the highest mortality rates, in contrast to Israel, Ukraine, and Norway, which had the lowest mortality rates (Figure 2; Supplementary Table S2). In 2019, the national age-adjusted DALY rates for HTN and heart disease varied between 27.8 and 1,374.1 per 100,000 individuals. The peak rates were recorded for Afghanistan (1,374.1), the Cook Islands (1,356.2), and the Central African Republic (1,321.8), while the rates lowest rates occurred in Israel (27.8), North China (28.0), and Denmark (30.6) (Supplementary Figure S1; Tables S3).

Age-adjusted prevalence percentages from 1990 to 2019 significantly differed by nation, with the most substantial increases observed in Bolivia (103.3%), Italy (42.9%), and Cameroon (40.6%). Conversely, Serbia (−40.3%), France (−38.4%), and Croatia (−38.3%) had the most significant reductions (Supplementary Table S1). Concurrently, Estonia (695.5%), Latvia (515.7%), and the Republic of Moldova (485.9%) experienced the most significant increases in age-adjusted death rates, whereas Israel (−75.7%), the Gulf (−7.3%), and Japan (−69.8%) experienced the most substantial decreases (Supplementary Table S2). From 1990 to 2019, Estonia (473.0%), Latvia (420.1%), and the Republic of Moldova (340.0%) experienced the most substantial growth in age-standardized DALY rates for HHD. Conversely, Israel (−76.7%), the Republic of Korea (−72.1%), and Colombia (−66.9%) experienced the most significant declines (see Supplementary Table S3).

### Age and sex patterns

**Figure 3:**
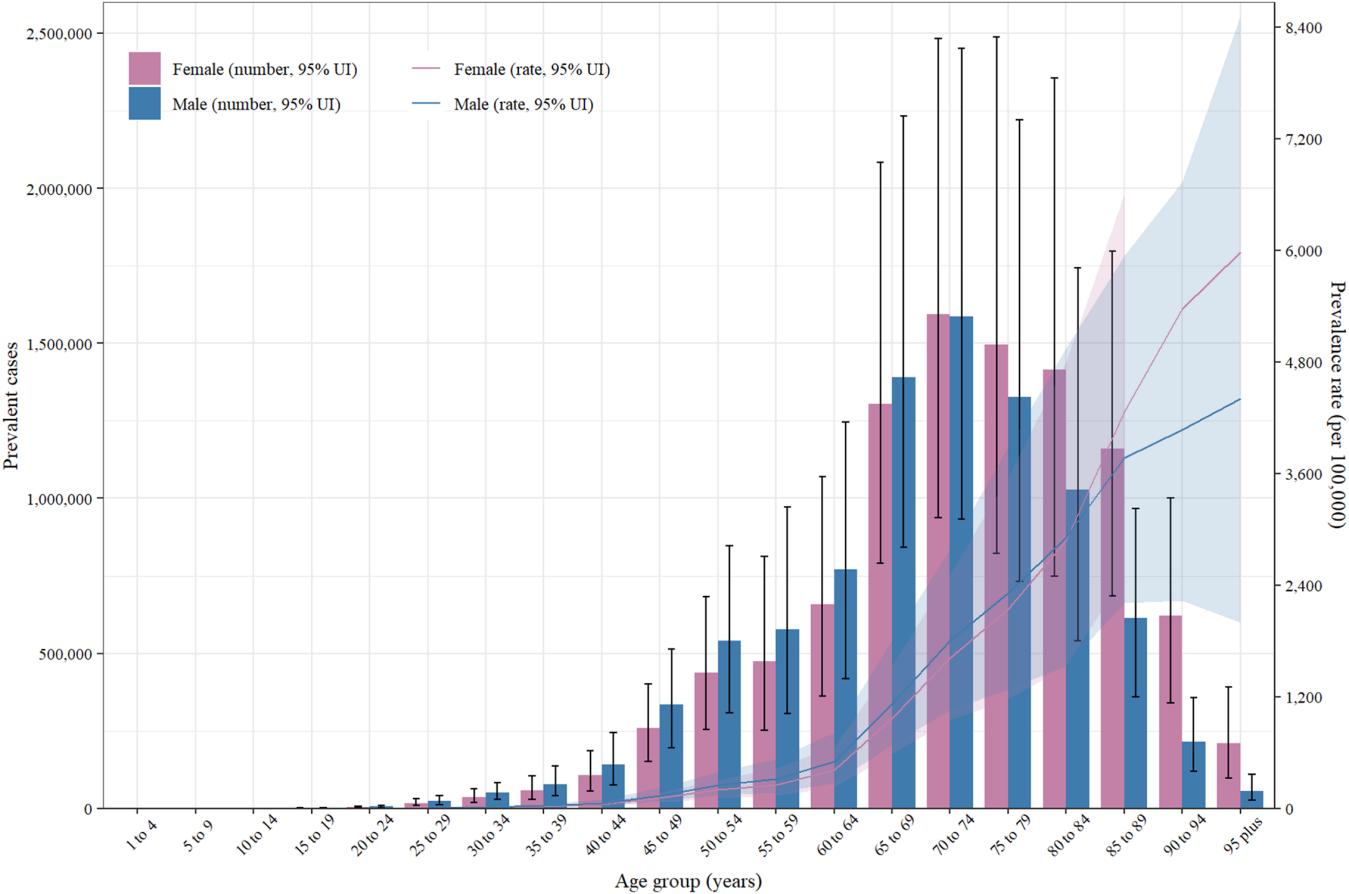
Number of combined cases and incidence of hypertension and heart disease per 100,000 people worldwide, categorized by age and sex (2019). The lines represent common cases with 95% uncertainty intervals for both sexes.

Starting in 1990, the worldwide incidence of HHD increased among individuals aged 35–39 years, reaching its zenith in the most senior age group (≥95 years) and escalating with advancing age in both sexes. Similarly, the highest incidence was observed in the 70–74-year age group, which subsequently decreased as age increased. HHD is more common in men aged 60–89 years and in women aged 60–94 years (Figure 3). In 2019, there was a global peak in deaths due to HHD among the oldest age group (≥95 years), with no significant difference observed between men and women across all age groups. Mortality rates peaked in the 80–84-year age group for both sexes and subsequently decreased as age increased. (Supplementary Figure S2). Among men, the general DALY rate for HHD increased with advancing age, while in women, this rate increased among participants up to the 85–89-year age group. Furthermore, the highest number of DALYs was observed in the 70–74-year age group, with a greater number noted in women between 80 and 84 years of age (Supplementary Figure S3).

### Connections to sociodemographic markers

**Figure 4:**
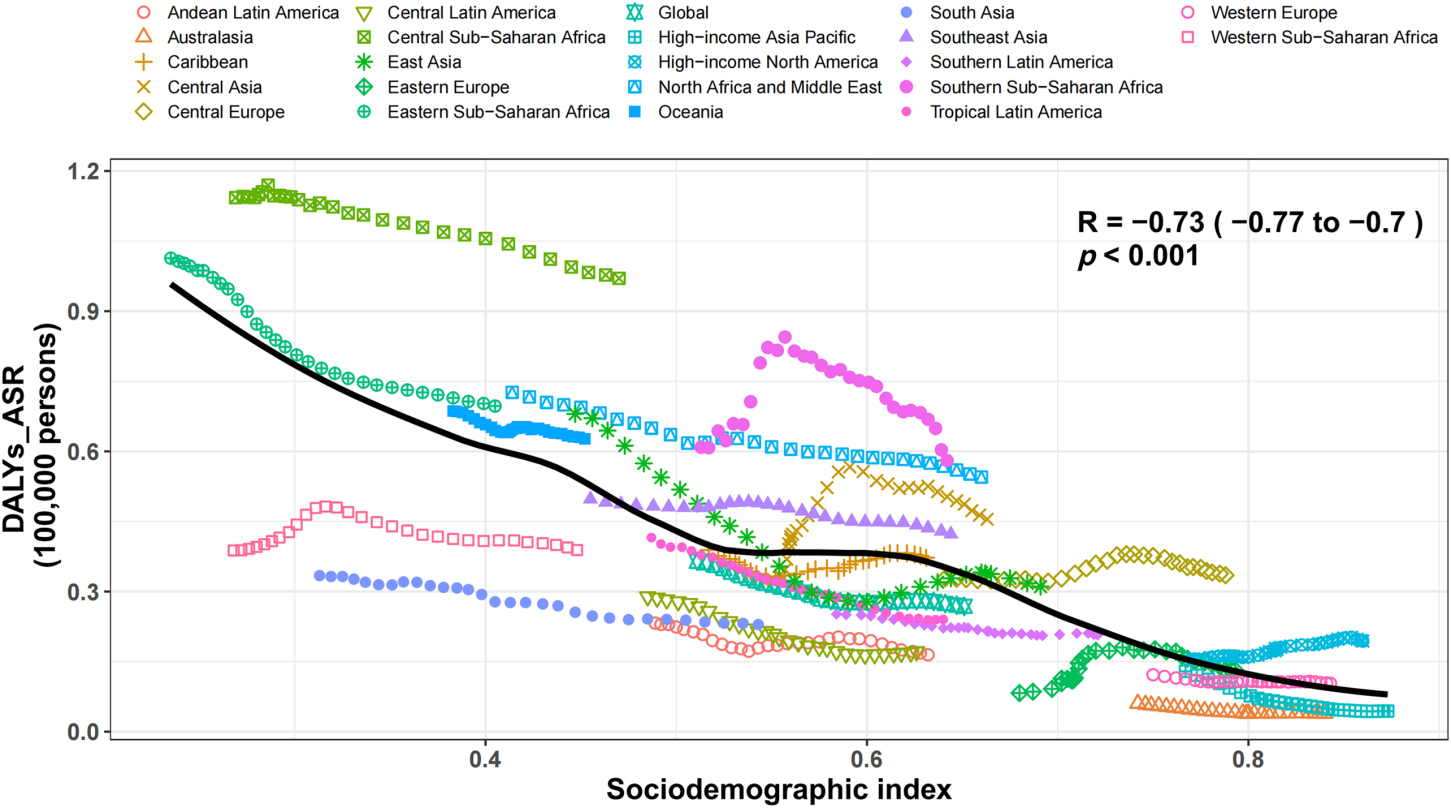
Age-standardized disability-adjusted life years (DALYs) statistics for hypertensive heart disease across 21 regions included in the GBD. Thirty data points per region depict the age-standardized DALYs rates observed from 1990 to 2019 in each region. The anticipated figures, which are derived from the sociodemographic index and disease frequencies across all sites, are depicted as a continuous line. DALYs rate lines that are plotted above the solid line indicate regions with a greater burden than what was anticipated (for instance, Central sub-Saharan Africa), while DALYs rate lines that are plotted below the line indicate regions with a burden that was below what was anticipated (such as South Asia). DALYs, disability-adjusted life-years; ASRs, age-standardized rates.

The cross-region analysis revealed an inverse relationship between the sociodemographic measure of HHD and the age-adjusted DALY rates from 1990 to 2019, as evidenced by an R value of −0.73 (*P* < 0.001). With the increase in sociodemographic indices, there was a rapid decrease in the age-adjusted DALY rates. From 1990 to 2019, the DALY figures in Central sub-Saharan Africa, sub-Saharan Africa, North Africa, and the Middle East exceeded predictions based on their sociodemographic information. The DALY rates in western sub-Saharan Africa, South Asia, and Western Europe were below what was expected (Figure 4). Nationally, there was a downward trend in the prevalence of HHD that correlated with increasing socioeconomic status in 2019, with an R value of −0.51 (*P* < 0.001) (Supplementary Figure S4). Nations and areas such as Afghanistan, Cook Islands, and the Central African Republic faced greater challenges than anticipated, in contrast to Niger, Nepal, and Guatemala, which experienced significantly lower burdens than predicted (Supplementary Figure S4).

### Risk factors

**Figure 5:**
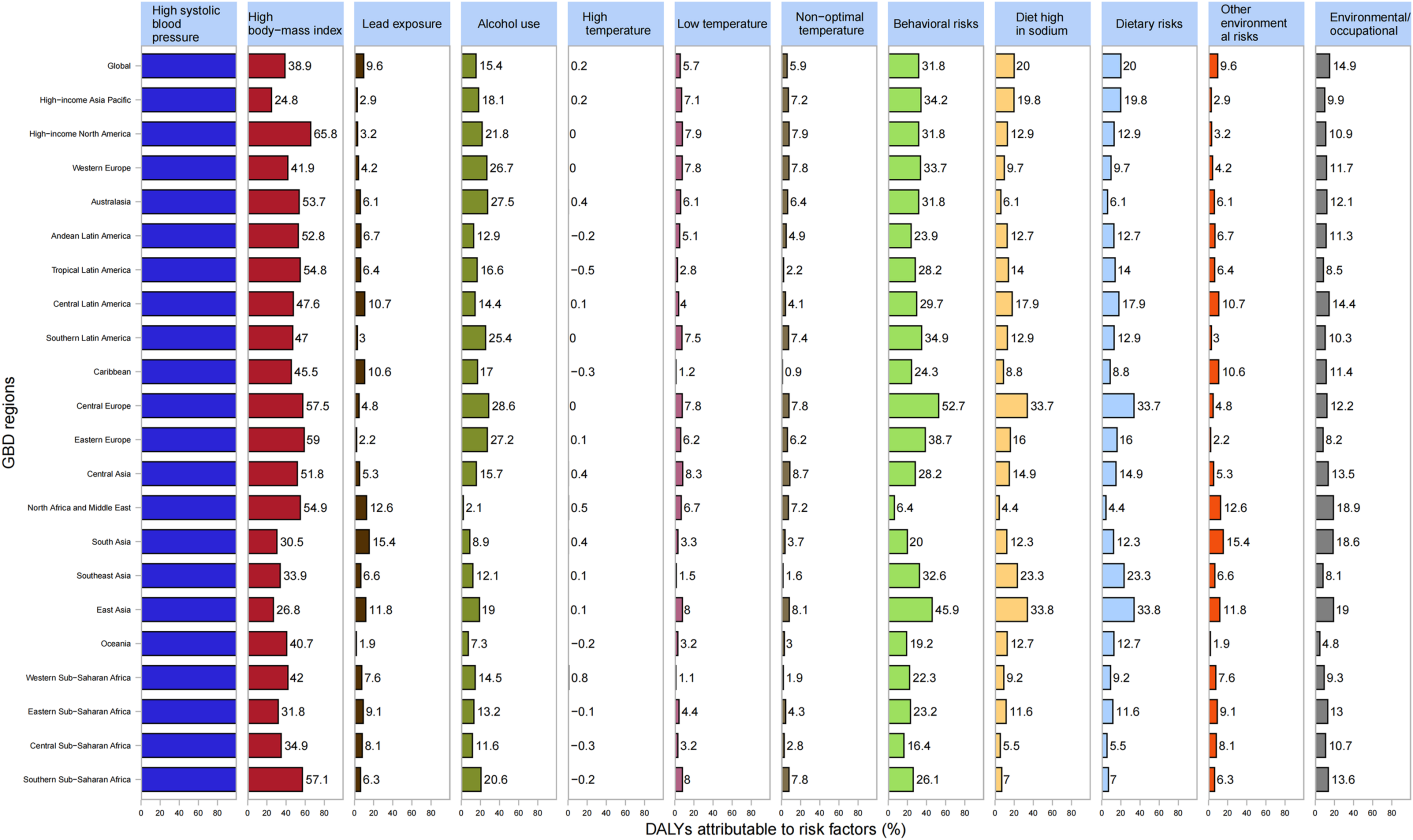
Proportion of disability-adjusted life years (DALYs) resulting from hypertensive heart disease linked to each risk factor associated with male sex across the 21 GBD regions in 2019. DALYs, disability-adjusted life-years; GBD, global burden of disease study.

The percentage of DALYs caused by HHD were linked to specific risk elements varied throughout the Global Burden of Disease regions. Worldwide, elevated systolic blood pressure (100%), increased body mass index (38.9%), and heightened behavioral risk (31.8%) were the primary factors in men's DALYs caused by HHD (Figure 5). Similarly, in women, high systolic blood pressure (100%), high body mass index (42.0%), and behavioral risk (16.5%) were markedly associated with HHD-related DALYs (Supplementary Figure S5).

### Predictions from the APC model

**Figure 6:**
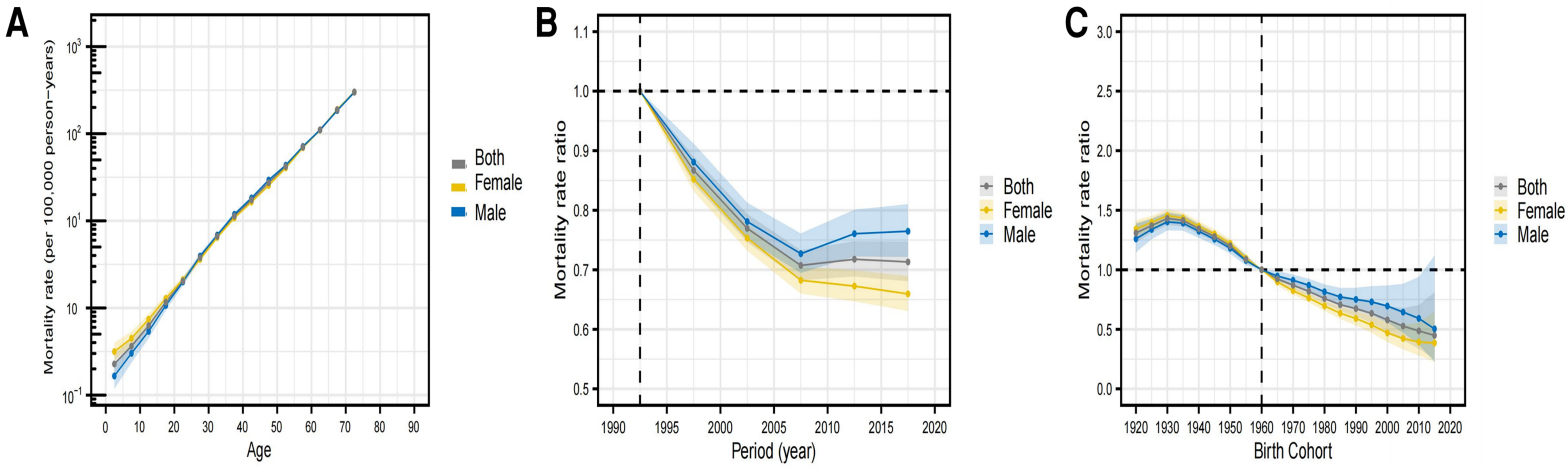
Influence of age, time frame, and group on global HHD death rates. (**A**) The influence of age is illustrated via modified longitudinal mortality graphs (per 100,000 person-years), considering variations across periods. (**B**) The influence of various timeframes is illustrated via the relative mortality risk (mortality rate ratio) and determined by the age-related rate ratio from 1990 to 1994 (the standard mortality rate ratio). The effect is evident in the relative mortality risk (ratio of mortality rates) and is determined by contrasting age-linked rates between 1990 and 1994 (the benchmark period) and 2015–2019. (**C**) The influence of cohort is illustrated by the relative mortality risk, which was determined by contrasting the age-related statistics of the 1920 and 2015 cohorts. The cohort data spanned from 1920 to 2015, and 1960 was used as the benchmark cohort. Dotted and shaded areas symbolize mortality rates and rate ratios, respectively, accompanied by their respective 95% uncertainty intervals. HHD stands for hypertensive heart disease.

In Figure 6, global estimates of HHD derived from the APC model are displayed for the APC effect. This includes the age effect, depicted as a longitudinal curve illustrating the progression of age-related deaths due to HHD; the period effect, shown as the comparative risk of death per period, aiding in monitoring the progression over time; and the cohort effect, indicated by the comparative risk of death per cohort, serving to observe mortality variations among different birth cohorts. There were variations in death rates among the different birth cohorts. Panel A indicates a steady age-related increase in deaths among HHD patients worldwide. Panel B indicates a decrease in mortality risk over time, decelerating after 2007. Cohort impacts, quantified as the comparative risk of death per group, serve to monitor mortality shifts among birth groups. The trends for both sexes combined and males almost coincided, whereas for females, the trends markedly decreased, indicating a lower mortality risk ratio for females over time. Panel C depicts a reduction in the mortality risk ratio with birth year, suggesting better survival when using 1960 as the baseline group.

## Discussion

### Main findings

Using data from the 2019 GBD Study, this study provides an up-to-date understanding of the frequency, death rate, and DALYs associated with HHD between 1990 and 2019, incorporating age-adjusted figures from 204 countries and regions. Globally, in 2019, HHD accounted for 18.6 million typical cases, 1.16 million deaths, and 21.51 million DALYs. Although there has been a reduction in mortality and DALYs due to HHD over the past three decades, there has been an increase in age-adjusted prevalence rates. Studies have shown that a global increase in DALYs associated with HHD is connected to demographic growth and aging (16, 17). These results might arise from population growth and aging as well as longer life expectancies. HHD, classified as an aging-related disease, intensifies the burden of both individual and global diseases (18). Clinically, these findings emphasize the importance of early detection and management of HTN to prevent the progression to HHD. The global increase in DALYs associated with HHD signifies a substantial burden on healthcare systems, particularly in low- and middle-income countries (LMICs) where resources are limited. The disparity in HHD burden between high-income countries (HICs) and LMICs highlights the need for improved healthcare infrastructure and accessibility in LMICs to address chronic diseases effectively.

The results of our study suggest a slight link between the Incidence of HHD and mortality rates in regional assessments. For example, East Asia (426.1), Oceania (344.9), and Southeast Asia (334.8) had the highest age-adjusted rates of HHD per 100,000 individuals, unlike sub-Saharan Central Africa (53.5), sub-Saharan Eastern Africa (38.8), and sub-Saharan Africa (34.1), which had the highest age-adjusted mortality rates due to HHD. This inequality becomes more apparent in poorer countries, where medical systems are customized for sudden illnesses such as infectious diseases and are deficient in chronic disease management (19). Significant regional differences in HHD prevalence and mortality can be attributed to three things: first, differences in healthcare infrastructure lead to different outcomes in hypertension management and HHD treatment (19); second, economic factors influence health behaviors and healthcare accessibility, with individuals living in low-income regions being more susceptible to poor diets and lifestyles (20); and finally, differences in public health policies and health awareness affect the effectiveness of disease prevention and management (1). Together, these factors contribute to significant differences in the burden of HHD in different regions of the globe.

Nationally, there was a significant fluctuation in the percentage of age-adjusted prevalence rates between 1990 and 2019, with the most substantial increases observed in Bolivia (Plurinational State of) (103.3%), Italy (42.9%), and Cameroon (40.6%). Concurrently, Estonia (695.5%), Latvia (515.7%), and the Republic of Moldova (485.9%) experienced the most significant increases in age-adjusted mortality rates. From 1990 to 2019, Estonia (473.0%), Latvia (420.1%), and the Republic of Moldova (340.0%) experienced the largest surge in age-standardized DALY rates for HHD. The World Health Organization predicted that from 2015 to 2050, the percentage of individuals aged 60 and above will increase twofold, from 12% to 22% (21). Consequently, the effective prevention and ideal management of HHD warrant increased focus from senior patients, governmental bodies, and medical professionals, particularly in nations with population aging such as Japan, China, and Italy.

Consistent with those of earlier research (22), our findings indicate a modest reduction in age-adjusted prevalence, mortality, and disability-adjusted life-year rates among men, mainly due to variances in BMI and behavioral risk. Nonetheless, the literature indicates (22) a greater propensity for women to suffer from HHD compared to men, attributed to elevated BMI. The increased incidence of HTN and obesity in women (23, 24) might explain these results. Regarding the distribution of ages, we observed a significant correlation between the age-adjusted impact of HHD and advancing age, which was consistent with earlier findings. Given that HHD is recognized as a disease linked to aging, its worldwide impact escalates with the aging of both patients and the entire population (25, 26). Prevention efforts should be focused on managing these risk factors through lifestyle interventions, such as promoting healthy diets, increasing physical activity, and implementing public health policies aimed at reducing the prevalence of HTN and obesity. Additionally, early screening and treatment of HTN can significantly reduce the burden of HHD, as indicated by Drazner (6), who emphasized the importance of early intervention in preventing the progression of HHD.

Negative correlations were observed between DALYs from HHD and sociodemographic measures at both the national and regional levels (R = −0.51 (*P* < 0.001) and R = −0.73 (*P* < 0.001), respectively). These correlations were further corroborated by related research (1) that revealed a negative correlation between the prevalence of HHD and the developmental stage of various regions and countries. Our study also revealed that HHD remains a significant public health issue in nations with low sociodemographic indices, as evidenced by the high age-standardized DALY rates in regions like Central sub-Saharan Africa. This finding is corroborated by Musaige (27), who reported high prevalence and increasing trends of obesity—a significant risk factor for HHD—in the Eastern Mediterranean region, contributing to the higher burden of HHD in these areas.

While HHD is inherently exclusive to HTN patients (with an assignable risk of 100%), a minority of HTN patients experience severe HHD. Other factors also contribute to the prevalence of HHD. Worldwide, figures from 2019 indicate that an increased risk of HHD is associated with a higher BMI. The majority of research on obesity predominantly uses BMI as the key indicator of obesity. Research indicates a surge in obesity rates within countries with electronic health records (27). Lifestyle shifts, encompassing diets rich in fats (notably saturated fats), cholesterol, and processed carbohydrates yet deficient in polyunsaturated fats and fiber, inactivity, and stress are the likely causes of the increased obesity prevalence among the regions (27). Elevated systolic blood pressure (SBP) is a direct indicator of hypertension and a major driver of HHD. High SBP increases the workload of the heart, leading to left ventricular hypertrophy, a hallmark of HHD, which may eventually lead to heart failure (2). Our analysis of the 2019 Global Burden of Disease data suggests that elevated SBP is associated with a significant increase in the prevalence and mortality of HHD. In regions with the highest levels of SBP, such as East Asia and Eastern Europe, the burden of HHD is correspondingly higher (1). Specifically, age-adjusted HHD mortality due to elevated SBP in these regions is significantly higher than the global average, indicating a strong correlation between high SBP and adverse HHD outcomes. High body mass index (BMI), a marker of overweight and obesity, indirectly contributes to HHD by exacerbating hypertension and triggering metabolic abnormalities (3). Obesity-related inflammation and insulin resistance further exacerbate the stress on the cardiovascular system, enhancing the impact of elevated SBP. In our study, we found that a high BMI is particularly significant in regions with high obesity rates such as North America and the Middle East. The data suggest that a high BMI is a significant contributor to HHD-associated DALYs, particularly among young adults who have not yet developed elevated SBP but already experience cardiovascular stress associated with a high BMI. Women account for a greater proportion of HHD DALYs due to elevated BMI, reflecting sex differences in fat distribution and metabolic responses (28).

Projections of the worldwide cohort impact of HHD on age, based on the APC model, indicate a steady rise in death rates as patients with HHD age worldwide. Over time, there was a decrease in the mortality risk ratio, which decreased after 2007. During this period, the mortality risk ratio was marginally reduced in women. With 1960 as the benchmark group, there was a decrease in mortality risk ratios as the birth year advanced, signifying enhanced survival rates, as corroborated by associated research (29).

HHD includes a range of organ-related effects, including left ventricular hypertrophy (LVH) and malfunctions in both systolic and diastolic functions (30). The incidence of LVH varies depending on these factors. The frequency of LVH varies according to detection techniques. Electrocardiographic findings show LVH in 3% of males and 1.5% of females, yet this figure increases to 15%–20% when assessing both sexes combined. The lack of advanced techniques such as echocardiography and magnetic resonance imaging in low-SDI regions might lead to an underestimation of HHD incidence in these regions. Advocating for increased public awareness of HHD risks and access to advanced testing methods in less developed areas is essential (31).

HHD is characterized by a range of irregularities, encompassing changes in LV structure, enlargement of the LV, and both systolic and diastolic malfunctions (2). Nonetheless, HHD is classified as heart failure, with symptoms stemming from the immediate and chronic influence of HTN (8). Considering the anticipated increase in life span and HTN prevalence in the next ten years (6, 21), HHD might represent a more important target in the prevention of cardiovascular diseases (2).

Furthermore, significant contextual factors linked to patient traits might also play a role in the subpar performance of countries in terms of burden. Given that HHD advances as a heart condition, characterized by an extended natural progression and a significantly increased likelihood of earlier mortality, prompt identification and treatment are crucial for improving patient prognosis and preventing early death (6). Pertinent to the execution of preventive and screening initiatives, those with lower socioeconomic status and health literacy tend to participate less in healthcare-seeking actions (20), resulting in the underrecognition of HTN and, consequently, ineffective management of HTN. Furthermore, lifestyle choices such as excessive sodium consumption, overweight status, and lack of physical activity might play a role in the heightened prevalence of HHD (32). Consequently, a variety of implementation approaches are essential to address the barriers at the patient, provider, and system levels necessary for enhancing HHD management in every nation.

### Strengths and limitations of this research

In this paper, we measured the burden of HHD in different regions and countries around the globe by analyzing HHD prevalence, mortality, and disability-adjusted life years (DALYs) between 1990 and 2019. We analyzed the major risk factors affecting HHD, including high SBP and high BMI, and assessed the importance of these factors by calculating the contribution of different risk factors to DALYs. The APC model was used to analyze age, period, and cohort effects of HHD. The model revealed increased mortality with age, decreased risk of death over time, and improved survival in later birth cohorts. However, this research has several limitations. First, a limited number of top-tier epidemiological databases exist for assessing the impact of HHD. Second, while uncommon, certain risk factors, such as elevated temperatures, were overlooked in our calculations. Third, the reliability of statistics is largely reliant on the volume and caliber of accessible national data. In certain regions with low to medium SDIs, such as Africa and Latin America, there is a notable or extreme sparsity of data. The majority of data regarding the disease burden in sub-Saharan Africa are obtained through extrapolation (33). Fourth, the absence of efficient death registration systems in numerous nations necessitates the use of oral autopsy examinations to approximate the death toll. This implies the possibility of underestimating the evidence. Enhancing vital registration systems could lead to more precise data and more effective disease management. Such constraints underscore the need for enhanced precision, better data gathering, and the adoption of more cohesive case definitions, facilitating more accurate international comparisons.

## Conclusions

HHD represents a significant public health issue, entailing substantial health care and financial expenses. Despite a decrease in point prevalence, mortality, and DALY rates throughout the duration of the study, there has been a corresponding increase in incidence. As the population ages, the issue of HHD will persist as a more significant concern. The documented worldwide, regional, and national impacts of HHD, its risk factors, and the effects of APC modeling could aid in more precisely predicting future disease burdens. Such insights could help decision-makers devise management strategies and service provisions to address the escalating medical requirements linked to HHD and its concurrent conditions.

## Data Availability

The original contributions presented in the study are included in the article/**Supplementary Material**, further inquiries can be directed to the corresponding author.
