# Supplementary material for: Impact of hypertensive heart disease, risk factors, and age-period-cohort models across 204 nations and regions from 1990 to 2019: a global perspective from the 2019 global burden of disease study
Source: Front Cardiovasc Med. 2024 Jul 18;11:1417523. doi: 10.3389/fcvm.2024.1417523 (PMC11291211; doi:10.3389/fcvm.2024.1417523)
Supplement: Supplementary file 1 [file Datasheet1.docx]

**Table S1**: Prevalent cases of hypertensive heart disease in 1990 and 2019 and the percentage change in the age-standardized rates (ASRs) per 100,000 people by location

|  | **Table S1: Prevalent cases of hypertensive heart disease in 1990 and 2019 and the percentage change in the age-standardized rates (ASRs) per 100,000, by location** | | | | | |  |
| --- | --- | --- | --- | --- | --- | --- | --- |
|  | Location | 1990 |  | 2019 |  | Percentage change in the  ASRs per 100,000 |  |
|  |  | No.（95% UI） | ASRs per 100,000 (95% UI) | No.（95% UI） | ASRs per 100,000 (95% UI) |  |  |
| 1 | Global | 7817320 (5616194,10643040) | 219.5 (158.8,299.4) | 18598025 (13544365,24898412) | 233.8 (170.5,312.9) | 6.5 (3.6,9.8) |  |
| 2 | Andean Latin America | 21047 (14079,30859) | 118.6 (79.4,174.2) | 74404 (51478,105633) | 138.4 (95.1,196.9) | 16.7 (7.6,28.9) |  |
| 3 | Bolivia (Plurinational State of) | 1154 (398,2434) | 52.5 (21,102) | 7814 (4484,12450) | 106.8 (63.9,163.8) | 103.3 (47,250.2) |  |
| 4 | Ecuador | 9303 (6282,13570) | 201.1 (136.8,288.6) | 31763 (21938,44873) | 224 (154.7,315.6) | 11.4 (0.2,27.7) |  |
| 5 | Peru | 10589 (7282,15290) | 100.2 (67.6,145.1) | 34827 (24495,48582) | 109.7 (76.7,154.3) | 9.5 (-1.4,23) |  |
| 6 | Australasia | 26073 (18754,35736) | 115.3 (83.8,156.4) | 42554 (30483,57290) | 79.1 (57.2,106.2) | -31.4 (-37, -25.7) |  |
| 7 | Australia | 22388 (16087,30680) | 119.3 (86.6,161) | 36332 (25672,49417) | 80.1 (57.4,108.1) | -32.8 (-38.9, -26.3) |  |
| 8 | New Zealand | 3685 (2478,5427) | 96.2 (65.9,140.3) | 6222 (4338,8630) | 73.8 (51.6,100.8) | -23.3 (-34.2, -9.2) |  |
| 9 | Caribbean | 51476 (37403,70941) | 208.4 (152,285.1) | 104765 (76579,144495) | 202.5 (147.9,279.7) | -2.8 (-6.5,1.5) |  |
| 10 | Antigua and Barbuda | 160 (120,204) | 282.3 (215.8,356.3) | 265 (192,360) | 283 (208.1,385.4) | 0.2 (-16.7,18.9) |  |
| 11 | Bahamas | 463 (344,620) | 336.7 (248.4,447.8) | 1143 (849,1508) | 332 (245.9,444.5) | -1.4 (-10.4,7.4) |  |
| 12 | Barbados | 714 (506,1044) | 225.3 (163.3,316.9) | 1116 (799,1554) | 224.2 (162.1,310.3) | -0.5 (-9.1,8.7) |  |
| 13 | Belize | 231 (168,323) | 259.6 (187.9,363.2) | 639 (461,885) | 263.7 (187.4,369.6) | 1.6 (-8.1,11.5) |  |
| 14 | Bermuda | 83 (59,114) | 139.4 (99.5,192.7) | 173 (126,251) | 128.4 (93.4,183.8) | -7.9 (-16,1.9) |  |
| 15 | Cuba | 16238 (11684,23134) | 159.5 (116.7,222.6) | 28346 (20626,39606) | 144.2 (105.1,200.8) | -9.6 (-18.1, -1.4) |  |
| 16 | Dominica | 206 (147,290) | 271 (198.1,370.7) | 240 (173,326) | 263.5 (190.5,356.5) | -2.8 (-12,6.2) |  |
| 17 | Dominican Republic | 7441 (5346,10309) | 227.1 (164.2,314.6) | 20690 (14882,29199) | 236.9 (168.8,336.5) | 4.4 (-4.9,15) |  |
| 18 | Grenada | 174 (122,244) | 220.6 (157.8,305.1) | 226 (162,314) | 221.3 (159.1,306) | 0.3 (-9.1,10.1) |  |
| 19 | Guyana | 921 (669,1267) | 288.5 (209.6,394.9) | 1444 (1043,1953) | 277.1 (198.2,377.2) | -3.9 (-12.8,5.4) |  |
| 20 | Haiti | 4822 (3333,6742) | 186.3 (133.6,256.5) | 11203 (7924,15494) | 201.3 (142.5,281.7) | 8.1 (-2.8,19.7) |  |
| 21 | Jamaica | 5613 (4052,7422) | 304.6 (222.3,398.4) | 9787 (7305,12720) | 317.8 (234.4,416.8) | 4.3 (-4.4,13) |  |
| 22 | Puerto Rico | 9355 (6639,13251) | 258.5 (187.3,360) | 18568 (13107,26681) | 239.4 (169.9,335.1) | -7.4 (-15.8,2.3) |  |
| 23 | Saint Kitts and Nevis | 85 (59,125) | 219.4 (157,307.5) | 119 (85,163) | 216 (153,305.2) | -1.5 (-10.8,8.9) |  |
| 24 | Saint Lucia | 219 (155,307) | 258.5 (188.7,354.5) | 531 (381,737) | 256.2 (184.7,355.5) | -0.9 (-9.2,9.2) |  |
| 25 | Saint Vincent and the Grenadines | 206 (147,286) | 291.3 (212.2,396) | 373 (267,509) | 290.5 (209.4,395.6) | -0.3 (-9.2,10.5) |  |
| 26 | Suriname | 607 (441,826) | 256.3 (184.2,353.7) | 1472 (1079,2017) | 265.3 (194.2,365.6) | 3.5 (-6.5,14) |  |
| 27 | Trinidad and Tobago | 2064 (1464,2926) | 263 (188,366.3) | 4515 (3217,6355) | 252.8 (182,354) | -3.9 (-12.6,5.3) |  |
| 28 | United States Virgin Islands | 159 (115,219) | 214.2 (153.8,295.8) | 367 (252,531) | 197.7 (139.9,278.4) | -7.7 (-16.2,0.8) |  |
| 29 | Central Asia | 86571 (62031,117014) | 209.4 (150.7,285.6) | 116888 (83382,159101) | 190.2 (133.2,262.4) | -9.2 (-14.9, -4.3) |  |
| 30 | Armenia | 2856 (2010,4092) | 129.6 (90.5,185.5) | 4889 (3313,6946) | 121.3 (82,172.4) | -6.4 (-15.3,4.5) |  |
| 31 | Azerbaijan | 7737 (5398,11022) | 180.4 (124.1,261.3) | 11983 (8128,17306) | 165.7 (113,242.6) | -8.2 (-17.5,2.3) |  |
| 32 | Georgia | 14328 (9810,20744) | 258.6 (178.6,366.2) | 14082 (9289,20537) | 220.6 (145.1,323.7) | -14.7 (-27, -4.1) |  |
| 33 | Kazakhstan | 14369 (9771,20591) | 128.6 (88.1,183.9) | 17372 (11744,25472) | 114.5 (77,165.5) | -11 (-21, -0.5) |  |
| 34 | Kyrgyzstan | 2916 (2013,4162) | 103.2 (71.5,148.6) | 3726 (2594,5321) | 95.9 (65.9,137.7) | -7.1 (-15.2,2.4) |  |
| 35 | Mongolia | 957 (656,1391) | 103.4 (72,149.2) | 1860 (1263,2657) | 103.3 (71,150.7) | -0.2 (-10.8,11.4) |  |
| 36 | Tajikistan | 6174 (4402,8347) | 235.1 (166.1,324.1) | 8361 (5932,11566) | 225.6 (159.4,316.1) | -4 (-13.7,6.9) |  |
| 37 | Turkmenistan | 3762 (2547,5208) | 223.3 (151.1,315.7) | 7363 (5122,10292) | 220.6 (150.3,315.8) | -1.2 (-11.6,9.8) |  |
| 38 | Uzbekistan | 33473 (24854,45016) | 329.3 (241,444) | 47255 (33333,63935) | 320.6 (230.2,430) | -2.6 (-12.8,7.6) |  |
| 39 | Central Europe | 272435 (187078,391798) | 197.3 (138.5,280.2) | 392401 (275256,555968) | 173.8 (123.5,242) | -11.9 (-19.6, -2.9) |  |
| 40 | Albania | 2024 (1385,2871) | 112.7 (76.4,160.7) | 4012 (2670,5900) | 92.5 (62,133.5) | -17.9 (-26.3, -8.8) |  |
| 41 | Bosnia and Herzegovina | 4767 (3288,6786) | 143 (98.6,203.2) | 6530 (4310,9551) | 110.4 (74.3,158.4) | -22.8 (-32.1, -13.2) |  |
| 42 | Bulgaria | 37996 (25153,54891) | 333.1 (231,466.8) | 41941 (27889,60467) | 270.1 (185.4,379) | -18.9 (-28.4, -9.2) |  |
| 43 | Croatia | 10812 (7074,15942) | 181.6 (121.3,262.7) | 10812 (7362,15532) | 112 (77.6,158.7) | -38.3 (-52.1, -20.5) |  |
| 44 | Czechia | 15625 (10946,21853) | 117 (82.9,161.2) | 26793 (18223,37633) | 120.9 (84.1,167.1) | 3.4 (-14.3,26.6) |  |
| 45 | Hungary | 36175 (24143,53359) | 251.2 (169.9,363.7) | 34882 (21594,53968) | 168.2 (106.6,254.3) | -33.1 (-43.7, -24.2) |  |
| 46 | Montenegro | 431 (297,630) | 75.1 (51.8,109.3) | 562 (375,831) | 57.5 (39.2,82.9) | -23.5 (-32.2, -14) |  |
| 47 | North Macedonia | 3580 (2423,5126) | 226.5 (155.3,324.9) | 5337 (3534,8071) | 181.4 (123.4,269.1) | -19.9 (-29, -10.7) |  |
| 48 | Poland | 77534 (52859,113546) | 188.5 (130.8,272.4) | 143489 (99972,203600) | 196.8 (137.5,278.7) | 4.4 (-7.7,18.6) |  |
| 49 | Romania | 56836 (37064,83509) | 222.3 (148.6,317.2) | 80847 (52111,121241) | 200.3 (129.5,297.5) | -9.9 (-25.6,7.1) |  |
| 50 | Serbia | 12386 (8242,17855) | 125.4 (82.5,182.1) | 12380 (7542,18885) | 74.8 (46.3,111.5) | -40.3 (-55.9, -23.8) |  |
| 51 | Slovakia | 9351 (6442,13555) | 160.4 (112.1,227.4) | 15634 (11311,21197) | 167.6 (122.2,225.1) | 4.5 (-15.4,32.6) |  |
| 52 | Slovenia | 4918 (3348,7338) | 206.1 (142.2,299.9) | 9181 (6658,12524) | 189.2 (138.1,254.1) | -8.2 (-24.9,16.1) |  |
| 53 | Central Latin America | 113216 (79226,160502) | 159.9 (112.3,227.4) | 343583 (244587,482451) | 153.5 (109,216.1) | -4 (-7.2, -0.5) |  |
| 54 | Colombia | 27784 (19501,39415) | 187.4 (131.9,265.6) | 93733 (67132,129804) | 175.1 (124.7,245.8) | -6.5 (-14.8,3.3) |  |
| 55 | Costa Rica | 2818 (1961,3991) | 175.1 (123.1,245.9) | 8280 (5890,11462) | 164.7 (116.5,228.9) | -5.9 (-15.4,3.9) |  |
| 56 | El Salvador | 2855 (1942,4116) | 104.3 (71.1,151.2) | 7060 (4962,9887) | 114.6 (80,161.4) | 9.9 (-1.8,22.6) |  |
| 57 | Guatemala | 2121 (1355,3194) | 77.7 (51.7,115.4) | 9954 (6773,14415) | 96.6 (67.3,139.4) | 24.3 (8.9,42.5) |  |
| 58 | Honduras | 2263 (1483,3336) | 129.1 (84.1,188.3) | 7653 (5235,11010) | 143.1 (97.7,204.7) | 10.9 (-0.1,25.3) |  |
| 59 | Mexico | 54830 (37137,79620) | 150.3 (103.9,218.1) | 151459 (104719,217552) | 139.5 (96.5,200.9) | -7.2 (-9.4, -4.6) |  |
| 60 | Nicaragua | 2101 (1436,3042) | 162.4 (110.4,235.4) | 6734 (4775,9646) | 175.5 (122.7,248.8) | 8.1 (-2,19.7) |  |
| 61 | Panama | 1847 (1291,2631) | 133.3 (92.2,189.2) | 5560 (3981,7741) | 134 (94.9,188) | 0.5 (-9.3,10.4) |  |
| 62 | Venezuela (Bolivarian Republic of) | 16597 (11052,23889) | 205.9 (139.3,292.7) | 53148 (36024,77082) | 198.1 (134.1,286.9) | -3.8 (-13.5,6.5) |  |
| 63 | Central Sub-Saharan Africa | 28245 (18161,43435) | 150 (97.3,224.1) | 66013 (43795,99015) | 147.6 (95.6,222.5) | -1.6 (-8.6,5.5) |  |
| 64 | Angola | 4564 (2980,6932) | 143.8 (93.8,215) | 13918 (9145,20880) | 150.7 (98.3,225.9) | 4.8 (-5.3,15.8) |  |
| 65 | Central African Republic | 1228 (776,1896) | 132.3 (85.5,200.2) | 2087 (1312,3131) | 124.4 (80.2,187.9) | -5.9 (-16.3,4.3) |  |
| 66 | Congo | 1409 (888,2186) | 156.4 (100.9,234.5) | 3456 (2224,5168) | 157.5 (100.4,237.3) | 0.7 (-8.9,11.5) |  |
| 67 | Democratic Republic of the Congo | 20010 (12718,30962) | 152.2 (97.6,227.3) | 44440 (29395,66679) | 146.9 (95.4,221.9) | -3.5 (-12.1,5.9) |  |
| 68 | Equatorial Guinea | 230 (148,356) | 137.4 (89.4,206.6) | 637 (421,949) | 153 (98.6,233.6) | 11.3 (-0.1,24.3) |  |
| 69 | Gabon | 804 (515,1238) | 158.8 (102,235.7) | 1475 (964,2196) | 159.8 (103.4,239.1) | 0.6 (-8.1,10.1) |  |
| 70 | East Asia | 3042447 (2152424,4183548) | 452.9 (326.2,616.5) | 8080427 (5821121,11009520) | 426.1 (306.6,574.8) | -5.9 (-8.6, -2.8) |  |
| 71 | China | 2974458 (2099431,4091288) | 461 (331.7,627.5) | 7903479 (5679083,10769048) | 433.5 (311.6,583) | -6 (-8.7, -2.9) |  |
| 72 | Democratic People's Republic of Korea | 28164 (20221,38939) | 230.8 (165.9,320.1) | 64456 (46179,90741) | 220 (156.2,308.7) | -4.7 (-13.7,6.6) |  |
| 73 | Taiwan (Province of China) | 39826 (28438,56010) | 305.1 (217.7,415.3) | 112492 (83574,150700) | 281.8 (209.4,374.7) | -7.7 (-21.1,7.9) |  |
| 74 | Eastern Europe | 146907 (97884,218307) | 56.1 (38.3,81.9) | 198892 (133630,295134) | 56.5 (38.2,83) | 0.7 (-2.5,3.4) |  |
| 75 | Belarus | 3703 (2551,5344) | 29.5 (20.5,41.9) | 4519 (3140,6394) | 28.4 (19.7,39.7) | -3.8 (-13.4,6.6) |  |
| 76 | Estonia | 4208 (2835,6243) | 208.4 (142.5,307.6) | 5596 (3674,8329) | 189.6 (126.9,279.9) | -9 (-20,3.2) |  |
| 77 | Latvia | 2471 (1677,3698) | 69.2 (46.9,102) | 2927 (1947,4457) | 64.7 (43.4,95.1) | -6.6 (-18.3,6.5) |  |
| 78 | Lithuania | 2873 (1980,4170) | 64.2 (44.4,92.3) | 3510 (2329,5244) | 56.8 (38.9,83) | -11.5 (-21.3, -0.6) |  |
| 79 | Republic of Moldova | 3816 (2569,5552) | 98.8 (68.1,142.1) | 5589 (3789,8177) | 97 (66,140.8) | -1.8 (-12.7,10.3) |  |
| 80 | Russian Federation | 121633 (79972,182261) | 73.1 (49.2,107.9) | 167583 (112254,251814) | 70.5 (47.4,105.1) | -3.4 (-6.6, -0.7) |  |
| 81 | Ukraine | 8202 (5375,12051) | 12.3 (8.2,17.9) | 9169 (6002,13718) | 11.9 (8,17.3) | -3.6 (-12.5,4.9) |  |
| 82 | Eastern Sub-Saharan Africa | 170962 (117520,234458) | 269.4 (184.8,372.8) | 394198 (272501,537479) | 281.8 (193.7,387.8) | 4.6 (1.6,7.6) |  |
| 83 | Burundi | 5514 (3611,7912) | 265.7 (177.4,378.8) | 10627 (7196,14820) | 280.6 (187.4,401.4) | 5.6 (-3.6,15.4) |  |
| 84 | Comoros | 616 (412,880) | 301.5 (202.7,425.1) | 1383 (928,1971) | 305.1 (204,433.9) | 1.2 (-8.3,11.6) |  |
| 85 | Djibouti | 342 (236,479) | 299.9 (200.9,428.8) | 1600 (1085,2234) | 319.3 (213.6,459.3) | 6.5 (-2.3,16.6) |  |
| 86 | Eritrea | 1696 (1144,2365) | 219.5 (149.5,314.3) | 5275 (3569,7276) | 242.3 (162.6,343.8) | 10.4 (0.4,21.8) |  |
| 87 | Ethiopia | 38262 (26383,51882) | 235 (162.6,316.3) | 99067 (69761,132517) | 273.6 (188.4,367.9) | 16.4 (10,22.8) |  |
| 88 | Kenya | 23386 (16526,30806) | 328.1 (229.5,431.7) | 57898 (41074,76843) | 310.5 (217.1,420.5) | -5.4 (-9.5, -0.7) |  |
| 89 | Madagascar | 12613 (8603,17572) | 280.4 (188.7,397.2) | 25847 (17596,35898) | 287 (192.4,412.5) | 2.4 (-6.2,11.6) |  |
| 90 | Malawi | 9715 (6476,13683) | 296.3 (200.9,419.7) | 18481 (12230,26216) | 289.1 (190.9,417.2) | -2.4 (-10.7,7) |  |
| 91 | Mozambique | 12241 (7912,17964) | 237.5 (155.6,357.7) | 23974 (15697,34849) | 254.1 (165.2,372.8) | 7 (-3.5,17.3) |  |
| 92 | Rwanda | 5916 (3964,8456) | 236.6 (157.1,340.5) | 12974 (8745,18401) | 249.5 (166.8,357.2) | 5.5 (-3.6,16.9) |  |
| 93 | Somalia | 5001 (3393,6908) | 249.6 (167.4,356.8) | 13545 (8992,19178) | 248.7 (167.5,359.6) | -0.4 (-9.8,9.5) |  |
| 94 | South Sudan | 6022 (4043,8534) | 279 (186.5,396.9) | 9582 (6587,13231) | 287.1 (193.8,403.9) | 2.9 (-6.3,12.8) |  |
| 95 | Uganda | 15979 (10732,22302) | 282.2 (191.8,399.9) | 33275 (23096,45675) | 274.4 (189.5,380.7) | -2.8 (-11.6,6.9) |  |
| 96 | United Republic of Tanzania | 27028 (18058,37992) | 283.2 (187.5,395.9) | 64852 (44525,90586) | 299.2 (201.9,421.5) | 5.6 (-3.9,15.3) |  |
| 97 | Zambia | 6507 (4359,9331) | 271.9 (180.2,393.2) | 15505 (10442,21769) | 275.4 (183.4,392.7) | 1.3 (-7.9,11) |  |
| 98 | High-income Asia Pacific | 166816 (114305,231899) | 94.2 (65.1,131.6) | 434593 (304460,608287) | 85.1 (61.5,114.6) | -9.7 (-21.8,5) |  |
| 99 | Brunei Darussalam | 82 (57,113) | 109.3 (75.3,154.9) | 194 (130,274) | 85.8 (56.6,123.9) | -21.5 (-31.8, -11.5) |  |
| 100 | Japan | 136188 (91930,193251) | 89.8 (61.2,126.9) | 332382 (228419,475399) | 78.4 (55.7,106.8) | -12.7 (-26.9,5.5) |  |
| 101 | Republic of Korea | 27758 (19666,38138) | 120.7 (85.4,167.1) | 93991 (65770,128763) | 111.6 (78,153) | -7.5 (-16.5,3) |  |
| 102 | Singapore | 2788 (2035,3749) | 140.6 (103.3,191.3) | 8025 (5821,11030) | 106.5 (76.7,146.2) | -24.3 (-30.5, -17.4) |  |
| 103 | High-income North America | 843289 (603037,1130929) | 245.5 (178.2,325.2) | 1540544 (1125394,2037713) | 253.1 (188.8,330.6) | 3.1 (-9.7,18) |  |
| 104 | Canada | 39449 (29357,51716) | 123.4 (93.1,159.8) | 52350 (38961,67755) | 78.9 (59.2,102.8) | -36 (-41.9, -29.4) |  |
| 105 | Greenland | 62 (45,83) | 195.6 (142.9,264) | 82 (59,111) | 133.4 (97.9,178.6) | -31.8 (-38.2, -25.2) |  |
| 106 | United States of America | 803759 (577215,1075049) | 258.3 (187,343.2) | 1488088 (1084320,1971720) | 274.3 (203.7,359.4) | 6.2 (-7.6,22.1) |  |
| 107 | North Africa and Middle East | 505025 (359681,689938) | 325 (237.4,443.5) | 1331826 (975253,1764467) | 333.9 (249.1,446.5) | 2.7 (-2.2,8.3) |  |
| 108 | Afghanistan | 21291 (14997,29443) | 333.3 (243,456.3) | 39579 (28818,53681) | 357.6 (261.6,491.3) | 7.3 (-2.9,19.1) |  |
| 109 | Algeria | 42105 (29624,58365) | 371.8 (270.1,508.7) | 119773 (86458,165674) | 374.7 (270.4,512.6) | 0.8 (-7.2,9.8) |  |
| 110 | Bahrain | 281 (204,383) | 158.4 (115.7,218.3) | 1516 (1062,2116) | 145.3 (105.2,197.9) | -8.3 (-15.6, -0.3) |  |
| 111 | Egypt | 71381 (45914,105337) | 270.7 (177.2,399.7) | 162516 (104367,243761) | 283.1 (183.8,416.5) | 4.6 (-5.3,14.5) |  |
| 112 | Iran (Islamic Republic of) | 98881 (70664,135953) | 420.1 (303,566.2) | 285078 (206673,380471) | 406.3 (293.7,546.3) | -3.3 (-5.3, -1.3) |  |
| 113 | Iraq | 18187 (13154,25048) | 252.5 (184.7,351.6) | 51927 (37016,71798) | 249.8 (179.6,349.7) | -1.1 (-9.9,8.3) |  |
| 114 | Jordan | 6439 (4678,8510) | 536.7 (395.1,710.9) | 33818 (25037,44682) | 561.6 (417.9,747.6) | 4.6 (-4.3,15.4) |  |
| 115 | Kuwait | 2917 (2152,3806) | 512.2 (373.8,682.8) | 13041 (9528,17029) | 514.9 (376.3,690.6) | 0.5 (-7.8,9.8) |  |
| 116 | Lebanon | 8688 (6252,11916) | 410.5 (300.1,556.5) | 22311 (16115,30920) | 428.8 (312,592.5) | 4.5 (-3.1,14.1) |  |
| 117 | Libya | 6482 (4757,8646) | 370.8 (272.5,501.2) | 18883 (13850,25346) | 389.5 (282.7,534.1) | 5.1 (-4.3,15) |  |
| 118 | Morocco | 44309 (31772,60228) | 353 (255.5,483.2) | 103513 (74316,142740) | 355.9 (258.7,487.7) | 0.8 (-8.1,10.3) |  |
| 119 | Oman | 1214 (926,1565) | 183.7 (141.5,241.6) | 3960 (2837,5270) | 223.1 (162.4,303.1) | 21.4 (6.1,40.1) |  |
| 120 | Palestine | 2713 (1977,3736) | 336.9 (245.9,464.8) | 7271 (5212,9904) | 337 (243.8,466.3) | 0 (-9.3,9.9) |  |
| 121 | Qatar | 178 (136,232) | 116.6 (89.2,152) | 1276 (882,1791) | 105.2 (76.2,143.1) | -9.7 (-22.7,5.8) |  |
| 122 | Saudi Arabia | 4967 (3559,6699) | 87.7 (63.4,119.7) | 17027 (11901,23339) | 94.9 (69.5,129) | 8.1 (-1.1,19.4) |  |
| 123 | Sudan | 28345 (20275,39612) | 330.6 (239.6,456.9) | 62867 (45361,85548) | 364.6 (263.6,500) | 10.3 (0.2,21.7) |  |
| 124 | Syrian Arab Republic | 5092 (3646,6977) | 106.1 (76.3,145.1) | 12862 (9140,17925) | 112.3 (81.1,156.4) | 5.8 (-2.5,15.4) |  |
| 125 | Tunisia | 17496 (12285,23865) | 371.8 (267.3,501.6) | 46301 (33668,62461) | 378.2 (277.5,513.6) | 1.7 (-7.4,11.3) |  |
| 126 | Turkey | 106555 (78652,144873) | 327.7 (242.1,450.8) | 263261 (201351,340627) | 313.1 (236.5,406.3) | -4.5 (-20.6,16.9) |  |
| 127 | United Arab Emirates | 1874 (1351,2542) | 403.1 (292.2,547.1) | 18434 (12770,25845) | 424 (305.2,577.3) | 5.2 (-4.5,17.3) |  |
| 128 | Yemen | 15290 (11027,21274) | 351.7 (258.8,479.7) | 45257 (32491,61533) | 375.9 (274.8,511.6) | 6.9 (-3.3,18.7) |  |
| 129 | Oceania | 7765 (5478,10545) | 341 (241.6,471.3) | 18807 (13405,25671) | 344.9 (248.5,477.9) | 1.1 (-4.8,7.8) |  |
| 130 | American Samoa | 51 (36,70) | 276.1 (197.9,384.5) | 116 (83,161) | 274 (198.2,376.7) | -0.7 (-10,8.2) |  |
| 131 | Cook Islands | 79 (60,101) | 717.4 (544.6,928.2) | 170 (128,226) | 703.1 (532.9,920.7) | -2 (-9.9,6) |  |
| 132 | Fiji | 1073 (762,1460) | 388.5 (277.3,539.1) | 2296 (1637,3142) | 383.6 (275.9,529.3) | -1.2 (-10.5,8.2) |  |
| 133 | Guam | 265 (190,361) | 428.3 (309.3,599) | 816 (588,1113) | 443.9 (320.4,622.7) | 3.6 (-5.3,12.9) |  |
| 134 | Kiribati | 64 (44,89) | 229.3 (163,326.7) | 118 (82,164) | 238.6 (169,336.9) | 4.1 (-6,14.6) |  |
| 135 | Marshall Islands | 47 (33,66) | 351.6 (250.3,490) | 90 (64,124) | 357.8 (256.7,502.2) | 1.8 (-7.7,11.9) |  |
| 136 | Micronesia (Federated States of) | 137 (97,192) | 364.8 (265,510.9) | 190 (136,256) | 363.3 (261.3,497.9) | -0.4 (-9.8,9.9) |  |
| 137 | Nauru | 8 (6,12) | 331.3 (233.1,468.9) | 8 (6,11) | 328.9 (236,464.5) | -0.7 (-9.9,9.2) |  |
| 138 | Niue | 9 (6,12) | 381.1 (273,530.2) | 8 (6,11) | 382.4 (276.5,536.4) | 0.3 (-8.3,10) |  |
| 139 | Northern Mariana Islands | 25 (18,34) | 201.7 (145.9,284.9) | 86 (60,121) | 208.6 (148.2,292.7) | 3.4 (-5.3,12.6) |  |
| 140 | Palau | 7 (5,9) | 80.3 (57.9,111.4) | 14 (10,19) | 79.4 (57,110.9) | -1.2 (-10.1,9) |  |
| 141 | Papua New Guinea | 4673 (3240,6409) | 332 (232.7,462) | 12178 (8598,16516) | 339.7 (243.4,475.7) | 2.3 (-7.1,13.3) |  |
| 142 | Samoa | 309 (219,425) | 407.2 (292.8,564.3) | 514 (374,705) | 398.8 (287.5,554.5) | -2 (-9.9,7.1) |  |
| 143 | Solomon Islands | 252 (175,352) | 264.8 (187.9,375.7) | 589 (411,818) | 267.6 (188.9,379.9) | 1 (-9.1,12.2) |  |
| 144 | Tokelau | 5 (4,7) | 381.4 (273.9,537.3) | 5 (4,7) | 398.5 (291.5,548.7) | 4.5 (-4.7,15.9) |  |
| 145 | Tonga | 126 (89,173) | 257.3 (184.8,356.9) | 195 (140,271) | 255 (181.9,355.7) | -0.9 (-8.9,8) |  |
| 146 | Tuvalu | 20 (14,28) | 352.6 (253.2,491.4) | 33 (23,46) | 365.3 (263,508.3) | 3.6 (-5.9,13.5) |  |
| 147 | Vanuatu | 185 (128,255) | 347.2 (247.2,482.8) | 491 (340,690) | 336.9 (242.7,473) | -3 (-11.5,6.7) |  |
| 148 | South Asia | 432561 (302776,619930) | 110.2 (77.4,156.8) | 1305144 (917051,1870774) | 111.8 (78.3,159) | 1.5 (-0.6,3.5) |  |
| 149 | Bangladesh | 44065 (31391,62584) | 117.4 (84,164.6) | 144543 (101781,203094) | 126.4 (90,175.2) | 7.7 (-2.7,19.2) |  |
| 150 | Bhutan | 150 (106,211) | 86.7 (62.2,121.7) | 438 (309,621) | 90.1 (63.5,125.8) | 3.9 (-6.2,14.9) |  |
| 151 | India | 319066 (220334,462725) | 104 (72.2,148.7) | 1035567 (719986,1488984) | 108.6 (75.5,155.2) | 4.4 (2.4,6.4) |  |
| 152 | Nepal | 4723 (3383,6724) | 69.4 (48.9,97.9) | 12933 (9146,18475) | 69.9 (49.5,98.3) | 0.6 (-9.1,12.1) |  |
| 153 | Pakistan | 64558 (44674,91613) | 133.5 (92.6,188.5) | 111664 (78337,159376) | 138.6 (96.2,196.9) | 3.8 (-2.3,9.8) |  |
| 154 | Southeast Asia | 737433 (527507,986793) | 335 (244.4,456.5) | 1847718 (1330748,2473049) | 334.8 (244.8,451.6) | -0.1 (-3.3,3.4) |  |
| 155 | Cambodia | 11666 (8151,16045) | 312.2 (222.2,430.1) | 34107 (24089,47079) | 328 (231.9,452.5) | 5.1 (-4.7,15.4) |  |
| 156 | Indonesia | 344152 (244749,462954) | 423.2 (305.1,573.1) | 837613 (596609,1138432) | 451.9 (322.9,611) | 6.8 (3.4,10.3) |  |
| 157 | Lao People's Democratic Republic | 4684 (3211,6575) | 264.4 (186.4,367.8) | 10554 (7488,14606) | 283.9 (203.3,396.6) | 7.4 (-3.1,20.3) |  |
| 158 | Malaysia | 7401 (5404,10063) | 90.3 (65.5,123.1) | 23044 (16564,31400) | 95 (69.8,130.4) | 5.2 (-4.6,14.9) |  |
| 159 | Maldives | 105 (74,143) | 149.1 (107.1,205) | 419 (302,568) | 157.3 (112,219.2) | 5.6 (-4.7,15) |  |
| 160 | Mauritius | 2502 (1765,3450) | 390.1 (280.3,540.1) | 6127 (4429,8468) | 371.1 (270,519.5) | -4.9 (-13.5,5.5) |  |
| 161 | Myanmar | 62762 (44201,88057) | 316.6 (222.5,449.1) | 131884 (92534,180353) | 319.2 (226.3,449.4) | 0.8 (-9,11.8) |  |
| 162 | Philippines | 101261 (72534,134395) | 392.2 (283.8,523.2) | 284575 (203845,378810) | 415.9 (302,553.8) | 6 (4.5,7.6) |  |
| 163 | Seychelles | 263 (191,366) | 466.6 (339,643.4) | 467 (335,635) | 469 (334.3,649.3) | 0.5 (-8.2,10.9) |  |
| 164 | Sri Lanka | 29520 (20434,41073) | 313.1 (219.2,435.1) | 75612 (52302,105303) | 312.5 (220.9,435.7) | -0.2 (-9.5,10.3) |  |
| 165 | Thailand | 35127 (25097,48743) | 113.8 (81.4,158.8) | 117391 (83676,161305) | 117.2 (83.6,162.7) | 2.9 (-6.3,13) |  |
| 166 | Timor-Leste | 653 (472,889) | 295 (210.8,413.4) | 2382 (1619,3306) | 320.6 (224.6,445.6) | 8.7 (-2.2,19.7) |  |
| 167 | Viet Nam | 136356 (96869,187328) | 365.4 (261.5,511.9) | 321122 (240265,420453) | 381.2 (283.6,512.5) | 4.3 (-8.8,19.6) |  |
| 168 | Southern Latin America | 86517 (57959,127888) | 203.4 (136.8,295.5) | 158613 (108714,229191) | 184 (127,265.4) | -9.5 (-17.2, -1.1) |  |
| 169 | Uruguay | 8551 (5898,12390) | 218.2 (152.4,309.7) | 10213 (7132,14335) | 167.5 (118.3,233.6) | -23.2 (-30.3, -15.2) |  |
| 170 | Argentina | 52984 (34671,78778) | 177.3 (116.8,260.8) | 89467 (60856,132326) | 159.6 (109.1,235) | -10 (-20.7,2.2) |  |
| 171 | Chile | 24979 (16728,36249) | 288.6 (194.7,412.9) | 58926 (40264,83389) | 245.3 (167.6,346.7) | -15 (-24, -4.8) |  |
| 172 | Southern Sub-Saharan Africa | 39951 (26153,58731) | 162.8 (105.9,237.5) | 74240 (48396,110535) | 147.8 (95.8,216) | -9.2 (-13.1, -5.4) |  |
| 173 | Botswana | 445 (276,690) | 90.1 (57.6,135.9) | 1052 (671,1626) | 91.1 (58,140) | 1.2 (-7.8,9.4) |  |
| 174 | Eswatini | 217 (137,332) | 86.7 (54.9,130.8) | 425 (262,655) | 85.6 (54,130.4) | -1.2 (-11.3,8.6) |  |
| 175 | Lesotho | 684 (422,1063) | 77.7 (49,117.1) | 850 (528,1338) | 77.9 (49.4,120.8) | 0.3 (-9.2,10.7) |  |
| 176 | Namibia | 596 (365,948) | 90 (57.1,137.7) | 1136 (723,1729) | 88.7 (56.6,135.8) | -1.5 (-11.4,8.7) |  |
| 177 | South Africa | 35967 (23472,53331) | 190 (122.5,278.2) | 67334 (43834,100121) | 166.1 (108.1,241.9) | -12.6 (-16.4, -8.6) |  |
| 178 | Zimbabwe | 2042 (1420,2959) | 58.3 (41,84.5) | 3443 (2153,5308) | 57.9 (36.3,87.7) | -0.6 (-19.7,23.4) |  |
| 179 | Tropical Latin America | 128401 (87298,183773) | 173.8 (120.6,246.8) | 392810 (272306,557651) | 171 (118.4,242.8) | -1.6 (-5,2.4) |  |
| 180 | Brazil | 125539 (85484,180047) | 174.8 (120.9,248.5) | 385099 (265802,546990) | 171.5 (118.6,244) | -1.8 (-5.2,2.2) |  |
| 181 | Paraguay | 2862 (1984,4099) | 143.3 (99.1,206.5) | 7710 (5479,10879) | 149.8 (105.1,215.1) | 4.5 (-5.9,16.5) |  |
| 182 | Western Europe | 738823 (523897,1040758) | 123.5 (89.5,169.7) | 1298347 (923594,1798392) | 123.5 (89.2,169.2) | 0.1 (-6.4,7.6) |  |
| 183 | Andorra | 94 (64,136) | 198.7 (140.4,277.4) | 214 (150,302) | 146.1 (102.9,206.6) | -26.5 (-33, -19.5) |  |
| 184 | Austria | 34860 (25364,47396) | 276.2 (205.6,368.2) | 35307 (25854,47180) | 171.8 (127.2,227.3) | -37.8 (-43.6, -30.7) |  |
| 185 | Belgium | 14406 (9972,20657) | 90.6 (64.5,127.6) | 18412 (14034,24747) | 70.7 (53.9,93.7) | -22 (-36.1, -4.5) |  |
| 186 | Cyprus | 676 (456,1005) | 94.7 (65.2,134.7) | 1449 (1036,2021) | 73.6 (53.6,100.9) | -22.3 (-33.6, -6.8) |  |
| 187 | Denmark | 3892 (2840,5248) | 45.3 (33.7,60) | 3808 (2734,5262) | 30.6 (22.5,41.7) | -32.4 (-37.8, -26) |  |
| 188 | Finland | 8002 (5953,10521) | 112.7 (84.9,146.3) | 14946 (11058,19901) | 107 (80.7,140.4) | -5.1 (-15.6,6.5) |  |
| 189 | France | 110397 (77496,149251) | 125 (89.3,168.3) | 125428 (90023,171923) | 77 (55.6,105.1) | -38.4 (-44.7, -31.5) |  |
| 190 | Germany | 185294 (131943,254487) | 141.3 (102.3,192.2) | 344499 (241242,476410) | 160.3 (114.6,215.7) | 13.4 (1,27.9) |  |
| 191 | Greece | 19568 (13587,28205) | 128.7 (90.2,181.6) | 27913 (19532,39565) | 98 (70.3,138.9) | -23.9 (-30.4, -16.3) |  |
| 192 | Iceland | 256 (181,358) | 85.7 (60.9,119.2) | 420 (306,570) | 69.5 (50.9,94.7) | -18.9 (-29.1, -5.4) |  |
| 193 | Ireland | 2350 (1664,3322) | 57.6 (40.8,79.8) | 3106 (2176,4381) | 40.2 (28.5,56.4) | -30.3 (-36.6, -23.5) |  |
| 194 | Israel | 2666 (1878,3728) | 56.1 (40.4,76.7) | 5141 (3678,7089) | 41.9 (30,57.2) | -25.3 (-31.4, -18.7) |  |
| 195 | Italy | 169842 (105341,258520) | 190.8 (123.4,282.4) | 466722 (314561,664165) | 272.5 (188.1,386.7) | 42.9 (24.4,71.8) |  |
| 196 | Luxembourg | 666 (462,962) | 120.8 (85.3,170.1) | 1328 (956,1789) | 121.1 (88.3,161.2) | 0.2 (-17.3,23.6) |  |
| 197 | Malta | 497 (353,702) | 122.2 (86.8,170.8) | 1202 (848,1698) | 119.1 (84.8,166.1) | -2.6 (-15.1,14.9) |  |
| 198 | Monaco | 86 (59,123) | 105.7 (75,146.2) | 82 (56,115) | 74.6 (52.3,104.4) | -29.5 (-36, -22.5) |  |
| 199 | Netherlands | 9583 (7151,12681) | 46.7 (35.3,61.4) | 14018 (10007,19194) | 38.2 (27.4,51.9) | -18.2 (-31.9, -3.6) |  |
| 200 | Norway | 4572 (3007,6822) | 61.3 (41.5,89.6) | 6330 (4370,8936) | 60.3 (41.9,84.8) | -1.7 (-12.5,13) |  |
| 201 | Portugal | 15366 (10573,22080) | 113.7 (79.4,159.8) | 20703 (14657,28847) | 72.4 (51.7,99.5) | -36.3 (-45.8, -25.8) |  |
| 202 | San Marino | 50 (35,72) | 148.5 (105.8,209.3) | 78 (54,109) | 105.6 (75,151.4) | -28.9 (-35.2, -21.1) |  |
| 203 | Spain | 57392 (40979,80890) | 104.1 (74.7,142.2) | 81492 (56622,116366) | 72.1 (51.1,102.3) | -30.8 (-40.1, -20.8) |  |
| 204 | Sweden | 22522 (15244,32585) | 139.7 (96.6,196.1) | 37254 (25240,53976) | 150.1 (103.6,214) | 7.5 (-3.1,19.6) |  |
| 205 | Switzerland | 10896 (7628,15515) | 95.8 (68.5,133.5) | 16701 (12205,22768) | 82.3 (61.4,110.8) | -14.1 (-28.8,5.5) |  |
| 206 | United Kingdom | 64277 (43415,94818) | 68.1 (47.6,97.9) | 70663 (49498,99546) | 52.6 (37.6,72.5) | -22.8 (-30.1, -13.8) |  |
| 207 | Western Sub-Saharan Africa | 171363 (121204,233717) | 229.9 (161.2,310) | 381258 (267069,513647) | 242.9 (168.7,330.3) | 5.6 (2.2,8.9) |  |
| 208 | Benin | 3808 (2477,5648) | 211.3 (137.5,309.6) | 8991 (6005,12814) | 215.7 (142.1,313.4) | 2.1 (-6.2,11) |  |
| 209 | Burkina Faso | 7229 (4681,10789) | 198.2 (129.9,288.2) | 15174 (10057,21825) | 198.7 (131.1,290.2) | 0.3 (-8.3,10) |  |
| 210 | Cabo Verde | 605 (389,905) | 257.1 (168.3,377) | 1075 (717,1539) | 265.6 (173.4,390.2) | 3.3 (-5.5,12.8) |  |
| 211 | Cameroon | 4740 (3273,6999) | 134.5 (90.5,198.5) | 18546 (12245,26999) | 189.1 (122.6,281.5) | 40.6 (18.6,69.2) |  |
| 212 | Chad | 4874 (3135,7318) | 192.1 (125.1,284.9) | 9643 (6316,13975) | 204.5 (133,301.2) | 6.4 (-3.5,17.9) |  |
| 213 | C么te d'Ivoire | 6790 (4413,9876) | 223.3 (144.5,331.5) | 19059 (12651,27717) | 225.6 (145.1,336.2) | 1 (-7,9.7) |  |
| 214 | Gambia | 702 (457,1033) | 237.7 (153.8,351.3) | 2067 (1361,3004) | 244.8 (159,356.7) | 3 (-5.6,13.1) |  |
| 215 | Ghana | 5653 (3933,8121) | 112.1 (76.8,162.6) | 16691 (10762,25155) | 123.6 (78.5,185) | 10.3 (-8.7,34.1) |  |
| 216 | Guinea | 6538 (4616,9103) | 221.3 (156.6,304.8) | 11826 (8086,16606) | 243.1 (163.6,343.7) | 9.9 (-4.6,23.7) |  |
| 217 | Guinea-Bissau | 640 (409,948) | 195 (126.8,288.7) | 1188 (788,1735) | 208 (136.4,305.2) | 6.7 (-2.9,17.5) |  |
| 218 | Liberia | 2224 (1416,3322) | 225.3 (146.6,327) | 4145 (2776,5882) | 241.4 (156.4,352.6) | 7.2 (-2.1,17.7) |  |
| 219 | Mali | 6823 (4440,10185) | 198 (129.1,288.1) | 15460 (10189,22613) | 212.5 (138.4,311) | 7.3 (-2,17.6) |  |
| 220 | Mauritania | 2087 (1332,3085) | 233.5 (150.2,342.6) | 4676 (3101,6860) | 251.2 (165.4,367.3) | 7.6 (-2.2,18.1) |  |
| 221 | Niger | 4333 (2846,6302) | 193.2 (124.9,283.6) | 13117 (8562,19118) | 207.6 (135.9,300.5) | 7.5 (-2.8,18.2) |  |
| 222 | Nigeria | 100629 (71876,131042) | 264.8 (190.1,342.9) | 208484 (152033,268857) | 288 (205.8,375.3) | 8.7 (4.8,13.2) |  |
| 223 | Sao Tome and Principe | 134 (85,201) | 229.7 (149.7,336.8) | 223 (149,316) | 246.4 (163.7,358.9) | 7.3 (-1.8,17.1) |  |
| 224 | Senegal | 7204 (4806,10322) | 257.6 (170.7,367.9) | 16891 (11305,24140) | 257.9 (170,372) | 0.1 (-8.2,8.9) |  |
| 225 | Sierra Leone | 4034 (2613,5953) | 233.9 (151.1,343.4) | 7315 (4783,10643) | 237.7 (156.7,347.6) | 1.6 (-7.8,11.1) |  |
| 226 | Togo | 2311 (1534,3373) | 226.9 (148.5,337.3) | 6681 (4429,9697) | 226.6 (148.7,331.9) | -0.1 (-8.8,8.9) |  |

**Table S2**: Deaths due to hypertensive heart disease between 1990 and 2019 and the percentage change in the age-standardized rates (ASRs) per 100,000 by location

|  | **Table S2: Deaths due to hypertensive heart disease in 1990 and 2019 and the percentage change  in the age-standardized rates (ASRs) per 100,000 by location** | | | | | |
| --- | --- | --- | --- | --- | --- | --- |
|  | Location | 1990 |  | 2019 |  | Percentage  change in the ASRs per  100,000 |
|  |  | No. (95% UI) | ASRs per 100,000 (95% UI) | No. (95% UI) | ASRs per 100,000 (95% UI) |  |
| 1 | Global | 654906 (530566,732729) | 19.3 (15.9,21.6) | 1156733 (859826,1278563) | 15.2 (11.2,16.7) | -21.5 (-35.2, -10.1) |
| 2 | Andean Latin America | 2310 (1982,2638) | 13 (11.2,15) | 5165 (4245,6112) | 9.7 (8,11.5) | -25.2 (-40.8, -9.5) |
| 3 | Bolivia (Plurinational State of) | 552 (339,745) | 22.1 (14,30.1) | 1293 (904,1766) | 18.4 (12.8,24.8) | -16.6 (-42.8,16.6) |
| 4 | Ecuador | 1015 (910,1124) | 23.1 (20.5,25.6) | 2656 (2069,3276) | 21.4 (16.6,26.1) | -7.4 (-29.9,14.2) |
| 5 | Peru | 744 (620,885) | 7 (5.8,8.3) | 1216 (901,1650) | 3.7 (2.7,5) | -47.1 (-62.2, -25.6) |
| 6 | Australasia | 762 (583,827) | 3.6 (2.8,3.9) | 1478 (1115,1745) | 2.5 (2,3) | -29.2 (-37, -4.2) |
| 7 | Australia | 602 (459,659) | 3.4 (2.6,3.8) | 1243 (927,1475) | 2.5 (1.9,3) | -26.5 (-35.9, -2.9) |
| 8 | New Zealand | 160 (121,176) | 4.4 (3.4,4.8) | 235 (187,318) | 2.6 (2.1,3.6) | -39.4 (-47.9, -5.7) |
| 9 | Caribbean | 4616 (4018,5704) | 19.4 (16.8,23.8) | 9720 (8014,11466) | 18.7 (15.4,22.1) | -3.5 (-19.5,11) |
| 10 | Antigua and Barbuda | 17 (14,19) | 29.2 (24.2,32.8) | 27 (22,32) | 30.7 (24.3,36) | 5.2 (-11.3,24.7) |
| 11 | Bahamas | 75 (66,87) | 55.6 (48.8,64.6) | 194 (155,243) | 55.6 (44.5,69.3) | 0 (-18.4,23) |
| 12 | Barbados | 48 (40,54) | 15.8 (13.2,18) | 70 (55,86) | 14.3 (11.4,17.6) | -9.7 (-25.8,9.4) |
| 13 | Belize | 17 (14,20) | 18.8 (16.1,23) | 50 (40,59) | 20.3 (16.1,24) | 7.7 (-10.8,29.4) |
| 14 | Bermuda | 6 (4,6) | 10.1 (7.2,11.6) | 10 (7,13) | 7.3 (5.3,9.1) | -27.8 (-45.1, -6.4) |
| 15 | Cuba | 527 (471,791) | 5.4 (4.7,8.2) | 2299 (1150,2905) | 11.4 (5.8,14.4) | 112.7 (-16.4,180.2) |
| 16 | Dominica | 40 (35,46) | 54.7 (47.7,61.2) | 42 (34,52) | 46.3 (37.7,57.4) | -15.4 (-32.4,7.4) |
| 17 | Dominican Republic | 636 (548,734) | 20.8 (17.9,24.1) | 1891 (1411,2457) | 22.2 (16.6,28.7) | 6.4 (-22.4,42.3) |
| 18 | Grenada | 22 (19,26) | 27.9 (23.8,32.7) | 26 (21,30) | 27 (21.4,31) | -3.2 (-20.7,14.1) |
| 19 | Guyana | 266 (200,304) | 82.2 (65,93.5) | 308 (238,393) | 58.1 (45.8,73.7) | -29.3 (-46, -4.1) |
| 20 | Haiti | 1237 (810,2237) | 47.1 (30.4,94) | 2202 (1270,3469) | 39.2 (22.8,62) | -16.9 (-42.1,17.5) |
| 21 | Jamaica | 755 (446,843) | 41.3 (24.6,46) | 788 (620,1009) | 24.2 (19.1,31.2) | -41.5 (-54.9,5.5) |
| 22 | Puerto Rico | 375 (333,509) | 10.9 (9.6,14.8) | 914 (679,1159) | 11.3 (8.3,14.6) | 3.7 (-32,33.8) |
| 23 | Saint Kitts and Nevis | 11 (9,12) | 30.8 (25.3,35.3) | 14 (11,17) | 25.8 (20.5,31.3) | -16.3 (-34.3,4.4) |
| 24 | Saint Lucia | 26 (22,30) | 35 (28.4,39.5) | 48 (39,58) | 23.9 (19.4,29.1) | -31.8 (-43.9, -14.1) |
| 25 | Saint Vincent and the Grenadines | 26 (22,30) | 39.1 (33.4,45.7) | 45 (37,53) | 37.3 (30.7,43.4) | -4.6 (-18.5,11.5) |
| 26 | Suriname | 67 (57,74) | 27.8 (23.6,30.9) | 125 (98,152) | 22.5 (17.6,27.3) | -19.1 (-35.3,1.3) |
| 27 | Trinidad and Tobago | 293 (208,319) | 41.2 (29,44.8) | 294 (216,495) | 16.7 (12.3,28.1) | -59.4 (-70.9, -14.3) |
| 28 | United States Virgin Islands | 18 (15,23) | 26.3 (21.5,32.4) | 45 (38,53) | 26.8 (22.7,31.4) | 1.9 (-20.1,28.7) |
| 29 | Central Asia | 6746 (5925,9023) | 16.3 (14.2,21.9) | 14510 (12251,16492) | 27.8 (21.9,31.6) | 70.6 (16.9,110.6) |
| 30 | Armenia | 308 (142,401) | 15.6 (6.3,20.6) | 659 (266,809) | 17.3 (6.7,21.2) | 10.9 (-14.6,42.1) |
| 31 | Azerbaijan | 1247 (1011,1666) | 28.3 (22.8,38.1) | 1938 (1403,2642) | 32.6 (22.6,44.5) | 15.4 (-38.3,71.9) |
| 32 | Georgia | 769 (564,1600) | 15.1 (11,29.8) | 3862 (1585,4703) | 56.7 (24.1,68.8) | 275.9 (2.3,462) |
| 33 | Kazakhstan | 1490 (1221,2793) | 12.9 (10.4,25.1) | 2054 (1614,4272) | 13.9 (11,29.5) | 8.4 (-11.4,30.7) |
| 34 | Kyrgyzstan | 306 (260,396) | 10.6 (9.1,14.1) | 512 (393,610) | 13.2 (10.4,15.9) | 24.9 (-7.2,53.7) |
| 35 | Mongolia | 217 (123,266) | 23.9 (13.7,28.9) | 188 (143,243) | 11.2 (8.6,14) | -53.3 (-64.8, -25.3) |
| 36 | Tajikistan | 958 (634,1383) | 35.9 (23.9,52.3) | 1610 (1011,2012) | 54.2 (32.3,67.2) | 50.8 (5.9,101.5) |
| 37 | Turkmenistan | 221 (190,281) | 13.6 (11.7,17.9) | 527 (402,676) | 16.4 (12.3,20.8) | 20.4 (-12.1,56) |
| 38 | Uzbekistan | 1229 (990,1562) | 12 (9.6,15.4) | 3161 (2088,4285) | 25.9 (16.1,36.6) | 115.7 (26.9,249.5) |
| 39 | Central Europe | 25071 (23174,31235) | 19.1 (17.4,24.1) | 48702 (34931,56401) | 21.7 (15.5,25.1) | 13.4 (-26.2,30.5) |
| 40 | Albania | 185 (162,210) | 11.3 (9.8,12.8) | 272 (197,363) | 6.6 (4.8,8.7) | -41.9 (-57.7, -20.7) |
| 41 | Bosnia and Herzegovina | 356 (318,484) | 10.9 (9.5,16.4) | 526 (402,785) | 9.6 (7.5,14.3) | -11.9 (-30.7,9.3) |
| 42 | Bulgaria | 3223 (2812,5878) | 30.5 (26.4,58.3) | 11112 (6952,13891) | 75.1 (46.4,92.8) | 145.8 (-6.5,231.9) |
| 43 | Croatia | 1198 (797,1322) | 22.6 (14.6,24.9) | 1741 (1131,2164) | 18.6 (12,23.1) | -17.7 (-34.4,3.4) |
| 44 | Czechia | 451 (358,1210) | 3.4 (2.7,9.2) | 1570 (1114,1936) | 7.1 (5,8.8) | 108.4 (-39.8,203.2) |
| 45 | Hungary | 3579 (3216,4163) | 26.8 (24,31.5) | 5790 (3786,7074) | 27.9 (18.2,34.1) | 3.9 (-32.8,25.3) |
| 46 | Montenegro | 23 (19,27) | 4.1 (3.5,4.8) | 47 (37,59) | 5.3 (4.1,6.6) | 29.1 (-4.2,66.8) |
| 47 | North Macedonia | 630 (535,738) | 46.4 (39.1,55.7) | 994 (765,1203) | 44 (34.1,52.8) | -5.2 (-28.1,16.5) |
| 48 | Poland | 4525 (4103,6116) | 11.3 (10.1,15.7) | 8656 (6611,10502) | 11.6 (8.8,14.1) | 2.8 (-32.1,22.6) |
| 49 | Romania | 8118 (6728,8846) | 34.7 (29.8,38.3) | 12796 (9432,15433) | 31.5 (23.6,38) | -9.2 (-28.5,7.2) |
| 50 | Serbia | 2013 (1693,2535) | 22.7 (19.1,28.8) | 3170 (2340,3846) | 23.4 (17,28.1) | 2.8 (-27.7,30.7) |
| 51 | Slovakia | 383 (320,769) | 6.8 (5.7,13.9) | 1113 (786,1388) | 12.4 (8.6,15.3) | 82.2 (-32.9,150.2) |
| 52 | Slovenia | 388 (256,492) | 17 (11,21.7) | 915 (382,1206) | 16.7 (7.4,21.9) | -2.1 (-47,34.7) |
| 53 | Central Latin America | 11662 (8211,12414) | 17.3 (12.3,18.5) | 23559 (19425,29470) | 10.7 (8.8,13.3) | -38.4 (-47.9, -4.6) |
| 54 | Colombia | 3897 (2160,4303) | 27.8 (15.7,30.9) | 5465 (3949,8323) | 9.7 (7,14.9) | -65.2 (-74.4, -11.8) |
| 55 | Costa Rica | 184 (147,209) | 11.7 (9.2,13.3) | 562 (416,723) | 10.7 (8,13.8) | -8.8 (-28.2,14.1) |
| 56 | El Salvador | 212 (187,275) | 7.6 (6.6,9.9) | 384 (287,501) | 5.9 (4.4,7.7) | -22 (-42.6,1.9) |
| 57 | Guatemala | 279 (235,320) | 10.2 (8.8,12.6) | 664 (512,979) | 7.6 (5.9,11) | -25.4 (-41.6,1.8) |
| 58 | Honduras | 260 (205,434) | 14 (10.8,25.4) | 794 (517,1369) | 15.4 (10,28.2) | 10.2 (-28.3,48.8) |
| 59 | Mexico | 4054 (2880,4324) | 12.4 (8.6,13.3) | 9059 (7188,10606) | 8.8 (6.9,10.2) | -29.3 (-38.5, -11.5) |
| 60 | Nicaragua | 196 (168,219) | 15.5 (13,17.5) | 636 (510,739) | 18.9 (14.8,21.8) | 21.6 (-1.6,43.6) |
| 61 | Panama | 74 (63,108) | 5.3 (4.6,7.9) | 309 (229,398) | 7.2 (5.3,9.3) | 34.1 (-14.5,76.5) |
| 62 | Venezuela (Bolivarian Republic of) | 2504 (1950,2728) | 30.2 (24.2,33.1) | 5687 (4275,7879) | 21 (15.7,29) | -30.5 (-47.8,1.7) |
| 63 | Central Sub-Saharan Africa | 9959 (6348,13024) | 58.7 (38.6,78.1) | 20718 (13285,28061) | 53.5 (34.3,72.5) | -8.9 (-27,13.7) |
| 64 | Angola | 1671 (1049,2254) | 56.9 (36.8,80.7) | 3438 (2096,4622) | 44.6 (27,59.8) | -21.7 (-42.6,10.8) |
| 65 | Central African Republic | 647 (395,884) | 73.1 (46.1,102.7) | 1051 (619,1513) | 68.8 (40.9,104.8) | -5.9 (-28.8,26.6) |
| 66 | Congo | 610 (381,786) | 74.2 (47.3,98.2) | 1037 (666,1387) | 55.1 (36.2,73.7) | -25.8 (-44.2, -1.1) |
| 67 | Democratic Republic of the Congo | 6637 (4089,9089) | 56.6 (35.4,77.4) | 14648 (9104,20691) | 55.4 (34.5,78.4) | -2.1 (-25.3,27.7) |
| 68 | Equatorial Guinea | 107 (65,149) | 67.2 (40.9,104.1) | 139 (80,196) | 40 (24,54.7) | -40.4 (-64.6, -3.4) |
| 69 | Gabon | 288 (181,371) | 63.1 (40.1,80.6) | 405 (259,532) | 50.3 (32.4,65.9) | -20.4 (-39.9,6.4) |
| 70 | East Asia | 246300 (186574,280285) | 41.3 (32.9,46.6) | 330520 (211770,384586) | 20.3 (12.8,23.5) | -50.9 (-64.5, -40.3) |
| 71 | China | 241298 (180926,275423) | 42.2 (33.7,47.7) | 320090 (201652,373927) | 20.6 (12.7,23.9) | -51.2 (-65.3, -40.5) |
| 72 | Democratic People's Republic of Korea | 2508 (1675,3532) | 22.7 (15.2,32) | 6130 (4747,8064) | 22.3 (17.1,29.5) | -2 (-29.8,39.2) |
| 73 | Taiwan (Province of China) | 2495 (1615,2716) | 22.2 (15,24.3) | 4300 (3334,5843) | 10.5 (8.1,14.3) | -52.7 (-63.2, -14.8) |
| 74 | Eastern Europe | 9562 (8591,15629) | 3.7 (3.3,6.2) | 24642 (15124,28505) | 7.1 (4.4,8.2) | 90.8 (-10.5,125.8) |
| 75 | Belarus | 601 (414,753) | 4.8 (3.3,5.9) | 382 (275,713) | 2.4 (1.7,4.4) | -49.8 (-64.9,8.3) |
| 76 | Estonia | 155 (125,348) | 8 (6.4,17.7) | 1942 (414,2540) | 62.9 (14.5,81.9) | 690.5 (-10.8,1059.5) |
| 77 | Latvia | 82 (63,203) | 2.4 (1.9,5.9) | 662 (179,856) | 14.8 (4.1,19.1) | 515.7 (-14.8,831.8) |
| 78 | Lithuania | 151 (123,308) | 3.4 (2.8,7) | 509 (303,630) | 8.3 (4.9,10.3) | 143.6 (-15.2,233.8) |
| 79 | Republic of Moldova | 180 (146,408) | 4.7 (3.7,11) | 1591 (427,1974) | 27.3 (7.4,33.9) | 485.9 (-26,726.8) |
| 80 | Russian Federation | 7532 (6819,12451) | 4.6 (4.1,7.7) | 18411 (11834,21657) | 7.9 (5.1,9.2) | 72.1 (-16.1,107.7) |
| 81 | Ukraine | 861 (710,1433) | 1.4 (1.2,2.2) | 1144 (936,1755) | 1.6 (1.3,2.3) | 10.9 (-12.3,36.8) |
| 82 | Eastern Sub-Saharan Africa | 30158 (18332,43333) | 51.4 (31.6,75.4) | 47532 (27885,71756) | 38.8 (22.4,58.5) | -24.6 (-37.5, -4.7) |
| 83 | Burundi | 987 (160,1792) | 50.2 (7.9,92.4) | 1165 (481,2106) | 35.7 (14.4,64.3) | -29 (-51.3,112.7) |
| 84 | Comoros | 96 (51,146) | 51.9 (28.5,79.1) | 155 (89,239) | 37.6 (21.5,57.9) | -27.6 (-47.3,4.4) |
| 85 | Djibouti | 50 (30,73) | 52.1 (32.2,74.8) | 145 (83,223) | 36.9 (21.2,54.5) | -29.2 (-47.7, -0.7) |
| 86 | Eritrea | 474 (257,759) | 65.2 (34.9,119.8) | 992 (535,1608) | 55.5 (30.3,89.7) | -15 (-43.8,27.8) |
| 87 | Ethiopia | 8954 (4996,13491) | 58.7 (35,88.4) | 10237 (5713,17004) | 32 (17.8,53.1) | -45.5 (-60.8, -13.3) |
| 88 | Kenya | 2087 (1269,3220) | 32.2 (19.4,50.2) | 5075 (2991,8289) | 32.6 (18.8,54.3) | 1.3 (-15.4,22.2) |
| 89 | Madagascar | 3009 (1836,4217) | 68.6 (42.4,97) | 5304 (3083,7993) | 64.5 (38.4,98.7) | -6 (-29.2,24.7) |
| 90 | Malawi | 1274 (750,1870) | 44.4 (26.1,65.4) | 2043 (1147,3277) | 37.3 (20.7,60.1) | -16 (-36.2,17.2) |
| 91 | Mozambique | 2331 (1436,3460) | 52.9 (33.5,81) | 4072 (2417,6064) | 51.3 (30.6,77) | -3 (-28,33.2) |
| 92 | Rwanda | 1420 (416,2447) | 61.2 (17.6,114.8) | 1466 (421,2605) | 34.2 (9.7,60.7) | -44.2 (-60.5, -20.1) |
| 93 | Somalia | 1327 (756,1886) | 67.5 (41.9,96.1) | 2484 (1491,3744) | 54 (33.1,82.5) | -20 (-40.7,11.3) |
| 94 | South Sudan | 920 (510,1418) | 47.4 (27,73) | 915 (523,1606) | 31.9 (18.5,57.2) | -32.8 (-50.4, -5.7) |
| 95 | Uganda | 1855 (747,3166) | 36.7 (14.4,62.6) | 3470 (1229,5901) | 33 (11.5,55.6) | -9.9 (-30.6,15.6) |
| 96 | United Republic of Tanzania | 4185 (2526,6184) | 50.2 (29.9,73.9) | 7130 (3585,11487) | 37.3 (18.7,59.4) | -25.7 (-43.9, -3.5) |
| 97 | Zambia | 1166 (755,1513) | 53.7 (35.3,71.3) | 2841 (1735,3817) | 57.9 (35.9,77.5) | 7.8 (-21.1,46.8) |
| 98 | High-income Asia Pacific | 14459 (8287,15725) | 9 (4.9,9.9) | 19342 (14639,23661) | 3 (2.3,4) | -66.8 (-72.6, -27.5) |
| 99 | Brunei Darussalam | 16 (14,20) | 29.3 (24.2,36.1) | 29 (24,40) | 19.6 (16.3,26.3) | -33 (-45.7, -16.7) |
| 100 | Japan | 10832 (5133,11954) | 7.8 (3.5,8.7) | 13511 (9669,16433) | 2.4 (1.7,3.3) | -69.8 (-75.8, -12.3) |
| 101 | Republic of Korea | 3389 (2617,3743) | 17.6 (14.3,19.7) | 5086 (3947,6472) | 6.7 (5.2,8.4) | -61.8 (-70, -50.1) |
| 102 | Singapore | 222 (199,271) | 12 (10.6,16.1) | 715 (517,826) | 9.7 (7.2,11.2) | -19.4 (-52.1, -6.9) |
| 103 | High-income North America | 24339 (20615,26139) | 6.8 (5.8,7.3) | 54534 (36383,59533) | 8.3 (5.4,9) | 21.4 (-15.5,28) |
| 104 | Canada | 605 (465,662) | 2 (1.5,2.2) | 1566 (920,1836) | 2 (1.2,2.4) | 4.2 (-29.9,17.6) |
| 105 | Greenland | 3 (2,3) | 9.5 (7.8,11.6) | 4 (3,5) | 7.1 (5.7,8.9) | -24.4 (-43.1, -0.7) |
| 106 | United States of America | 23732 (20112,25466) | 7.3 (6.2,7.9) | 52963 (35453,57776) | 9 (5.9,9.8) | 23.7 (-13.8,30.6) |
| 107 | North Africa and Middle East | 53293 (32535,67378) | 41.1 (24.6,52.1) | 108862 (59840,135179) | 32.2 (17.1,39.3) | -21.7 (-41.8, -2.7) |
| 108 | Afghanistan | 4444 (1328,6730) | 74.5 (23.7,112.3) | 6845 (2245,10104) | 71.9 (24.3,104.5) | -3.5 (-28.1,30.5) |
| 109 | Algeria | 4431 (2026,6239) | 56.9 (26.1,78.2) | 8827 (3589,11895) | 36.7 (14.6,48.8) | -35.5 (-53, -8.5) |
| 110 | Bahrain | 14 (11,18) | 11.8 (9.6,17.6) | 37 (29,49) | 7.2 (5.7,10.6) | -38.6 (-55.5, -17.4) |
| 111 | Egypt | 11606 (5445,17608) | 53.8 (24.8,80.5) | 21692 (8571,37507) | 47.3 (17.6,80.2) | -12 (-45.5,19.7) |
| 112 | Iran (Islamic Republic of) | 6380 (5185,7919) | 37.2 (30.2,47.5) | 18477 (15571,20027) | 30.2 (25.1,32.8) | -19 (-41.9,1.3) |
| 113 | Iraq | 1503 (1073,1974) | 23.2 (16.3,30.6) | 3090 (2394,3706) | 18.7 (14.4,22.2) | -19.5 (-42.8,14.6) |
| 114 | Jordan | 540 (423,680) | 58.3 (45.3,74.8) | 1780 (1119,2140) | 39.9 (24.7,47.9) | -31.7 (-53.3, -11) |
| 115 | Kuwait | 146 (112,166) | 31.9 (24.7,36.7) | 394 (318,538) | 20.1 (16,26.2) | -36.8 (-49.5,0.5) |
| 116 | Lebanon | 599 (284,909) | 33.8 (16.2,50.8) | 1367 (546,1846) | 27.1 (10.9,36.6) | -19.7 (-58.5,28.3) |
| 117 | Libya | 374 (186,531) | 23.3 (11.8,33.1) | 1109 (461,1624) | 25.8 (10.7,38.1) | 10.4 (-27.7,61.2) |
| 118 | Morocco | 4574 (2196,6472) | 42.9 (20.7,64.4) | 9862 (4068,13500) | 40.5 (16.5,55.3) | -5.5 (-31.4,34.9) |
| 119 | Oman | 130 (93,181) | 29.2 (21.3,41.4) | 232 (171,282) | 23.6 (18.2,28.6) | -19.3 (-50.5,18.3) |
| 120 | Palestine | 238 (177,297) | 33.8 (25.2,41.9) | 602 (408,708) | 37.3 (24.7,43.9) | 10.5 (-26.7,52.1) |
| 121 | Qatar | 6 (5,9) | 10.8 (7.7,16.5) | 18 (13,26) | 5.8 (4.2,7.7) | -46.7 (-66.2, -17.3) |
| 122 | Saudi Arabia | 287 (205,456) | 5.7 (4.1,9.6) | 653 (485,838) | 4.3 (3.3,5.9) | -24.1 (-59.5,12.3) |
| 123 | Sudan | 4032 (1740,5667) | 53 (22.8,76) | 7087 (2787,10661) | 47.6 (18.4,70.6) | -10.2 (-35.2,27.4) |
| 124 | Syrian Arab Republic | 533 (387,735) | 12.6 (9.2,17.7) | 793 (591,1057) | 9.3 (7,12) | -26.8 (-52.8,10.3) |
| 125 | Tunisia | 1206 (606,1558) | 32.6 (16.5,42.3) | 2879 (1177,4083) | 26.6 (10.8,37.5) | -18.5 (-46.1,17.3) |
| 126 | Turkey | 9917 (6164,13881) | 35.3 (20.7,49.3) | 16257 (9127,20408) | 20.7 (11.3,26.1) | -41.2 (-61, -11.3) |
| 127 | United Arab Emirates | 122 (46,235) | 47.2 (17.5,85.2) | 1045 (349,2223) | 43.6 (15.4,85.5) | -7.7 (-47.9,65.1) |
| 128 | Yemen | 2176 (888,3123) | 59.4 (25,85) | 5705 (2259,9162) | 57 (22.2,93.6) | -4.1 (-35.3,38.6) |
| 129 | Oceania | 759 (519,1028) | 31.5 (21.7,41.8) | 1668 (1097,2258) | 28.5 (19.5,37.7) | -9.4 (-25.3,10.8) |
| 130 | American Samoa | 4 (3,5) | 19.8 (14.6,24.7) | 6 (5,7) | 13.1 (10.7,15.7) | -34 (-48.6, -12) |
| 131 | Cook Islands | 11 (9,14) | 102 (80.8,124.8) | 15 (13,19) | 65.3 (54.3,78.1) | -36 (-50.2, -14.3) |
| 132 | Fiji | 111 (84,142) | 37.9 (29,47.7) | 182 (143,231) | 30.2 (24.3,38) | -20.2 (-42.8,14.1) |
| 133 | Guam | 34 (20,39) | 61.3 (36.7,71.6) | 31 (25,39) | 16.3 (13.1,20.5) | -73.3 (-80, -47.7) |
| 134 | Kiribati | 9 (7,11) | 26.1 (20,31.9) | 12 (9,15) | 20.2 (15.5,25.5) | -22.6 (-41.8,1.8) |
| 135 | Marshall Islands | 6 (4,8) | 45.2 (29.4,61.6) | 11 (6,16) | 40.6 (24.3,55.8) | -10.2 (-31.6,20) |
| 136 | Micronesia (Federated States of) | 18 (11,24) | 46.6 (29.9,60.9) | 25 (13,34) | 43.2 (25,58.5) | -7.3 (-36.9,23.7) |
| 137 | Nauru | 1 (1,2) | 38.3 (25.1,49.6) | 1 (1,2) | 33.9 (19.3,45.2) | -11.4 (-31.8,13.9) |
| 138 | Niue | 1 (0,1) | 28.2 (19,37.3) | 0 (0,1) | 21.7 (13.1,28.8) | -23 (-42.6,0.5) |
| 139 | Northern Mariana Islands | 2 (1,2) | 12.4 (7.8,16) | 3 (2,3) | 6.8 (5.4,8.2) | -44.8 (-58.8, -19.5) |
| 140 | Palau | 0 (0,0) | 4.2 (2.9,5.7) | 1 (0,1) | 3.2 (2.4,4) | -23.9 (-48.1,13.4) |
| 141 | Papua New Guinea | 429 (253,639) | 28.5 (17,42) | 1117 (653,1625) | 29.2 (17.6,41.4) | 2.5 (-20.6,34.4) |
| 142 | Samoa | 25 (18,32) | 33.4 (23.8,42) | 36 (22,47) | 27.1 (17.2,35.2) | -19 (-38.8,6.6) |
| 143 | Solomon Islands | 43 (26,61) | 35.8 (22.6,49) | 93 (51,139) | 34.7 (20.1,49.2) | -3.2 (-30.1,27.4) |
| 144 | Tokelau | 0 (0,1) | 32.8 (21.9,46) | 0 (0,0) | 22.9 (14.6,30.5) | -30.1 (-47.6, -3.3) |
| 145 | Tonga | 4 (3,6) | 8.8 (6.2,11.5) | 6 (4,8) | 7.6 (5.7,9.9) | -13.5 (-36.9,16.4) |
| 146 | Tuvalu | 2 (2,3) | 41.1 (26.5,56.4) | 3 (2,4) | 30.3 (18.6,41.7) | -26.4 (-47.6,4) |
| 147 | Vanuatu | 17 (11,23) | 30.6 (19.6,41.2) | 48 (28,67) | 32 (18.4,43.8) | 4.6 (-22.8,43.9) |
| 148 | South Asia | 72846 (44041,99373) | 19 (12.1,26.7) | 146322 (103826,191490) | 13.2 (9.3,17.4) | -30.9 (-45.8, -5) |
| 149 | Bangladesh | 7908 (4303,11058) | 21.5 (12.2,30.7) | 16700 (10103,26173) | 15.9 (9.6,24.5) | -26.2 (-52,20.9) |
| 150 | Bhutan | 33 (18,49) | 19.9 (11.1,31.2) | 69 (50,94) | 15.2 (11,20.3) | -23.6 (-55.8,27.4) |
| 151 | India | 53791 (33171,75757) | 18.2 (11.6,26.2) | 110267 (73272,148670) | 12.1 (8,16.3) | -33.5 (-47.6, -8.9) |
| 152 | Nepal | 1068 (678,1781) | 15.6 (10.2,28.6) | 2435 (1758,3397) | 14.3 (10.4,20.2) | -8.6 (-38.8,36.8) |
| 153 | Pakistan | 10046 (6233,14262) | 20.9 (13.1,30.5) | 16850 (11249,22098) | 21 (14.4,27.7) | 0.3 (-24.6,26.8) |
| 154 | Southeast Asia | 51627 (37064,60881) | 25 (18.6,29.7) | 110441 (70668,124533) | 21.4 (14.2,24) | -14.5 (-30.1,4.5) |
| 155 | Cambodia | 1201 (690,1623) | 32.5 (18.9,44) | 2629 (1629,3376) | 26.9 (17.1,33.9) | -17.2 (-43.2,26.5) |
| 156 | Indonesia | 23667 (14496,29627) | 30.5 (19.4,38.2) | 50621 (28707,61825) | 30.8 (18.4,37.1) | 1 (-19.4,26.5) |
| 157 | Lao People's Democratic Republic | 634 (330,966) | 38.1 (21,57.6) | 958 (520,1356) | 27.9 (15.2,38.9) | -26.9 (-49.7,16.4) |
| 158 | Malaysia | 429 (334,508) | 5.5 (4.3,6.5) | 600 (457,806) | 2.7 (2,3.6) | -50.8 (-65.8, -20.9) |
| 159 | Maldives | 8 (3,11) | 12.1 (5.5,17.8) | 17 (13,21) | 7.2 (5.3,8.9) | -40.5 (-63.6,29.8) |
| 160 | Mauritius | 209 (160,229) | 34.7 (26.9,38.1) | 417 (321,517) | 26.8 (20.4,33.2) | -22.6 (-36.5, -3.5) |
| 161 | Myanmar | 6594 (3857,9338) | 34.1 (20.8,47.5) | 9013 (5437,11853) | 23.2 (14.6,30.1) | -31.8 (-50.4,4.7) |
| 162 | Philippines | 4901 (4329,5744) | 23.4 (20.7,27.3) | 23537 (17145,28081) | 36.2 (28.1,42.5) | 54.3 (6.1,87.8) |
| 163 | Seychelles | 41 (33,46) | 73.4 (59.4,81.8) | 43 (36,50) | 45.8 (38.6,53.4) | -37.6 (-46.8, -13.4) |
| 164 | Sri Lanka | 1541 (1348,1935) | 17.4 (15.2,22.9) | 3734 (2617,4997) | 16.5 (11.9,22) | -5.3 (-39.7,31.7) |
| 165 | Thailand | 951 (699,1264) | 3.5 (2.5,4.5) | 2171 (1606,2870) | 2.2 (1.6,2.9) | -35.7 (-55.8, -4.8) |
| 166 | Timor-Leste | 52 (30,78) | 26.2 (15.2,39) | 190 (109,283) | 28.7 (17,42.4) | 9.7 (-24.2,70.3) |
| 167 | Viet Nam | 11330 (7394,14666) | 32.1 (21.4,41.8) | 16366 (9698,23527) | 21.1 (12.7,30) | -34.2 (-57.5, -1) |
| 168 | Southern Latin America | 5916 (5318,7225) | 14.2 (12.6,18) | 11973 (9823,13736) | 13.9 (11.4,15.9) | -2.6 (-22.7,8.4) |
| 169 | Uruguay | 318 (281,384) | 8.3 (7.3,10.2) | 628 (438,721) | 9.6 (6.8,10.9) | 15.4 (-21.5,32.6) |
| 170 | Argentina | 4525 (4056,5657) | 15.4 (13.7,20) | 8325 (7104,10094) | 14.8 (12.7,18) | -3.5 (-18.6,8.6) |
| 171 | Chile | 1073 (872,1181) | 13.2 (10.9,14.5) | 3019 (2014,3492) | 12.8 (8.5,14.8) | -3.2 (-33.5,10.7) |
| 172 | Southern Sub-Saharan Africa | 7386 (6453,8766) | 31.7 (27.2,37.9) | 15244 (13548,17064) | 34.1 (30.1,38.1) | 7.8 (-10.6,20.2) |
| 173 | Botswana | 179 (124,242) | 40.6 (28.8,54.8) | 404 (265,568) | 40.4 (26.2,56.2) | -0.5 (-38.3,50.6) |
| 174 | Eswatini | 107 (82,134) | 46 (35.7,57.3) | 220 (141,322) | 51 (34,71.8) | 10.9 (-25.3,57.3) |
| 175 | Lesotho | 344 (266,437) | 42.4 (33.1,54.3) | 601 (410,817) | 62.1 (43.2,82.2) | 46.4 (0.8,104.6) |
| 176 | Namibia | 262 (188,343) | 44.7 (32.1,58.8) | 478 (337,660) | 41.3 (29.7,56.4) | -7.7 (-38.1,31.6) |
| 177 | South Africa | 5754 (4962,7128) | 31.5 (26.8,39) | 12384 (10716,13901) | 34.3 (29.3,38.2) | 8.9 (-11.3,22.6) |
| 178 | Zimbabwe | 740 (602,1025) | 24.1 (19.4,33.9) | 1157 (804,1917) | 21.8 (15.5,36.4) | -9.5 (-34.4,26.1) |
| 179 | Tropical Latin America | 16305 (12899,17349) | 21.9 (17.9,23.4) | 30651 (26652,41664) | 13.4 (11.6,18.2) | -38.6 (-44.3, -7.7) |
| 180 | Brazil | 16024 (12645,17080) | 22.1 (18,23.7) | 29812 (25857,40911) | 13.4 (11.5,18.3) | -39.5 (-45.4, -8.3) |
| 181 | Paraguay | 281 (224,319) | 14.2 (11.1,16.2) | 839 (605,1062) | 16.3 (11.6,20.7) | 14.3 (-13.2,46.7) |
| 182 | Western Europe | 47171 (40735,55755) | 8 (6.8,9.7) | 101523 (68545,115189) | 8.4 (5.8,9.5) | 4.5 (-31.7,15.3) |
| 183 | Andorra | 4 (3,6) | 12.6 (8.9,17.2) | 16 (12,21) | 9.6 (7,12.4) | -24 (-47,8.5) |
| 184 | Austria | 1202 (1060,1618) | 9.9 (8.7,13.7) | 2985 (1683,3497) | 12.8 (7.4,14.9) | 29.5 (-39.3,51.1) |
| 185 | Belgium | 471 (406,694) | 3.1 (2.6,4.6) | 876 (685,1112) | 2.8 (2.2,3.7) | -8.1 (-31.2,6.4) |
| 186 | Cyprus | 97 (52,130) | 20.6 (10.2,27.5) | 179 (91,214) | 13.2 (6.1,15.9) | -35.8 (-53.9,14.3) |
| 187 | Denmark | 240 (200,277) | 2.8 (2.3,3.2) | 301 (244,368) | 2.3 (1.8,2.8) | -18.6 (-31.9,6.1) |
| 188 | Finland | 450 (381,536) | 6.5 (5.5,7.8) | 1451 (746,1720) | 9.6 (4.9,11.3) | 48.2 (-31.6,73.7) |
| 189 | France | 5165 (3514,5791) | 5.8 (4,6.5) | 8543 (5427,10391) | 4.1 (2.8,4.9) | -29.7 (-40.5, -7.8) |
| 190 | Germany | 18281 (15974,23772) | 13.9 (12,18.4) | 34349 (26138,40003) | 14.2 (10.9,16.5) | 2.4 (-30.5,17.7) |
| 191 | Greece | 1117 (949,1350) | 8.2 (6.9,9.8) | 3006 (2089,3514) | 9.1 (6.5,10.6) | 11.4 (-21,29) |
| 192 | Iceland | 11 (9,14) | 3.4 (2.9,4.4) | 23 (17,28) | 3.2 (2.4,3.9) | -6.3 (-33.7,10.8) |
| 193 | Ireland | 143 (126,179) | 3.9 (3.4,4.9) | 190 (152,278) | 2.4 (1.9,3.5) | -37.6 (-47.4, -12.9) |
| 194 | Israel | 347 (190,396) | 8 (4.6,9.1) | 257 (187,629) | 1.9 (1.4,4.8) | -75.7 (-81.7, -8.4) |
| 195 | Italy | 11215 (8368,12184) | 13.1 (10.2,14.6) | 28201 (15317,32325) | 13.9 (7.8,15.9) | 6.7 (-34.6,17.7) |
| 196 | Luxembourg | 28 (24,37) | 5.7 (4.8,7.5) | 56 (42,73) | 4.7 (3.5,6) | -17.8 (-40.8,0.2) |
| 197 | Malta | 26 (22,33) | 7.1 (6,9) | 52 (40,65) | 4.9 (3.9,6.3) | -30.7 (-43.1, -14.8) |
| 198 | Monaco | 4 (3,6) | 4.8 (3.7,6.6) | 8 (6,9) | 5.9 (4.5,7.2) | 23.2 (-12.8,61.6) |
| 199 | Netherlands | 509 (436,707) | 2.5 (2.2,3.5) | 1130 (810,1334) | 2.9 (2.1,3.4) | 14.3 (-27.4,33.8) |
| 200 | Norway | 262 (195,285) | 3.5 (2.6,3.8) | 242 (192,337) | 2 (1.6,2.9) | -42.4 (-50.3, -0.3) |
| 201 | Portugal | 884 (603,977) | 7.6 (5.3,8.4) | 1990 (1167,2369) | 6.3 (3.8,7.4) | -17.5 (-39.7, -2.5) |
| 202 | San Marino | 2 (1,3) | 6.1 (4.8,8.3) | 6 (4,9) | 7.2 (5,9.9) | 18.1 (-25.1,73.4) |
| 203 | Spain | 2270 (1920,2829) | 4.5 (3.7,5.7) | 8727 (4525,10420) | 6.3 (3.4,7.5) | 41.8 (-31.1,69) |
| 204 | Sweden | 353 (299,606) | 2.1 (1.8,3.6) | 1724 (662,2053) | 6.2 (2.5,7.3) | 190.8 (-21.1,257.5) |
| 205 | Switzerland | 851 (416,1043) | 7.6 (3.7,9.2) | 2222 (828,2757) | 9 (3.6,11) | 19 (-22.2,45.8) |
| 206 | United Kingdom | 3200 (2853,5488) | 3.5 (3.1,6) | 4901 (4192,7731) | 3.5 (3,5.2) | 0.1 (-24.3,7.1) |
| 207 | Western Sub-Saharan Africa | 13658 (10159,17327) | 18.5 (14,23.3) | 29625 (18109,37337) | 18.9 (11.8,23.5) | 2.1 (-37.5,28.9) |
| 208 | Benin | 313 (228,394) | 17.2 (12.7,21.7) | 885 (517,1246) | 21.4 (12.6,29.3) | 24.4 (-19.5,74.9) |
| 209 | Burkina Faso | 702 (488,936) | 18.7 (13.3,25) | 2243 (1260,2984) | 29.3 (16.7,38.3) | 56.4 (-8.9,110.2) |
| 210 | Cabo Verde | 32 (23,40) | 13.7 (10.2,17.2) | 59 (43,73) | 14.1 (10.3,17.2) | 3.5 (-31.4,45.6) |
| 211 | Cameroon | 715 (450,972) | 19.2 (12.3,26.3) | 1958 (1057,3004) | 19.9 (10.9,29.4) | 3.9 (-34.9,53.7) |
| 212 | Chad | 459 (311,629) | 18.2 (12.5,24.9) | 1127 (608,1553) | 23.4 (12.7,31.8) | 28.3 (-15.8,74.4) |
| 213 | C么te d'Ivoire | 512 (343,683) | 16.5 (11.2,21.6) | 1745 (950,2546) | 20.8 (11.4,29.4) | 25.6 (-20,75.5) |
| 214 | Gambia | 43 (30,58) | 15.5 (11.1,20.3) | 200 (107,290) | 24.3 (13,34.9) | 56.8 (-3.1,125.5) |
| 215 | Ghana | 860 (590,1122) | 16.9 (12,21.7) | 2583 (1525,3398) | 19.8 (11.8,25.5) | 17 (-26.5,57.2) |
| 216 | Guinea | 551 (330,756) | 18.5 (11.5,25.7) | 1223 (627,1681) | 24.8 (12.9,33.6) | 33.8 (-20,97.1) |
| 217 | Guinea-Bissau | 87 (43,119) | 24.6 (13.7,33.2) | 185 (87,268) | 30.3 (15.1,43.2) | 23.2 (-23,78.8) |
| 218 | Liberia | 165 (102,220) | 17.1 (11,22.8) | 364 (191,560) | 21 (11,31.6) | 22.6 (-24.6,77.7) |
| 219 | Mali | 704 (408,960) | 20.4 (12,27.6) | 1583 (904,2122) | 21.8 (12.6,28.7) | 6.9 (-31,45.2) |
| 220 | Mauritania | 176 (118,233) | 19.9 (13.6,26.2) | 300 (182,407) | 16.5 (10.1,22.1) | -17 (-48.6,18.4) |
| 221 | Niger | 377 (231,546) | 16.7 (10.2,25.5) | 1129 (628,1716) | 17.8 (9.9,26.6) | 6.6 (-31.8,46.4) |
| 222 | Nigeria | 7137 (4985,9917) | 19.4 (13.6,26.5) | 11660 (7218,15659) | 16.2 (10.1,21.4) | -16.3 (-53.1,19) |
| 223 | Sao Tome and Principe | 5 (4,6) | 9.2 (7.3,11.4) | 7 (5,10) | 8.3 (5.5,10.8) | -10.1 (-36.3,24.9) |
| 224 | Senegal | 411 (292,517) | 14.9 (10.7,18.7) | 1186 (658,1633) | 18.5 (10.3,25.2) | 23.9 (-20.4,68.8) |
| 225 | Sierra Leone | 231 (156,314) | 13.6 (9.3,18.4) | 566 (313,804) | 18.3 (10.3,25.6) | 34.6 (-16.4,86.6) |
| 226 | Togo | 177 (132,224) | 17 (12.7,21.5) | 623 (350,852) | 21.4 (12.3,29.1) | 26.2 (-20.4,78) |

**Table S3**: DALYs due to hypertensive heart disease in 1990 and 2019 and the percentage change in the age-standardized rates (ASRs) per 100,000 people by location

|  | **Table S3: DALYs due to hypertensive heart disease in 1990 and 2019 and the percentage change in  the age-standardized rates (ASRs) per 100,000, by location** | | | | | |
| --- | --- | --- | --- | --- | --- | --- |
|  | Location | 1990 |  | 2019 |  | Percentage  change in the  ASRs per  100,000 |
|  |  | No. (95% UI) | ASRs per 100,000 (95% UI) | No. (95% UI) | ASRs per 100,000 (95% UI) |  |
| 1 | Global | 13943612 (11312107,15650103) | 364.6 (297.7,406.7) | 21508002 (16400051,23899879) | 268.2 (204.6,298.1) | -26.4 (-35.5, -15.7) |
| 2 | Andean Latin America | 47183 (40474,53915) | 232.3 (199.5,264.9) | 90914 (74602,108263) | 164.6 (134.9,195.7) | -29.1 (-43.2, -13.3) |
| 3 | Bolivia (Plurinational State of) | 11658 (7040,15778) | 380 (233.6,512.8) | 23908 (16612,33016) | 293.3 (206.4,400.1) | -22.8 (-46.4,10.5) |
| 4 | Ecuador | 19861 (18243,21901) | 382.4 (348.8,418.8) | 45150 (35073,55980) | 318.9 (248.5,393) | -16.6 (-35.8,3.9) |
| 5 | Peru | 15664 (12954,18318) | 130 (108.6,152.9) | 21856 (16546,29378) | 67.3 (50.9,90) | -48.2 (-62.3, -26.9) |
| 6 | Australasia | 13670 (10801,15255) | 60.5 (47.9,67.4) | 20379 (16878,24230) | 38.9 (32.9,46.6) | -35.6 (-41, -9.9) |
| 7 | Australia | 10721 (8704,12101) | 57.2 (46.6,64.7) | 17007 (13925,19956) | 38.4 (32,45.2) | -32.8 (-39.5, -10.6) |
| 8 | New Zealand | 2949 (2100,3274) | 77.3 (55.5,85.7) | 3372 (2862,4566) | 41.9 (35.9,56.1) | -45.8 (-53.1, -7.2) |
| 9 | Caribbean | 100652 (86727,120565) | 388.8 (336.2,467.5) | 192423 (158186,229234) | 372.3 (306.2,444.1) | -4.2 (-20.1,12.6) |
| 10 | Antigua and Barbuda | 293 (253,328) | 544.4 (471.3,609.8) | 526 (414,630) | 537 (425.2,637.9) | -1.4 (-17.5,17.1) |
| 11 | Bahamas | 1709 (1506,2007) | 1094.3 (966.5,1282.9) | 4262 (3368,5400) | 1089.4 (866.6,1363.3) | -0.4 (-20.3,24.6) |
| 12 | Barbados | 832 (716,947) | 285.6 (245.8,322.5) | 1259 (1015,1561) | 260 (210.1,323) | -9 (-25,10.8) |
| 13 | Belize | 334 (291,402) | 360.2 (314.5,435) | 1090 (887,1289) | 393.4 (321.9,463.7) | 9.2 (-9.8,30.9) |
| 14 | Bermuda | 111 (86,126) | 183.4 (141.3,207.1) | 165 (126,203) | 125.5 (96.9,156.1) | -31.6 (-47.7, -9.8) |
| 15 | Cuba | 12127 (10955,16059) | 118.7 (107.2,158.1) | 39938 (21577,50236) | 209.3 (113.9,263.5) | 76.4 (-17.2,128.3) |
| 16 | Dominica | 691 (605,778) | 958.1 (844.8,1078.1) | 733 (599,909) | 817.5 (667.4,1013.1) | -14.7 (-32.1,8.6) |
| 17 | Dominican Republic | 13711 (12015,15855) | 377.4 (329.8,436.6) | 36699 (26287,49404) | 401.7 (293.2,536.5) | 6.4 (-25.4,44.9) |
| 18 | Grenada | 389 (333,463) | 537.6 (458.4,642.5) | 531 (429,614) | 491.1 (394.8,564.8) | -8.6 (-25.3,7.5) |
| 19 | Guyana | 6061 (4555,6989) | 1610.6 (1207.2,1843.6) | 7259 (5517,9501) | 1157.4 (894.9,1490) | -28.1 (-46.2, -3.3) |
| 20 | Haiti | 30708 (19946,49020) | 950.9 (628.6,1653.7) | 52948 (30957,82840) | 762.5 (450.2,1181) | -19.8 (-44.5,15.2) |
| 21 | Jamaica | 12982 (8250,14270) | 721 (461.2,791.3) | 13706 (10878,18005) | 451.1 (357.4,594.8) | -37.4 (-52.4,7.7) |
| 22 | Puerto Rico | 8337 (7528,10364) | 232.6 (210.5,288) | 15420 (11648,19707) | 229.5 (168.4,294.7) | -1.3 (-30.1,27.2) |
| 23 | Saint Kitts and Nevis | 210 (169,240) | 582.2 (466,661.7) | 292 (229,361) | 466.3 (371.5,571.7) | -19.9 (-37,2) |
| 24 | Saint Lucia | 512 (429,574) | 609.9 (509.3,683.8) | 890 (736,1090) | 420.4 (346.5,511.5) | -31.1 (-42.7, -13.4) |
| 25 | Saint Vincent and the Grenadines | 497 (436,574) | 705.5 (615.5,813.9) | 879 (735,1038) | 670.5 (557.4,788.3) | -5 (-19.4,11.7) |
| 26 | Suriname | 1376 (1199,1521) | 534.8 (467.3,590.5) | 2587 (2085,3144) | 437 (351.5,530.3) | -18.3 (-34.1,1.9) |
| 27 | Trinidad and Tobago | 6009 (4350,6517) | 736.9 (530.8,800.6) | 5851 (4283,9807) | 323.2 (236.8,539.1) | -56.1 (-68.4, -13) |
| 28 | United States Virgin Islands | 410 (334,511) | 493.9 (405.2,611.6) | 870 (723,1031) | 489.1 (407.8,575) | -1 (-24.7,27.4) |
| 29 | Central Asia | 147407 (131117,191112) | 325.3 (288.5,423.3) | 292503 (252571,334956) | 454.6 (390,515.3) | 39.7 (8.2,68.9) |
| 30 | Armenia | 5138 (3209,6278) | 229.2 (125.4,287.1) | 9351 (5080,11220) | 233.4 (123.4,280) | 1.9 (-19.1,28.9) |
| 31 | Azerbaijan | 25511 (21107,32608) | 533.7 (439,692) | 39258 (29074,52599) | 510.5 (373.7,682.3) | -4.4 (-42.7,36.7) |
| 32 | Georgia | 15203 (11290,32261) | 265 (197.8,541.5) | 54457 (28143,65637) | 860.1 (465,1036.7) | 224.6 (2.2,370.6) |
| 33 | Kazakhstan | 36371 (30522,57882) | 285.9 (238.9,480.8) | 46662 (36912,87495) | 275.4 (217.9,531.3) | -3.7 (-21.9,21.1) |
| 34 | Kyrgyzstan | 6890 (5668,8558) | 229.1 (191.8,286.6) | 11146 (8257,13280) | 252.2 (193.6,299.7) | 10.1 (-14.5,35.3) |
| 35 | Mongolia | 4999 (2839,6173) | 484.7 (279.2,593.1) | 4767 (3532,6157) | 211 (161.8,267.9) | -56.5 (-67.9, -28.8) |
| 36 | Tajikistan | 19105 (13555,25397) | 695.7 (489.6,943.2) | 35536 (22959,44476) | 873.6 (556.4,1085.4) | 25.6 (-7.4,64.4) |
| 37 | Turkmenistan | 5225 (4492,6317) | 282.6 (242.6,341.1) | 11999 (9155,15452) | 324.7 (250.5,415.8) | 14.9 (-12.4,49.1) |
| 38 | Uzbekistan | 28966 (23642,36069) | 262.3 (213.7,327.4) | 79326 (55933,102698) | 462.2 (311.8,622.6) | 76.2 (13.3,168.6) |
| 39 | Central Europe | 466530 (437891,562125) | 328.3 (306.6,398.8) | 730907 (546291,846682) | 334.8 (249.4,387.4) | 2 (-28.4,17.6) |
| 40 | Albania | 3181 (2848,3620) | 174.3 (155.6,197.4) | 4244 (3176,5603) | 99.3 (74.6,130.8) | -43 (-57.9, -23.1) |
| 41 | Bosnia and Herzegovina | 7886 (7181,9379) | 208.5 (188.9,264.8) | 8293 (6516,12466) | 144.6 (114.1,213.7) | -30.7 (-45.2, -11.2) |
| 42 | Bulgaria | 67618 (60009,113044) | 569.7 (504.6,965) | 178217 (110819,224814) | 1238.8 (758.5,1564.6) | 117.4 (-8.5,195.1) |
| 43 | Croatia | 19629 (14512,21494) | 332 (240.2,364) | 22904 (16244,28551) | 247 (178.2,309.9) | -25.6 (-40.9, -3.7) |
| 44 | Czechia | 9964 (8256,21582) | 74 (61.5,159.5) | 25058 (18606,30943) | 119.8 (88.5,149.3) | 61.8 (-41.4,121.7) |
| 45 | Hungary | 65059 (57599,74438) | 456 (400.9,522.8) | 85437 (59092,104960) | 436 (302.5,538.3) | -4.4 (-34.4,16.6) |
| 46 | Montenegro | 409 (356,490) | 69.2 (60.3,82.5) | 778 (624,954) | 81.3 (65.1,99.5) | 17.5 (-11,49.8) |
| 47 | North Macedonia | 9837 (8750,11309) | 634 (557.6,736.9) | 15134 (11915,18378) | 561 (441,673.3) | -11.5 (-31.3,8.9) |
| 48 | Poland | 90881 (83086,110286) | 213.7 (196.8,262.3) | 134540 (105317,162957) | 189.4 (147.2,229.9) | -11.4 (-32.7,6) |
| 49 | Romania | 145342 (122020,156182) | 553.4 (473.3,594.9) | 183387 (144396,223297) | 469.8 (369.8,573) | -15.1 (-30.2,2.1) |
| 50 | Serbia | 32221 (27558,40228) | 329.7 (281.1,410.6) | 43824 (34155,53394) | 290 (222.3,351.3) | -12 (-35.8,12.3) |
| 51 | Slovakia | 8134 (6959,13986) | 138.8 (118.5,238.4) | 18829 (13526,23682) | 207.3 (147.7,260.5) | 49.3 (-35.4,100.5) |
| 52 | Slovenia | 6369 (4369,8142) | 266 (181,339.8) | 10262 (5620,13188) | 202.7 (120.3,262.4) | -23.8 (-48.6,4.4) |
| 53 | Central Latin America | 224846 (161060,238460) | 288.8 (208.2,307.2) | 392457 (329203,503101) | 171.5 (144.2,219.6) | -40.6 (-50.1, -6.3) |
| 54 | Colombia | 74202 (42161,81096) | 461.3 (259.5,506.2) | 82279 (62285,131041) | 152.8 (115.3,244.9) | -66.9 (-75.4, -11.8) |
| 55 | Costa Rica | 3285 (2733,3648) | 195.6 (160.1,218.3) | 8518 (6707,10845) | 166.1 (131.1,211.6) | -15.1 (-32.7,7.7) |
| 56 | El Salvador | 4558 (4116,5339) | 151.6 (136.3,181.6) | 6668 (5144,8523) | 109.3 (84,139.7) | -27.9 (-45.8, -6.2) |
| 57 | Guatemala | 6713 (5058,7656) | 189.5 (157.6,214.5) | 12380 (9653,17502) | 117.3 (92,170.2) | -38.1 (-51.7, -4.7) |
| 58 | Honduras | 6302 (5126,8789) | 291.4 (234.7,444.5) | 16821 (11355,26345) | 285.5 (192.5,464.2) | -2 (-35.2,30.5) |
| 59 | Mexico | 73037 (54970,78062) | 190.5 (140.9,204.3) | 151034 (126033,177586) | 136.6 (113,160.3) | -28.3 (-37.7, -10.2) |
| 60 | Nicaragua | 3904 (3447,4344) | 271.4 (236.6,303.4) | 11116 (8911,13194) | 286 (230.8,334.7) | 5.4 (-14.3,25.3) |
| 61 | Panama | 1501 (1316,2006) | 102.1 (89.1,138.1) | 5249 (3917,6664) | 125.6 (93.9,159.5) | 23.1 (-16.9,60) |
| 62 | Venezuela (Bolivarian Republic of) | 51343 (37232,55231) | 547.7 (406.1,591.8) | 98393 (73622,136259) | 350.8 (263.9,486.7) | -35.9 (-52.4,1.5) |
| 63 | Central Sub-Saharan Africa | 250003 (156306,325981) | 1143.1 (733.8,1487.5) | 479608 (307612,648098) | 970.3 (625.8,1310.4) | -15.1 (-33.3,7.3) |
| 64 | Angola | 43343 (27053,58283) | 1134.6 (722.6,1537) | 82090 (50088,111005) | 799.7 (488,1071.5) | -29.5 (-49.3,0.5) |
| 65 | Central African Republic | 16777 (10015,22781) | 1474.7 (912.7,2015.2) | 27077 (15613,38164) | 1321.8 (787.6,1924.4) | -10.4 (-33.8,23.1) |
| 66 | Congo | 14989 (9029,19481) | 1452.5 (903.4,1864.7) | 24016 (15253,32280) | 984.8 (633.5,1307.6) | -32.2 (-49.7, -7.4) |
| 67 | Democratic Republic of the Congo | 166007 (99032,224584) | 1093.7 (675.4,1495.4) | 335081 (205668,474992) | 1007.4 (627,1419.5) | -7.9 (-30.6,23.3) |
| 68 | Equatorial Guinea | 2637 (1561,3627) | 1357.7 (827.2,1887.5) | 2933 (1658,4385) | 672.7 (387.7,958.5) | -50.5 (-69.5, -16.8) |
| 69 | Gabon | 6250 (3919,8116) | 1171.7 (729.9,1509.1) | 8411 (5333,11263) | 867.6 (555.1,1137.8) | -26 (-44.6,1.1) |
| 70 | East Asia | 5144375 (3759946,5899064) | 680 (516,768.8) | 5780823 (4053566,6713463) | 310.4 (217.5,359.4) | -54.4 (-63.6, -44.7) |
| 71 | China | 5042174 (3661268,5792671) | 692.6 (526.3,786) | 5594910 (3877275,6532987) | 312.9 (214.1,363.7) | -54.8 (-64.4, -44.9) |
| 72 | Democratic People's Republic of Korea | 53240 (35923,75485) | 386.3 (264.2,537.8) | 114559 (89000,146040) | 376.1 (293.9,479.9) | -2.6 (-30.3,38) |
| 73 | Taiwan (Province of China) | 48960 (32615,53118) | 358.1 (244,389.6) | 71354 (56967,96024) | 179.7 (143.6,241.4) | -49.8 (-60.5, -14.2) |
| 74 | Eastern Europe | 225457 (202493,326682) | 82.7 (74,120) | 447153 (297646,517284) | 131.7 (88.5,152.6) | 59.3 (-6.5,85.1) |
| 75 | Belarus | 13980 (8416,17812) | 108.6 (65.8,138.2) | 7873 (5687,13606) | 51.4 (37.2,86.4) | -52.7 (-67,10.1) |
| 76 | Estonia | 3366 (2730,7279) | 166.4 (134.9,360.5) | 26713 (7083,34967) | 953.3 (273.9,1252.7) | 473 (-17.1,748.4) |
| 77 | Latvia | 1685 (1319,4150) | 47.6 (37.4,117.2) | 10052 (3207,13058) | 247.7 (84.2,321.6) | 420.1 (-15.9,697.3) |
| 78 | Lithuania | 3540 (2966,6044) | 79.3 (66.4,135.2) | 8991 (5250,11345) | 166.3 (94.4,212.6) | 109.8 (-18.5,184.8) |
| 79 | Republic of Moldova | 4245 (3484,8996) | 98.3 (80.8,211.4) | 25285 (8276,31266) | 432.7 (144.2,534.9) | 340 (-24.3,502.8) |
| 80 | Russian Federation | 181301 (162993,262454) | 102.6 (92,150.3) | 346150 (234413,407628) | 149.7 (102.7,176.5) | 45.9 (-11.5,72.5) |
| 81 | Ukraine | 17339 (14154,28351) | 26.2 (21.5,40.8) | 22089 (17952,33273) | 31.9 (26.2,44.9) | 21.9 (-6,53.8) |
| 82 | Eastern Sub-Saharan Africa | 751971 (457843,1090488) | 1013.4 (625.8,1450.8) | 1077393 (658673,1587143) | 696.9 (418.4,1038.7) | -31.2 (-43.4, -9.5) |
| 83 | Burundi | 23673 (4280,41586) | 1011 (183.1,1779) | 28005 (12229,50666) | 651.7 (282.8,1155.6) | -35.5 (-56.3,76.3) |
| 84 | Comoros | 2137 (1042,3215) | 986.4 (504.6,1475.2) | 3113 (1822,4805) | 666.6 (393.8,1027) | -32.4 (-52.1,11.6) |
| 85 | Djibouti | 1336 (790,1974) | 977.4 (598.7,1417) | 3540 (2059,5581) | 650.4 (384.7,977.3) | -33.5 (-51.6, -5.3) |
| 86 | Eritrea | 13401 (7212,20677) | 1331.1 (734.1,2158) | 24791 (13136,40333) | 998 (550.8,1610.2) | -25 (-50.5,10.1) |
| 87 | Ethiopia | 241920 (125514,369004) | 1204.1 (684.4,1795.4) | 218055 (126118,355861) | 559.7 (322.5,920.4) | -53.5 (-66.8, -19.7) |
| 88 | Kenya | 47026 (29828,72487) | 595.2 (379,902.8) | 113752 (70812,179984) | 564.7 (343.2,903.3) | -5.1 (-20.9,13.5) |
| 89 | Madagascar | 77182 (47555,106996) | 1433.8 (883.5,1987.1) | 140163 (82638,206185) | 1258.8 (740.1,1886.4) | -12.2 (-34.6,18.9) |
| 90 | Malawi | 30794 (18087,44985) | 835.9 (501.1,1214.3) | 44403 (25724,71347) | 653 (378,1037.4) | -21.9 (-41.5,10.6) |
| 91 | Mozambique | 53836 (32898,78363) | 962.8 (599.9,1418.9) | 91174 (55237,135523) | 899.4 (543.1,1336.3) | -6.6 (-31.6,29.5) |
| 92 | Rwanda | 36078 (10938,58124) | 1242.6 (378.7,2090.5) | 32573 (10365,57997) | 591.5 (185.1,1040) | -52.4 (-66.7, -26) |
| 93 | Somalia | 36273 (19924,52476) | 1386.3 (804.6,1956.1) | 64079 (38733,96914) | 1012.3 (619.3,1508.1) | -27 (-47.2,3.9) |
| 94 | South Sudan | 20919 (11822,31927) | 899.3 (510.4,1353.8) | 20660 (11915,35668) | 573.8 (335.6,989.7) | -36.2 (-54.4, -7.9) |
| 95 | Uganda | 42624 (18119,73285) | 686.6 (295.5,1167.8) | 77507 (29993,132011) | 583.2 (222.5,995.7) | -15.1 (-34.7,8.2) |
| 96 | United Republic of Tanzania | 95914 (58530,138843) | 926.8 (568.8,1342.4) | 149720 (78343,240069) | 652.3 (339,1044.3) | -29.6 (-46.5, -7.6) |
| 97 | Zambia | 28307 (18342,37071) | 1016.3 (668.5,1308.7) | 64997 (39870,88045) | 1034.1 (635.3,1388.8) | 1.8 (-26.3,41.5) |
| 98 | High-income Asia Pacific | 234464 (153052,252777) | 129.2 (81.3,139.9) | 231326 (187431,320808) | 44 (36.5,64.6) | -65.9 (-71, -28.6) |
| 99 | Brunei Darussalam | 385 (313,466) | 445.3 (371.4,541.4) | 631 (532,891) | 275.3 (233.6,369.9) | -38.2 (-49.5, -22.3) |
| 100 | Japan | 155651 (89781,170072) | 101.5 (56.5,111.5) | 153506 (120503,221019) | 34.7 (28.1,56.1) | -65.8 (-71.3, -9.8) |
| 101 | Republic of Korea | 73426 (52809,79978) | 277.6 (214.5,304.3) | 62698 (51239,93096) | 77.4 (62.8,111.5) | -72.1 (-77.5, -52) |
| 102 | Singapore | 5003 (4068,5560) | 230.9 (199.4,266.9) | 14491 (9011,16687) | 184.9 (119,212.4) | -19.9 (-55.1, -7.6) |
| 103 | High-income North America | 525978 (451723,581933) | 155.3 (133.2,171.8) | 1101963 (735461,1201088) | 193.7 (126.5,210.2) | 24.7 (-14.5,32.4) |
| 104 | Canada | 12922 (11360,15564) | 40.7 (35.7,48.8) | 26072 (18699,29707) | 39.1 (29.3,44.4) | -3.9 (-33.6,7.7) |
| 105 | Greenland | 78 (65,95) | 207 (173.7,251) | 107 (83,135) | 156.1 (123.8,193.8) | -24.6 (-43.3, -2) |
| 106 | United States of America | 512965 (439534,567529) | 167.4 (143.2,184.8) | 1075767 (716054,1171660) | 212.2 (138.1,230.5) | 26.7 (-13,34.6) |
| 107 | North Africa and Middle East | 1153295 (702872,1469063) | 726.3 (448.4,913.1) | 2180416 (1285193,2768672) | 545 (315.8,682.4) | -25 (-42, -7.4) |
| 108 | Afghanistan | 107008 (31319,165653) | 1525.4 (462.1,2324.2) | 171933 (55322,257996) | 1374.1 (467.2,2020.7) | -9.9 (-34.3,24.1) |
| 109 | Algeria | 98681 (48274,137763) | 929.7 (447,1285.5) | 167586 (75889,225037) | 562.3 (246.6,750.9) | -39.5 (-55.2, -14) |
| 110 | Bahrain | 398 (332,477) | 219 (181.5,289.5) | 1053 (831,1334) | 121.3 (96.7,160.5) | -44.6 (-59, -25.4) |
| 111 | Egypt | 260227 (127619,396351) | 953 (460.2,1434.9) | 479769 (205145,839823) | 827.6 (337.7,1419.8) | -13.2 (-44.2,19.6) |
| 112 | Iran (Islamic Republic of) | 147285 (121914,188151) | 645.8 (531.2,785.7) | 340486 (302456,368968) | 498.6 (436.9,540.6) | -22.8 (-41.5, -5.7) |
| 113 | Iraq | 30668 (22468,39847) | 412 (302.6,532.3) | 64650 (50792,79396) | 314.7 (251.5,376.4) | -23.6 (-45.7,8.1) |
| 114 | Jordan | 11682 (9439,14257) | 1008.4 (800.4,1254.3) | 37419 (25921,45440) | 664.8 (440.8,798) | -34.1 (-53, -14.3) |
| 115 | Kuwait | 3817 (2919,4261) | 615.6 (483.9,701.9) | 9095 (7448,12545) | 371.5 (302.9,490.8) | -39.6 (-51.7, -3.2) |
| 116 | Lebanon | 12640 (6334,18986) | 600.8 (302.3,895.9) | 23993 (10723,31998) | 462.8 (205.5,616) | -23 (-55.3,19.7) |
| 117 | Libya | 8101 (4398,11341) | 440.8 (237,613.7) | 23150 (10915,33703) | 470.8 (222.8,681.1) | 6.8 (-27.7,53.9) |
| 118 | Morocco | 99548 (50854,135656) | 775.4 (387.8,1072.6) | 194965 (89377,268008) | 682.9 (305.9,920.6) | -11.9 (-35,23.2) |
| 119 | Oman | 3151 (2252,4341) | 519.7 (375.7,719.3) | 5651 (4239,7227) | 385.5 (296.4,461.1) | -25.8 (-51.5,10) |
| 120 | Palestine | 4440 (3265,5667) | 561.2 (418.1,706.1) | 11515 (8622,13520) | 587.4 (410.4,686.5) | 4.7 (-29.6,45.4) |
| 121 | Qatar | 194 (139,257) | 178 (129.8,248.1) | 640 (455,878) | 87.1 (64.4,114.3) | -51.1 (-68.7, -24.7) |
| 122 | Saudi Arabia | 7958 (5733,11530) | 122.9 (89.8,188.6) | 21505 (15241,27824) | 95.7 (73.3,120.8) | -22.1 (-55.6,14.6) |
| 123 | Sudan | 92313 (40839,127563) | 1017.4 (452.2,1393.8) | 149603 (64313,229914) | 845.6 (353.8,1262.8) | -16.9 (-39.9,19.2) |
| 124 | Syrian Arab Republic | 11143 (7851,14821) | 221.1 (159.5,297) | 16622 (12656,22279) | 152.8 (117.9,200.3) | -30.9 (-53.5,6.3) |
| 125 | Tunisia | 25604 (13891,32558) | 556.8 (298.9,711.3) | 52894 (24642,74461) | 443.5 (205.2,622.2) | -20.4 (-44.2,12.7) |
| 126 | Turkey | 171692 (119446,232639) | 543.6 (368,743) | 240376 (172860,294851) | 292.7 (204.4,360.6) | -46.2 (-63.8, -21.1) |
| 127 | United Arab Emirates | 3869 (1581,7452) | 865.1 (347.2,1576.1) | 37483 (13446,79415) | 802.3 (297.6,1587.5) | -7.3 (-46.6,64.8) |
| 128 | Yemen | 52101 (22008,75611) | 1123.5 (477.7,1597.7) | 127814 (53510,204622) | 1024.1 (426.8,1623.2) | -8.8 (-38.1,36.2) |
| 129 | Oceania | 21024 (14463,28574) | 686.4 (481.2,916.5) | 46429 (29882,63469) | 626.3 (419.1,841.6) | -8.8 (-25.9,13.6) |
| 130 | American Samoa | 96 (71,119) | 416.3 (311.7,509.7) | 139 (114,169) | 290.6 (239.1,350.1) | -30.2 (-46.1, -8.5) |
| 131 | Cook Islands | 273 (213,338) | 2136.7 (1691.8,2614) | 326 (268,394) | 1356.2 (1110.7,1654.8) | -36.5 (-51.6, -13.9) |
| 132 | Fiji | 3057 (2320,3946) | 810.2 (620.2,1029.5) | 4698 (3655,6029) | 633.9 (504.6,797.8) | -21.8 (-44.5,13.5) |
| 133 | Guam | 834 (529,975) | 1142 (708.5,1328.2) | 798 (647,981) | 417.7 (340.1,516.5) | -63.4 (-72.2, -35.9) |
| 134 | Kiribati | 266 (194,331) | 652.8 (488.1,805.7) | 363 (263,464) | 482.1 (360.8,603.2) | -26.2 (-45, -2.3) |
| 135 | Marshall Islands | 163 (103,221) | 974.9 (627.5,1317) | 334 (192,470) | 903.8 (538,1249) | -7.3 (-30.4,24.3) |
| 136 | Micronesia (Federated States of) | 491 (304,654) | 1031.8 (646.3,1349.1) | 682 (352,967) | 938.5 (526.8,1304.2) | -9 (-39.4,26.6) |
| 137 | Nauru | 36 (22,50) | 853.4 (543.3,1136.3) | 37 (20,53) | 758.3 (435.5,1033.6) | -11.1 (-31.8,15.1) |
| 138 | Niue | 13 (9,18) | 599.2 (404.4,816.9) | 10 (6,14) | 467.7 (297.4,637.2) | -21.9 (-41.9,5.6) |
| 139 | Northern Mariana Islands | 49 (33,64) | 254 (171.6,326.1) | 79 (61,96) | 151.6 (120.3,181.7) | -40.3 (-54.2, -15.7) |
| 140 | Palau | 9 (6,12) | 91.5 (65.4,125.2) | 15 (11,19) | 70.3 (54.5,88.5) | -23.2 (-47.4,12) |
| 141 | Papua New Guinea | 11987 (7165,17884) | 629 (379.4,920.4) | 31548 (18380,46386) | 633.3 (381.1,910.9) | 0.7 (-23.1,34.1) |
| 142 | Samoa | 618 (434,793) | 711.3 (502.2,906.1) | 860 (564,1150) | 582.3 (382,767.2) | -18.1 (-40,10.3) |
| 143 | Solomon Islands | 1327 (752,1918) | 876.5 (530.8,1228.9) | 2850 (1480,4363) | 820.8 (462.9,1209.2) | -6.3 (-33.9,25.3) |
| 144 | Tokelau | 9 (6,13) | 714.6 (469.7,1024.6) | 7 (4,9) | 496.3 (328.5,678) | -30.5 (-49.2, -1.1) |
| 145 | Tonga | 110 (79,142) | 199.6 (144.8,256.8) | 138 (103,179) | 173.8 (130.9,224.7) | -12.9 (-36.2,17.1) |
| 146 | Tuvalu | 62 (40,87) | 908.9 (584.5,1251.2) | 67 (42,94) | 660.1 (417,917.2) | -27.4 (-48.1,3.5) |
| 147 | Vanuatu | 459 (293,640) | 674.3 (434.9,916.5) | 1288 (768,1815) | 718.7 (430,994.7) | 6.6 (-24,51.6) |
| 148 | South Asia | 1684231 (998246,2289415) | 334.1 (206.3,452.4) | 3002380 (2193397,3898340) | 229.2 (166.1,296.9) | -31.4 (-44.7, -5) |
| 149 | Bangladesh | 174097 (93721,241139) | 387.8 (212.7,532.6) | 318970 (196059,504659) | 261.4 (162.2,408.9) | -32.6 (-55.9,15.6) |
| 150 | Bhutan | 759 (392,1105) | 346.5 (186.9,509) | 1266 (926,1727) | 243.8 (177.9,327.3) | -29.6 (-55.7,18.9) |
| 151 | India | 1279535 (778867,1793776) | 321.6 (203.3,451.2) | 2255792 (1523567,2999060) | 211.9 (143.3,281.8) | -34.1 (-48.3, -9.5) |
| 152 | Nepal | 24724 (15075,38388) | 284.9 (183.1,464.5) | 47017 (33303,64561) | 232.6 (166.9,319) | -18.3 (-43.3,23.5) |
| 153 | Pakistan | 205117 (121880,275865) | 375.7 (229.8,515.2) | 379336 (241606,499263) | 370.7 (246.9,481.9) | -1.3 (-28.3,25.2) |
| 154 | Southeast Asia | 1232973 (859616,1457858) | 497.9 (358.1,584.1) | 2498936 (1585136,2834243) | 422.9 (274.2,476.8) | -15.1 (-29.6,4.1) |
| 155 | Cambodia | 30326 (17156,41376) | 682.1 (395,913) | 60104 (36908,78070) | 524.5 (328.1,667.6) | -23.1 (-47.7,19.3) |
| 156 | Indonesia | 593699 (365566,744804) | 620.5 (396.2,773.4) | 1162872 (649471,1428830) | 575.8 (334.8,693.7) | -7.2 (-24.1,18.2) |
| 157 | Lao People's Democratic Republic | 15537 (7968,23586) | 769.7 (410.9,1165.8) | 22253 (11820,31403) | 531 (292,744.5) | -31 (-53.1,10.9) |
| 158 | Malaysia | 10255 (8086,12057) | 112.1 (88.4,131.8) | 14965 (11706,18772) | 57.3 (45.1,72.3) | -48.9 (-62.5, -25.8) |
| 159 | Maldives | 194 (84,285) | 232.1 (107.6,336.8) | 339 (264,409) | 122.4 (94.5,147.3) | -47.3 (-66.9,19.4) |
| 160 | Mauritius | 4652 (3572,5098) | 659.1 (514.8,721.1) | 7920 (6227,9899) | 476.4 (374.3,590.4) | -27.7 (-41.1, -8.4) |
| 161 | Myanmar | 166740 (95196,243202) | 720 (427.8,1020.4) | 201225 (117800,267246) | 451.3 (271,593.9) | -37.3 (-55.9, -1) |
| 162 | Philippines | 110662 (98300,129979) | 413.9 (368,480.2) | 557097 (375227,671363) | 726.4 (513.9,866.7) | 75.5 (14.9,116.1) |
| 163 | Seychelles | 773 (635,853) | 1367.7 (1125.6,1509.6) | 846 (731,988) | 806.9 (698.8,938.8) | -41 (-49.7, -19.5) |
| 164 | Sri Lanka | 37657 (33468,43268) | 353.7 (314.1,426) | 77199 (54323,102395) | 311.6 (222.3,410.1) | -11.9 (-42,22) |
| 165 | Thailand | 23795 (18175,29847) | 69.4 (52.3,88.6) | 47579 (36434,61534) | 47.9 (36.7,61.6) | -31 (-49.4, -3.7) |
| 166 | Timor-Leste | 1302 (748,1898) | 500.3 (293.1,731.7) | 4151 (2386,6187) | 536.3 (313.8,791.3) | 7.2 (-27.9,64) |
| 167 | Viet Nam | 235743 (150798,306600) | 607.9 (397.3,786) | 339113 (200811,496994) | 386 (235.5,554.1) | -36.5 (-57.8, -5.2) |
| 168 | Southern Latin America | 112458 (102362,127746) | 251.7 (229.9,292.9) | 175522 (153789,209641) | 207 (182.3,247.8) | -17.8 (-26.8, -7.8) |
| 169 | Uruguay | 5987 (5246,6900) | 153.6 (135.2,176.2) | 8743 (6643,9915) | 149.3 (111.9,169.6) | -2.8 (-25.2,9.6) |
| 170 | Argentina | 86528 (79085,100044) | 275.7 (251.9,324.5) | 121694 (107733,153789) | 221.5 (196.6,280.6) | -19.7 (-27.2, -7.4) |
| 171 | Chile | 19939 (17263,22332) | 218.2 (187.6,242.9) | 45076 (34168,51180) | 188.8 (142.8,214.7) | -13.5 (-35, -2.4) |
| 172 | Southern Sub-Saharan Africa | 169620 (150433,196199) | 608.8 (535.7,712) | 309472 (277895,349063) | 580.3 (519.5,648.9) | -4.7 (-17.5,5.9) |
| 173 | Botswana | 4260 (2882,5908) | 771 (536.5,1057.2) | 9450 (6198,13538) | 720.7 (474.3,1009.5) | -6.5 (-42.1,42.9) |
| 174 | Eswatini | 2544 (1923,3225) | 881.1 (676.8,1102.7) | 5234 (3232,7965) | 940.1 (601.4,1378.5) | 6.7 (-29.4,57.2) |
| 175 | Lesotho | 7570 (5842,9778) | 798.7 (615.7,1018.6) | 13966 (9181,19414) | 1155.5 (776.4,1565.7) | 44.7 (-2.3,111) |
| 176 | Namibia | 5930 (4162,7835) | 853.7 (606.8,1119.7) | 9551 (6335,13812) | 713.1 (488,1002.4) | -16.5 (-43.2,22.1) |
| 177 | South Africa | 132196 (116581,156044) | 614.4 (535.9,744.9) | 243448 (216011,275575) | 574.4 (504.8,647) | -6.5 (-19,3.5) |
| 178 | Zimbabwe | 17120 (13937,23713) | 440.4 (360.6,610.2) | 27822 (18619,45035) | 407.5 (280.2,675.3) | -7.5 (-34.7,30.9) |
| 179 | Tropical Latin America | 370594 (285765,391473) | 415.4 (325.7,440.7) | 572378 (515578,781221) | 240.4 (215.7,327.9) | -42.1 (-47.6, -8.8) |
| 180 | Brazil | 365378 (280511,386086) | 419.8 (328.4,445.5) | 558147 (502265,769793) | 239.8 (215.3,330.2) | -42.9 (-48.3, -9) |
| 181 | Paraguay | 5216 (4394,5844) | 243.7 (202.8,273.4) | 14230 (10785,18224) | 265.6 (200.2,340.5) | 9 (-17.9,41.2) |
| 182 | Western Europe | 721567 (623736,807698) | 121.9 (105.6,136.7) | 1123040 (847281,1253174) | 103.5 (80.7,115.7) | -15.1 (-34, -4.2) |
| 183 | Andorra | 72 (52,99) | 167.6 (121,223.1) | 193 (148,246) | 126.4 (96.7,162.7) | -24.6 (-47.2,7.1) |
| 184 | Austria | 19261 (17180,23781) | 156.4 (139.7,196.3) | 32797 (21287,37460) | 155.7 (104.7,177.1) | -0.4 (-41.5,13.1) |
| 185 | Belgium | 7808 (6851,9838) | 50.3 (44.2,63) | 10228 (8482,13802) | 37.9 (32,52.2) | -24.8 (-34.9, -6.5) |
| 186 | Cyprus | 1359 (819,1779) | 231.6 (130.6,304.6) | 2054 (1270,2419) | 129.1 (73.8,152) | -44.3 (-58.8, -4) |
| 187 | Denmark | 3824 (3051,4356) | 45.9 (36,51.9) | 3753 (3197,4806) | 30.6 (26.3,39.4) | -33.2 (-44.2, -1.5) |
| 188 | Finland | 7428 (6252,8203) | 104.8 (88.1,115.8) | 17739 (9719,20437) | 138.1 (71.9,158.8) | 31.7 (-33.2,52) |
| 189 | France | 71047 (51887,79670) | 80.4 (58.9,89.5) | 86728 (65116,103079) | 48.7 (38.6,58.6) | -39.4 (-46.8, -12.1) |
| 190 | Germany | 283338 (251495,331880) | 218.3 (194.1,256.7) | 373824 (310011,438167) | 166.2 (141.2,205.8) | -23.9 (-34.3, -2.3) |
| 191 | Greece | 16305 (14448,19748) | 110.6 (97.6,134.9) | 34068 (25703,39081) | 117 (90.4,132.4) | 5.7 (-22.6,19.2) |
| 192 | Iceland | 158 (138,198) | 53.4 (46.8,66.7) | 274 (215,323) | 44.1 (35.3,52.1) | -17.4 (-37.7, -4.5) |
| 193 | Ireland | 2378 (2049,2663) | 60.2 (52.2,68.7) | 2670 (2217,3694) | 35.2 (29.2,48) | -41.4 (-50, -13.5) |
| 194 | Israel | 5620 (2948,6392) | 119.3 (63.7,135.4) | 3410 (2653,7796) | 27.8 (21.8,63.6) | -76.7 (-82, -9) |
| 195 | Italy | 167203 (125142,182064) | 186.1 (144.5,203) | 310553 (194450,353300) | 173.1 (111.4,195.5) | -7 (-32.2,1.3) |
| 196 | Luxembourg | 435 (380,544) | 81.6 (72,102.3) | 705 (554,892) | 63.5 (50.7,80.7) | -22.2 (-41.3, -5.3) |
| 197 | Malta | 426 (372,520) | 105.8 (92.3,130.3) | 721 (592,916) | 74.2 (61.3,93.9) | -29.9 (-42.1, -15) |
| 198 | Monaco | 56 (43,74) | 70.9 (56,93.1) | 86 (68,105) | 77.5 (61.3,94.2) | 9.3 (-19,40.9) |
| 199 | Netherlands | 7989 (7163,9737) | 39.6 (35.6,47.8) | 13143 (10357,15218) | 35.9 (29,41.4) | -9.4 (-30.9,2.2) |
| 200 | Norway | 3842 (2897,4229) | 52.7 (40,57.9) | 3007 (2516,4492) | 28 (23.5,42.7) | -46.9 (-53.9, -3) |
| 201 | Portugal | 13281 (9706,14598) | 101.8 (75.3,111.8) | 21445 (14512,24811) | 72.7 (51.6,83.3) | -28.6 (-42.1, -9.3) |
| 202 | San Marino | 27 (22,35) | 84.1 (68.6,107.3) | 71 (51,96) | 91.8 (64.9,126.9) | 9.2 (-28.3,57.1) |
| 203 | Spain | 34370 (30152,42652) | 63.9 (55.8,80.4) | 88610 (53256,103991) | 72.5 (46.4,84.1) | 13.6 (-35.3,30.9) |
| 204 | Sweden | 6307 (5238,9427) | 39.2 (32.8,58.3) | 19729 (9813,23203) | 78.6 (40.7,92.1) | 100.3 (-22,140.2) |
| 205 | Switzerland | 10360 (6342,12455) | 91.4 (57.7,109.1) | 21066 (10189,25154) | 94.9 (50.5,111.5) | 3.8 (-28.3,26.2) |
| 206 | United Kingdom | 58073 (53418,78488) | 64.9 (60.1,84.8) | 75187 (67100,100651) | 60.7 (54.3,74) | -6.6 (-29.5,1.1) |
| 207 | Western Sub-Saharan Africa | 345315 (250285,439743) | 387.9 (287,490.3) | 761582 (475938,972554) | 388.9 (247.3,487.7) | 0.2 (-36.7,26.8) |
| 208 | Benin | 7704 (5577,9656) | 374.5 (276,466.3) | 22492 (13261,32424) | 437.3 (261.7,613.2) | 16.8 (-24.4,64.5) |
| 209 | Burkina Faso | 18189 (12671,24064) | 408.9 (291.2,539.2) | 55844 (30836,75391) | 599.3 (337.2,795.1) | 46.6 (-11.5,97.8) |
| 210 | Cabo Verde | 705 (542,861) | 311.8 (236.9,380) | 1179 (931,1431) | 273.1 (213.3,329.6) | -12.4 (-35.2,20.4) |
| 211 | Cameroon | 18556 (11512,25094) | 401.9 (255.1,541.2) | 52204 (28816,81783) | 409.4 (229.7,625.8) | 1.9 (-35.1,54.1) |
| 212 | Chad | 11206 (7638,15100) | 392.8 (271.6,528) | 29745 (15957,41945) | 497.6 (273.2,685.4) | 26.7 (-16.4,74.8) |
| 213 | C么te d'Ivoire | 14799 (9763,19881) | 346.5 (239.8,453.6) | 47458 (25226,70617) | 419.3 (233.2,607) | 21 (-22,68.2) |
| 214 | Gambia | 1127 (790,1520) | 314.4 (226.4,416.1) | 4814 (2693,7053) | 485.6 (268.4,698.9) | 54.4 (-4.9,132.5) |
| 215 | Ghana | 22882 (15592,30140) | 349.2 (241.9,453.7) | 64347 (37609,86636) | 388.6 (230.9,510.8) | 11.3 (-28.3,51.8) |
| 216 | Guinea | 13749 (8230,18830) | 407.7 (246.7,551.1) | 30531 (15454,42982) | 526.9 (274.8,729.1) | 29.3 (-20.9,89.5) |
| 217 | Guinea-Bissau | 2453 (1152,3356) | 563 (284.9,760.5) | 5274 (2376,7838) | 656.3 (308.1,953.5) | 16.6 (-25.9,70.2) |
| 218 | Liberia | 4079 (2581,5444) | 366.9 (237.3,486.1) | 9546 (4985,14983) | 431.5 (229.8,661.6) | 17.6 (-26.4,70.3) |
| 219 | Mali | 18828 (10922,25550) | 441.3 (262.6,598) | 40825 (23504,54973) | 450.4 (262.6,600.5) | 2.1 (-31.8,38.1) |
| 220 | Mauritania | 4406 (2954,5796) | 432.4 (294.9,571.4) | 7038 (4309,9756) | 335 (206.9,454) | -22.5 (-51.2,8.8) |
| 221 | Niger | 10513 (6529,15109) | 358.4 (225.9,514.7) | 30378 (17044,46019) | 374.9 (214.2,559.1) | 4.6 (-29.8,43.8) |
| 222 | Nigeria | 175001 (120658,244404) | 397.8 (277.5,549.3) | 299011 (189174,410733) | 334.1 (214.8,445.2) | -16 (-50.7,19.9) |
| 223 | Sao Tome and Principe | 127 (96,159) | 200.1 (155.5,248.3) | 201 (142,268) | 183.6 (129.7,237.4) | -8.3 (-33,28.9) |
| 224 | Senegal | 10540 (7564,13213) | 320.5 (232.4,399.2) | 29120 (16855,40025) | 380.4 (222.3,521.7) | 18.7 (-23.4,63.5) |
| 225 | Sierra Leone | 5598 (3789,7669) | 289.4 (199.2,393.4) | 14748 (8423,21385) | 384.4 (221.7,546) | 32.8 (-16.1,85.1) |
| 226 | Togo | 4844 (3619,6098) | 365.6 (278,455.9) | 16817 (9603,23615) | 440.1 (256.2,600.2) | 20.4 (-22.3,68.5) |


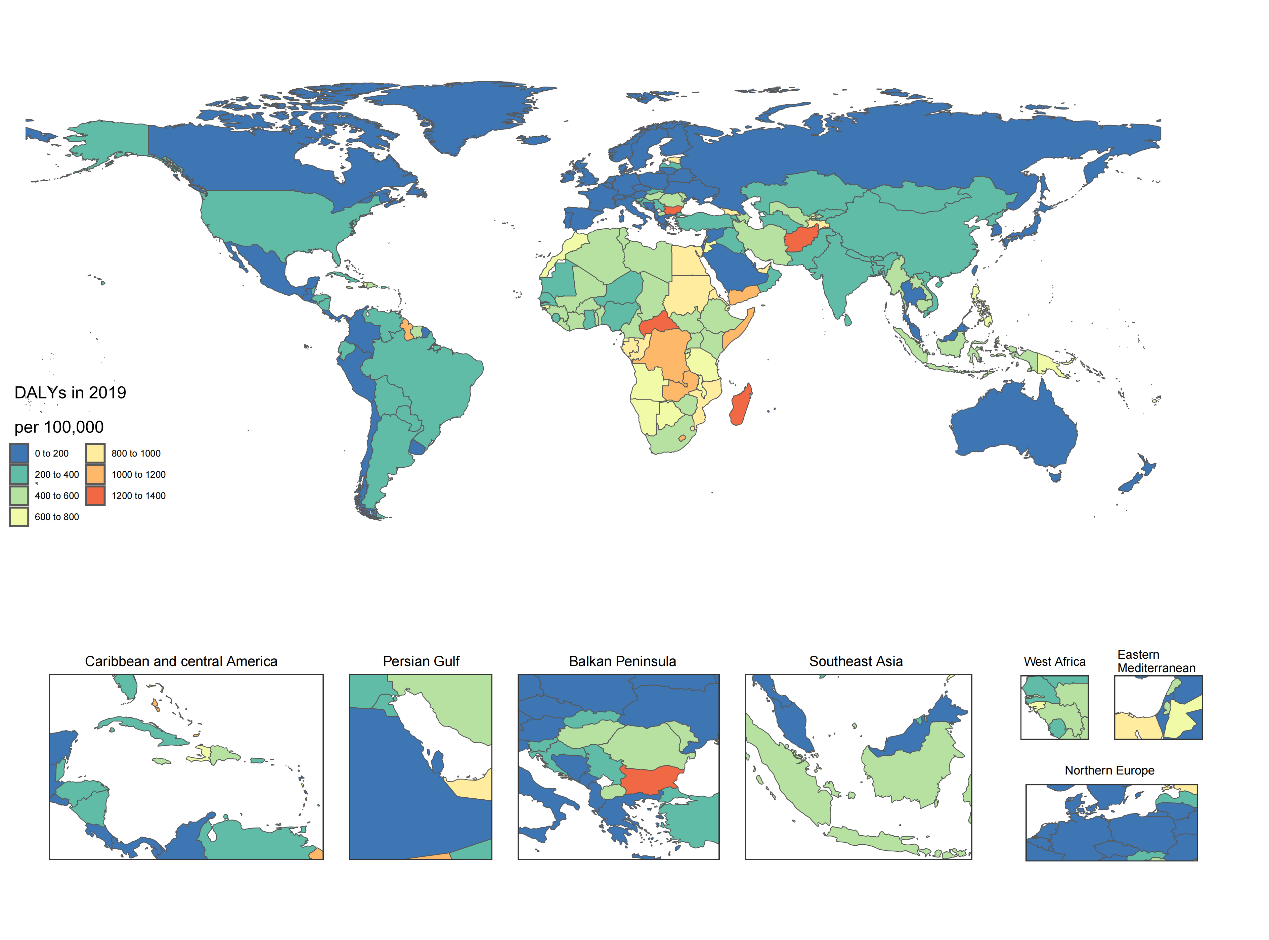


**Figure S1**: Age-standardized DALY rate of hypertensive heart disease per 100,000 people in 2019 by country. DALYs=disability adjusted life years


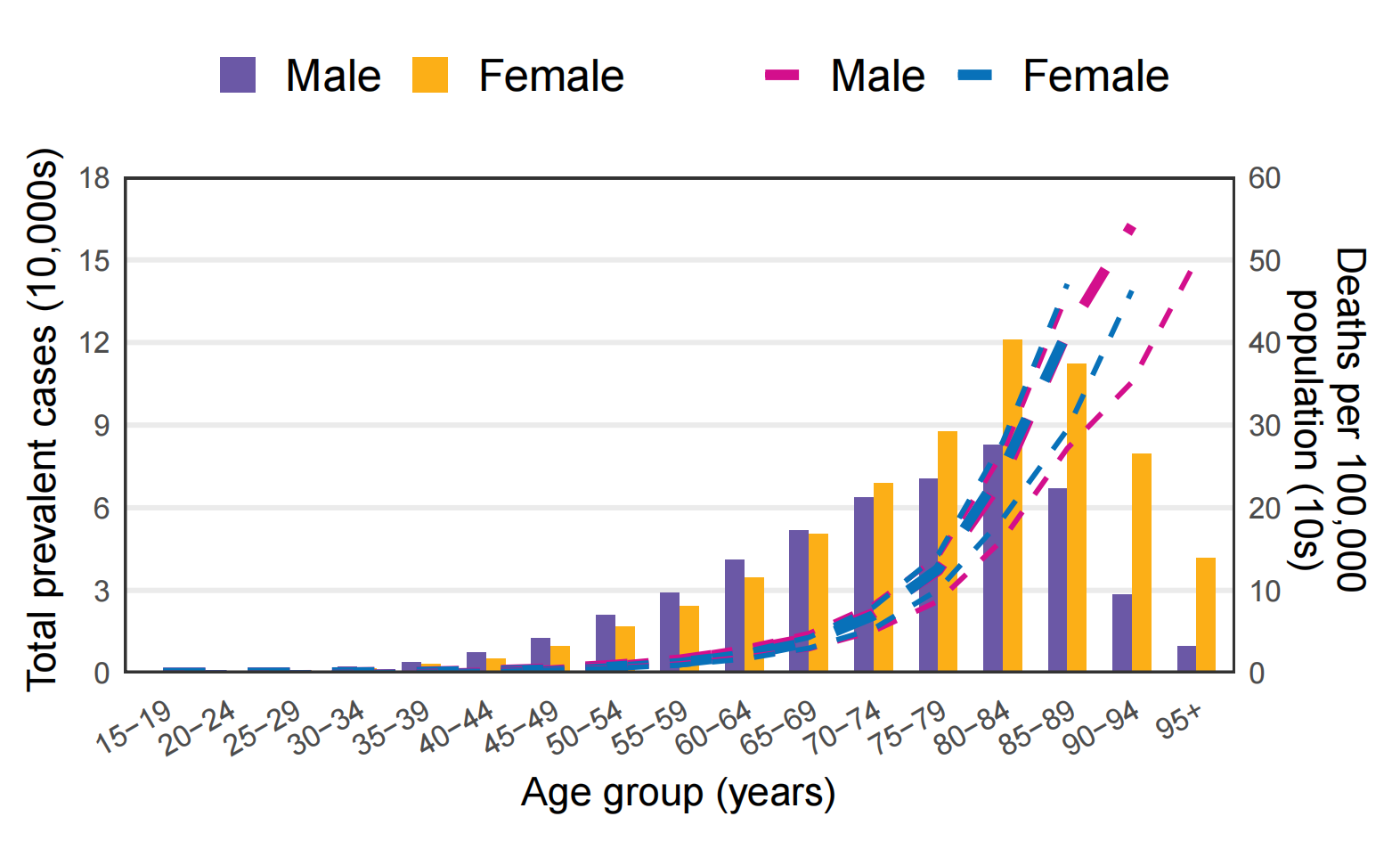


**Figure S2**: Global number of deaths and death rate of hypertensive heart disease per 100,000 people, by age and sex, in 2019; dotted and dashed lines indicate 95% upper and lower uncertainty intervals, respectively


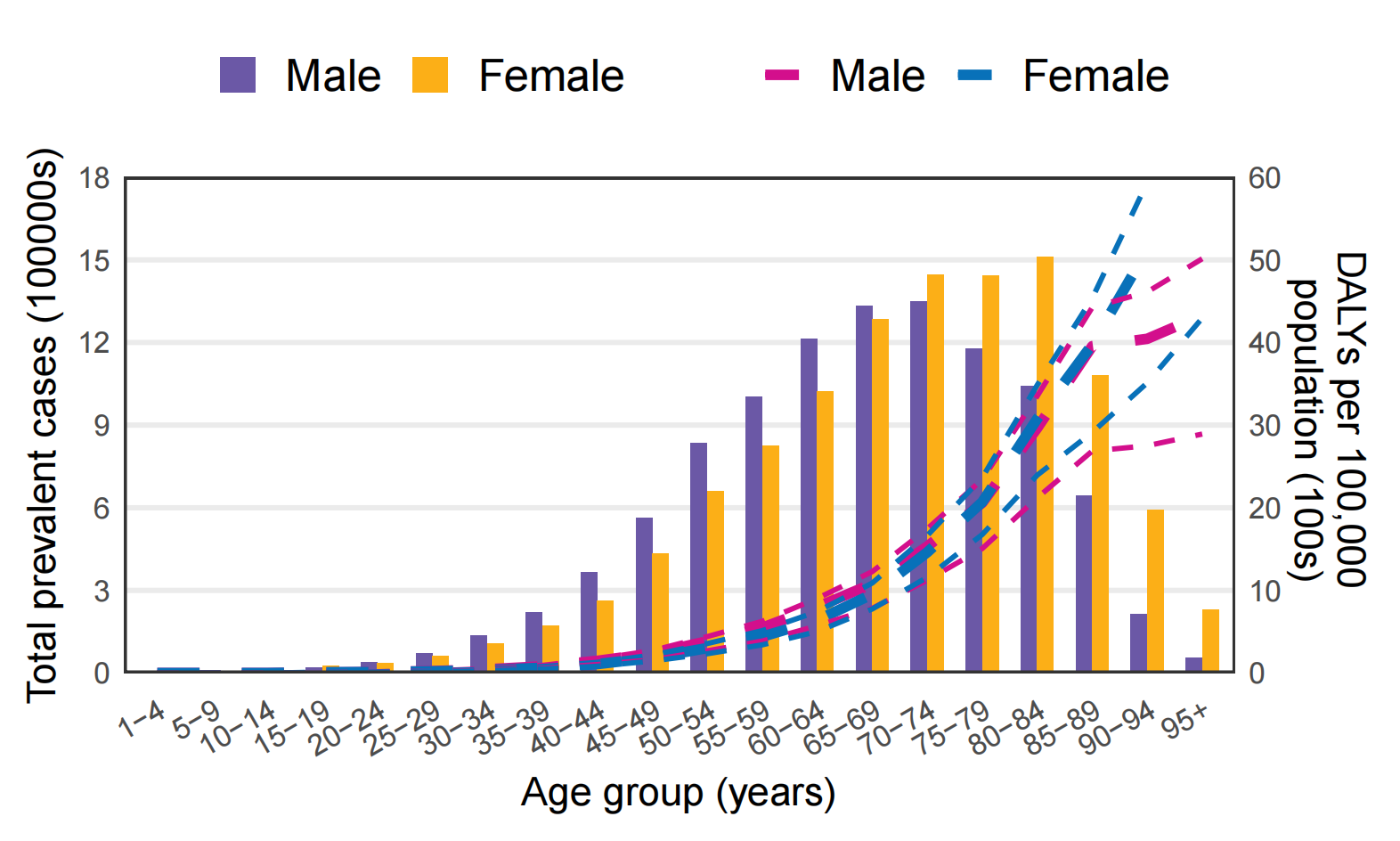


**Figure S3**: Global number of DALYs and DALY rate of hypertensive heart disease per 100,000 people by age and sex in 2019; dotted and dashed lines indicate 95% upper and lower uncertainty intervals, respectively. DALYs=disability adjusted life years


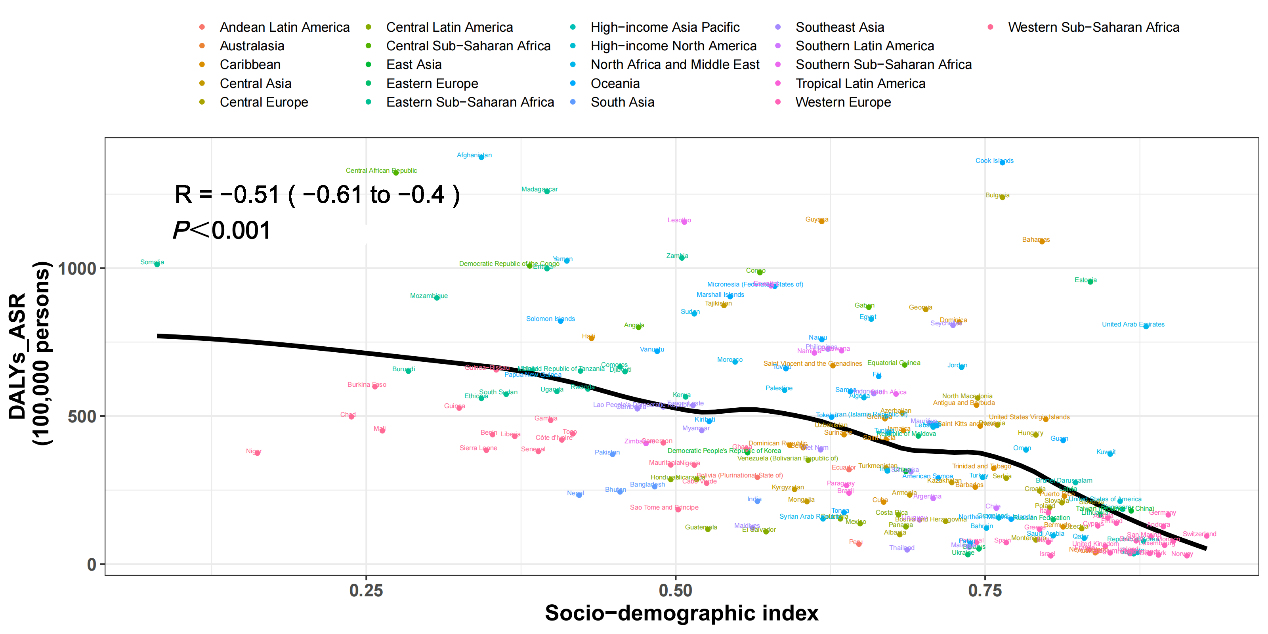


**Figure S4**: Age-standardized DALY rates of hypertensive heart disease for 204 countries and territories by SDI in 2019; expected values based on the sociodemographic index and disease rates in all locations are shown as black lines. Each point shows the observed age-standardized DALY rate for

each country in 2019. DALYs=disability adjusted life years. SDI=

Sociodemographic index


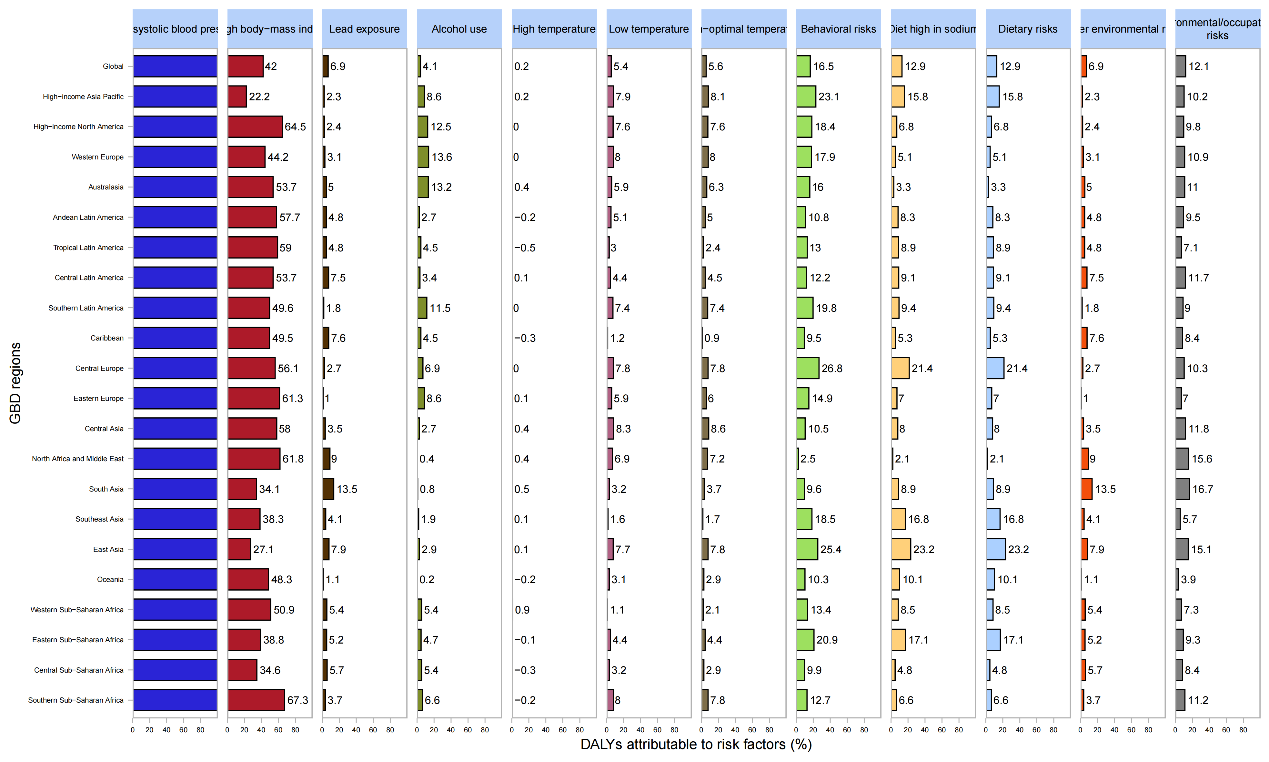


**Figure S5**: Percentage of DALYs due to hypertensive heart disease attributable to risk factors among females in 21 GBD regions in 2019. DALYs=disability adjusted life years
